# A chronological map of 308 physical and mental health conditions from 4 million individuals in the English National Health Service

**DOI:** 10.1016/S2589-7500(19)30012-3

**Published:** 2019-05-20

**Authors:** Valerie Kuan, Spiros Denaxas, Arturo Gonzalez-Izquierdo, Kenan Direk, Osman Bhatti, Shanaz Husain, Shailen Sutaria, Melanie Hingorani, Dorothea Nitsch, Constantinos A Parisinos, R Thomas Lumbers, Rohini Mathur, Reecha Sofat, Juan P Casas, Ian C K Wong, Harry Hemingway, Aroon D Hingorani

**Affiliations:** a Institute of Cardiovascular Science, London; b Health Data Research UK London; c Institute of Health Informatics, University College London, London, UK; d School of Pharmacy, University College London, London, UK; e Alan Turing Institute, London, UK; f Chrisp Street Health Centre, London, UK; g Tower Hamlets Clinical Commissioning Group, London, UK; h Stratford Village Surgery, London, UK; i Clinical Effectiveness Group, Queen Mary University of London, London, UK; j Moorfields Eye Hospital, London, UK; k Department of Non-communicable Disease Epidemiology, London School of Hygiene and Tropical Medicine, London, UK; l Barts Heart Centre, St Bartholomew's Hospital, London, UK; m Massachusetts Veterans Epidemiology Research and Information Center, VA Boston Healthcare System, Boston, MA, USA; n Centre for Safe Medication Practice and Research, Department of Pharmacology and Pharmacy, The University of Hong Kong, Pok Fu Lam, Hong Kong; o National Institute for Health Research, London, UK; p University College London British Heart Foundation Research Accelerator, London, UK

## Abstract

**Background:**

To effectively prevent, detect, and treat health conditions that affect people during their lifecourse, health-care professionals and researchers need to know which sections of the population are susceptible to which health conditions and at which ages. Hence, we aimed to map the course of human health by identifying the 50 most common health conditions in each decade of life and estimating the median age at first diagnosis.

**Methods:**

We developed phenotyping algorithms and codelists for physical and mental health conditions that involve intensive use of health-care resources. Individuals older than 1 year were included in the study if their primary-care and hospital-admission records met research standards set by the Clinical Practice Research Datalink and they had been registered in a general practice in England contributing up-to-standard data for at least 1 year during the study period. We used linked records of individuals from the CALIBER platform to calculate the sex-standardised cumulative incidence for these conditions by 10-year age groups between April 1, 2010, and March 31, 2015. We also derived the median age at diagnosis and prevalence estimates stratified by age, sex, and ethnicity (black, white, south Asian) over the study period from the primary-care and secondary-care records of patients.

**Findings:**

We developed case definitions for 308 disease phenotypes. We used records of 2 784 138 patients for the calculation of cumulative incidence and of 3 872 451 patients for the calculation of period prevalence and median age at diagnosis of these conditions. Conditions that first gained prominence at key stages of life were: atopic conditions and infections that led to hospital admission in children (<10 years); acne and menstrual disorders in the teenage years (10–19 years); mental health conditions, obesity, and migraine in individuals aged 20–29 years; soft-tissue disorders and gastro-oesophageal reflux disease in individuals aged 30–39 years; dyslipidaemia, hypertension, and erectile dysfunction in individuals aged 40–59 years; cancer, osteoarthritis, benign prostatic hyperplasia, cataract, diverticular disease, type 2 diabetes, and deafness in individuals aged 60–79 years; and atrial fibrillation, dementia, acute and chronic kidney disease, heart failure, ischaemic heart disease, anaemia, and osteoporosis in individuals aged 80 years or older. Black or south-Asian individuals were diagnosed earlier than white individuals for 258 (84%) of the 308 conditions. Bone fractures and atopic conditions were recorded earlier in male individuals, whereas female individuals were diagnosed at younger ages with nutritional anaemias, tubulointerstitial nephritis, and urinary disorders.

**Interpretation:**

We have produced the first chronological map of human health with cumulative-incidence and period-prevalence estimates for multiple morbidities in parallel from birth to advanced age. This can guide clinicians, policy makers, and researchers on how to formulate differential diagnoses, allocate resources, and target research priorities on the basis of the knowledge of who gets which diseases when. We have published our phenotyping algorithms on the CALIBER open-access Portal which will facilitate future research by providing a curated list of reusable case definitions.

**Funding:**

Wellcome Trust, National Institute for Health Research, Medical Research Council, Arthritis Research UK, British Heart Foundation, Cancer Research UK, Chief Scientist Office of the Scottish Government Health and Social Care Directorates, Department of Health and Social Care (England), Health and Social Care Research and Development Division (Welsh Government), Public Health Agency (Northern Ireland), Economic and Social Research Council, Engineering and Physical Sciences Research Council, National Institute for Social Care and Health Research, and The Alan Turing Institute.

## Introduction

A chronological map of human health from birth to death depicting the most common conditions by age and marking the median age at diagnosis is fundamental to understanding who gets which conditions when, on a population level. This understanding can inform clinicians about the frequency, and hence the prior probability, of a range of conditions on the basis of the age of presentation. This knowledge could also allow policy makers to consider common conditions at different ages or in different groups when allocating training and resources, and researchers and their funders to prioritise prevalent conditions.

**Research in context**

**Evidence before this study**

We did two English language searches in MEDLINE for studies published in the past 10 years: one search for studies describing disease prevalence for multiple diseases, using the keywords “diseases” OR “disorders”, “epidemiology” OR “prevalence”, AND “comorbidity” OR “multimorbidity”; and the other for studies reporting mean or median age of disease onset or diagnosis using the keywords “diseases” OR “disorders”, AND “age of onset” OR “age at diagnosis” OR “age of diagnosis”, AND “mean” OR “median”.

Consented cohort studies investigating multiple health conditions were limited in age range and in ascertainment of conditions diagnosed in primary care. Many had too few participants to reliably estimate disease distribution by age, sex, and ethnicity. Studies based on electronic health records (EHRs) surmounted these limitations, but the manual curation required for developing case definitions and phenotype algorithms from EHR data restricted the number of conditions analysed within a single study to fewer than 100. Many studies reported prevalence estimates for comorbid conditions relative to an index disease, such as heart failure. Some were confined to either primary or secondary care. The Global Burden of Disease initiative inferred disease prevalence estimates from mathematical models based on empirical frequency data. The US National Cancer Institute's Surveillance, Epidemiology, and End Results cancer statistics review reported age at diagnosis by sex and ethnicity for primary cancer sites. Most other studies reported age at diagnosis for a single disease from small, sometimes unrepresentative sample sets. We did not find any studies that described the age distribution and age at diagnosis stratified by sex and ethnicity from birth to death for several hundred diseases contemporaneously with a single linked clinical dataset obtained from primary-care and secondary-care settings within a universal health-care system.

**Added value of this study**

We present the first lifecourse map of human health, charting the 50 most common conditions in each decade of life, and the median age at diagnosis for 308 conditions. We compiled case definitions, cumulative incidence and age-specific, sex-specific and ethnicity-specific period prevalences for 308 conditions, by harmonising Read, International Classification of Diseases (tenth revision), and Office of the Population Censuses and Surveys Classification of Interventions and Procedures version 4 codes across primary-care and secondary-care records in England. This has involved updating and extensively expanding the phenotyping algorithms in the CALIBER Portal. Conditions were selected to reflect the disease burden and health-care utilisation of the English population, which are likely to be similar to those in countries with similar economies and population structures. Conditions with more than 10 000 Hospital Episode Statistics finished consultant episodes (the time spent under the care of one consultant while admitted to hospital) in England from April 1, 2014, to March 31, 2015, or those with estimated prevalences greater than 0·01% and considered clinically important by our panel of clinicians were included in this report.

Our results illustrate the varying dominance of different conditions through the passage of life. Common childhood conditions were atopic disorders and acute infections. Acne and menstrual disorders gained prominence in teenagers. Mental health disorders emerged in young adults, together with obesity and migraine. Disorders associated with the metabolic syndrome, soft tissue disorders, erectile dysfunction, and gastro-oesophageal reflux disease rose substantially in middle-age. Cancer, osteoarthritis, benign prostatic hyperplasia, cataract, diverticular disease, and deafness became more common in individuals aged 60–79 years, whereas atrial fibrillation, dementia, acute and chronic kidney disease, heart failure, ischaemic heart disease, anaemia, and osteoporosis escalated in advanced age (≥80 years).

Ethnic and sex differences were also discernible. White patients had later median age at diagnosis for 258 of the 308 conditions. Although this could be attributed to the older age structure of the white population, another potential reason is that distinct biological pathways can lead to the same diagnosis in different demographic groups. Sleep apnoea, for example, was common in black boys and older white men, with potentially different mechanisms underlying the two groups. Female individuals were younger at diagnosis of tubulointerstitial nephritis, urinary incontinence, chronic cystitis, and nutritional anaemias, whereas male individuals were diagnosed at younger ages with bone fractures and atopic conditions.

**Implications of all the available evidence**

By mapping the distribution of health conditions across the lifecourse, we have empowered researchers, clinicians, health-care providers, and policy makers to better identify individuals at risk, and to instigate strategies to detect, prevent, and manage specific conditions. The patterns of disease distribution that we have revealed could lead to further research into the heterogeneous causes of diseases. The platform that we have created can promote further research into ageing-related health conditions and multimorbidity to meet the challenges facing ageing populations. By providing the phenotyping algorithms for hundreds of conditions through an existing open access Portal (CALIBER), we are also facilitating the use of EHR data in large cohort studies such as UK Biobank in this era of high-throughput biomedical data.

Addressing this question requires large-scale, population-based studies with broad coverage of health conditions and appropriate age-related frequency measures. The age-specific cumulative incidence establishes when specific conditions are more likely to occur during the lifecourse, while the age-specific period prevalence unveils the collective past medical history of a population at each stage of life over a specified calendar time period. Age at first recorded diagnosis by sex and ethnicity characterises patterns of health-condition onset by age and differences between demographic groups, with potentially distinctive underlying pathological processes. Although the Global Burden of Disease (GBD) reports^1^ and multimorbidity studies^2^, ^3^, ^4^ have drawn from various data sources to estimate disease frequency statistics for the overall population, there have been no previous studies linking prevalence estimates with age of diagnosis for multiple conditions in parallel within a single health system, to draw the chronological map of human health conditions across the lifecourse.

The UK National Health Service (NHS) is well placed to support these analyses, as the provider of universal cradle-to-grave health care in the UK since 1948, with more than 98% of the UK population registered with an NHS general practice.^5^ NHS clinical data can be aggregated on a population scale, with electronic health records (EHRs) in primary care^6^ linked to digitised disease-episode coding in secondary care^7^ using unique NHS identification numbers assigned permanently to individuals.^8^ EHRs comprise data from multiple sources with a variety of coding schemes, wherein a single condition such as type 2 diabetes might be represented by hundreds of codes. Therefore, the construction of case definitions and codelists across the various clinical settings requires meticulous curation, which has previously been a limiting factor in the contemporaneous study of hundreds of conditions.

We have created a chronological map of human health by charting the most common mental and physical health conditions by decade of age, and by estimating the median age at first recorded diagnosis for 308 health conditions using linked EHRs in England. We have also compiled a compendium of phenotyping algorithms and codelists; age-specific, sex-specific, and ethnicity-specific prevalences; and median age at first record by sex and ethnicity of the spectrum of disorders affecting recipients of NHS care for the use of clinicians, policy makers, health-care providers, and researchers.

## Methods

### Study design and participants

We studied population-based EHRs of primary-care patient-level data from the Clinical Practice Research Datalink (CPRD) linked to the dataset of the Hospital Episode Statistics (HES) for admitted-patient care. CPRD is one of the largest EHR databases in the world, is representative of the English population by age, sex, and ethnicity,^5^, ^9^ provides anonymised data, and has been previously validated for epidemiological research.^10^ Individuals older than 1 year were included in the study if their records met research standards set by the CPRD^5^ and they had been registered in a general practice in England contributing up-to-standard data for at least 1 year from April 1, 2010, to March 31, 2015.

The study was approved by the Independent Scientific Advisory Committee for the Medicines and Healthcare products Regulatory Agency (protocol 16_022).

### Procedures

We identified physical and mental health conditions that involve intensive use of health-care resources. These conditions included those from the quality and outcomes framework,^11^ a UK general-practice payment-for-performance scheme, with modifications for more granular phenotypes that reflect distinct pathological pathways—where applicable—such as type 1 diabetes, type 2 diabetes, and diabetes (other or unspecified).

The number of finished consultant episodes (the time spent by an inpatient under the care of one consultant) for all diagnoses in England from April 1, 2014, to March 31, 2015, was obtained from inpatient activity reports published by NHS Digital.^12^ Diagnoses were coded using three-character or four-character codes from the International Classification of Diseases, tenth revision (ICD-10). We examined the finished consultant episodes for codes in chapters I–XIV and XVI–XVII of the ICD-10. We excluded pregnancy-related conditions, symptoms, signs, abnormal clinical and laboratory findings, and external causes of morbidity and mortality. Three-character or four-character ICD-10 codes were assigned to specific conditions as agreed between clinicians in the team (VK, OB, SS, SH, MH, DN, CAP, RTL, RS, and ADH). Conditions with codes that had more than 10 000 finished consultant episodes were included. If a condition had fewer than 10 000 finished consultant episodes but the prevalence was greater than 0·01% and it was considered to be clinically important by our panel, it was included in the study (appendix p 56).

Infections were categorised by organ system and causal organism. Chronic infections with long-term sequelae included were HIV, chronic viral hepatitis, tuberculosis, and rheumatic fever. Acute infections were limited to hospital admissions. Obesity was only considered for individuals older than 18 years.

Health conditions were harmonised across primary-care and secondary-care coding systems and organised into 16 disease categories corresponding closely to ICD-10 chapters (appendix pp 2–6).

Phenotyping algorithms defining these conditions were based on diagnosis or procedural codes, with the additional inclusion of some blood test values or other measures—ie, estimated glomerular filtration rate, total cholesterol, low-density lipoprotein cholesterol, high-density lipoprotein cholesterol, triglyceride, or body-mass index (BMI). Diagnoses and procedures are recorded in CPRD with Read codes. ICD-10 diagnosis codes and Office of the Population Censuses and Surveys Classification of Interventions and Procedures version 4 (OPCS-4) procedural codes are used in the HES for admitted-patient care. Keywords were searched in the Read and OPCS-4 dictionaries for each of the selected conditions to construct the Read and OPCS-4 codelists. Patients were considered to have or have had a specific condition if they met the criteria in the algorithm for that condition before or during the study period. Algorithms and codelists for all identified conditions are available on the CALIBER Portal. The algorithms can be downloaded in a machine-readable CSV format from the algorithm data repository.

Selection of health conditions, algorithm development, and codelist construction were done by a panel of clinicians with expertise spanning the range of recorded conditions (VK, OB, SH, SS, MH, DN, CAP, RTL, RS, and ADH).

The main outcomes of our study were cumulative incidence and period prevalence, stratified by age, ethnicity, and sex (male and female), and age at first diagnosis.

Ethnicity was grouped into the five categories of the 2011 UK census—ie, white, mixed, south Asian, black, and other (appendix pp 7).^9^ Patients with missing ethnicity or codes belonging to more than one category were classified as unknown. Ethnic stratification was reported for white, south-Asian, and black populations only, as interpretation of mixed and other populations is less meaningful when considering disease susceptibility.

### Statistical analysis

The age at first recorded diagnosis was the earliest age at which the criteria in a phenotyping algorithm for a specific condition were met from any source.

The cumulative incidence between April 1, 2010, and March 31, 2015, was calculated by dividing the number of incident cases (people with first recorded diagnoses) during this time period by the number of people in the study population at risk on April 1, 2010. We computed the sex-standardised cumulative incidence for 10-year age bands (0–9 years, 10–19 years, 20–29 years, 30–39 years, 40–49 years, 50–59 years, 60–69 years, 70–79 years, ≥80 years). As we had not estimated the prevalence of childhood obesity in this study, we did not calculate the cumulative incidence for obesity for those between 18 years and 20 years of age because we were unable to determine the denominator (individuals aged 18 years on April 1, 2010, who had not previously been defined as obese). Age-specific, sex-specific, and ethnicity-specific period prevalences from April 1, 2010, to March 31, 2015, were calculated by dividing the number of new and pre-existing cases by the number of people in the study population during this time period. Standardisation was applied using the 2013 European Standard Population.^13^ The median age (IQR) at which conditions were first recorded was determined for patients in the study population.

We compared our prevalence estimates with those from the GBD study^1^ and from Barnett and colleagues' study.^2^ Prevalence estimates were obtained directly from the published article in the case of Barnett and colleagues' study,^2^ or downloaded from the GBD online results tool^14^ in the case of the GBD 2017 study.^1^

Analyses were done using R (version 3.4.3).

### Role of the funding source

The funders had no role in study design, data collection, data analysis, data interpretation, report writing, or the decision to submit the paper for publication. The corresponding author had full access to all the data in the study and had final responsibility for the decision to submit for publication.

## Results

We developed case definitions for 308 disease phenotypes from 10 819 Read codes, 1932 ICD-10 diagnosis codes, 670 OPCS-4 procedural codes, and measurements of estimated glomerular filtration rate, total cholesterol, low-density lipoprotein cholesterol, high-density lipoprotein cholesterol, triglyceride, and BMI. Cumulative-incidence estimates from April 1, 2010, to March 31, 2015, were calculated from records of 2 784 138 individuals at the start of the study period on April 1, 2010. Period-prevalence estimates for the same period and the median age at first record were derived from 3 872 451 individuals in the study population. 2 666 234 (68·9%) of 3 872 451 individuals were white, 860 275 (22·2%) had unknown ethnicity, 155 435 (4·0%) were of south-Asian ethnicity, 98 815 (2·6%) were black, 33 673 (0·9%) had mixed ethnicity, and 58 019 (1·5%) were of other ethnicity; this ethnicity distribution was represented across age and sex subgroups (table).

**Table:** Number of individuals in each ethnic group in the study population from April 1, 2010, to March 31, 2015, stratified by age and sex

	**South Asian (n=155 435)**	**Black (n=98 815)**	**Mixed (n=33 673)**	**Other (n=58 019)**	**White (n=2 666 234)**	**Unknown (n=860 275)**	**All ethnicities (n=3 872 451)**
**Sex**							
Female	78 056 (50·2%)	51 943 (52·6%)	17 172 (51·0%)	30 367 (52·3%)	1 422 425 (53·3%)	355 737 (41·4%)	1 955 700 (50·5%)
Male	77 379 (49·8%)	46 872 (47·4%)	16 501 (49·0%)	27 652 (47·7%)	1 243 809 (46·7%)	504 538 (58·6%)	1 916 751 (49·5%)
**Age groups, both sexes (years)**							
0–9 (n=542 337)	32 971 (6·1%)	21 739 (4·0%)	14 653 (2·7%)	9651 (1·8%)	384 691 (70·9%)	78 632 (14·5%)	..
10–19 (n=433 169)	15 811 (3·7%)	13 059 (3·0%)	5101 (1·2%)	7214 (1·7%)	250 517 (57·8%)	141 467 (32·7%)	..
20–29 (n=546 371)	32 198 (5·9%)	14 866 (2·7%)	5273 (1·0%)	11 224 (2·1%)	345 175 (63·2%)	137 635 (25·2%)	..
30–39 (n=546 596)	33 404 (6·1%)	18 203 (3·3%)	3935 (0·7%)	12 055 (2·2%)	347 786 (63·6%)	131 213 (24·0%)	..
40–49 (n=558 963)	17 387 (3·1%)	16 756 (3·0%)	2518 (0·5%)	8206 (1·5%)	365 120 (65·3%)	148 976 (26·7%)	..
50–59 (n=443 489)	11 339 (2·6%)	7593 (1·7%)	1187 (0·3%)	4834 (1·1%)	304 502 (68·7%)	114 034 (25·7%)	..
60–69 (n=379 796)	6638 (1·7%)	3158 (0·8%)	548 (0·1%)	2774 (0·7%)	295 806 (77·9%)	70 872 (18·7%)	..
70–79 (n=244 823)	4237 (1·7%)	2642 (1·1%)	315 (0·1%)	1382 (0·6%)	209 890 (85·7%)	26 357 (10·8%)	..
≥80 (n=176 907)	1450 (0·8%)	799 (0·5%)	143 (0·1%)	679 (0·4%)	162 747 (92·0%)	11 089 (6·3%)	..
All ages (n=3 872 451)	155 435 (4·0%)	98 815 (2·6%)	33 673 (0·9%)	58 019 (1·5%)	2 666 234 (68·9%)	860 275 (22·2%)	..

**Figure 1:**
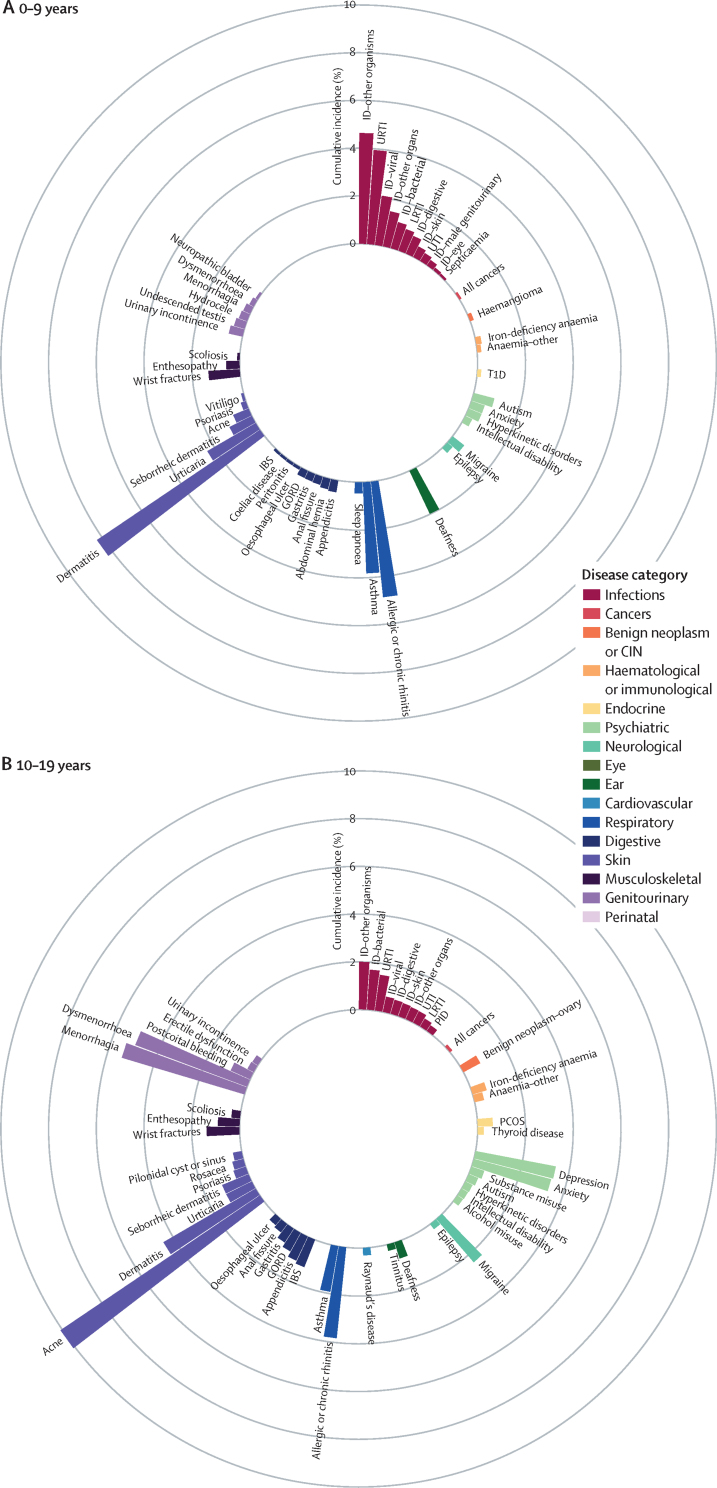

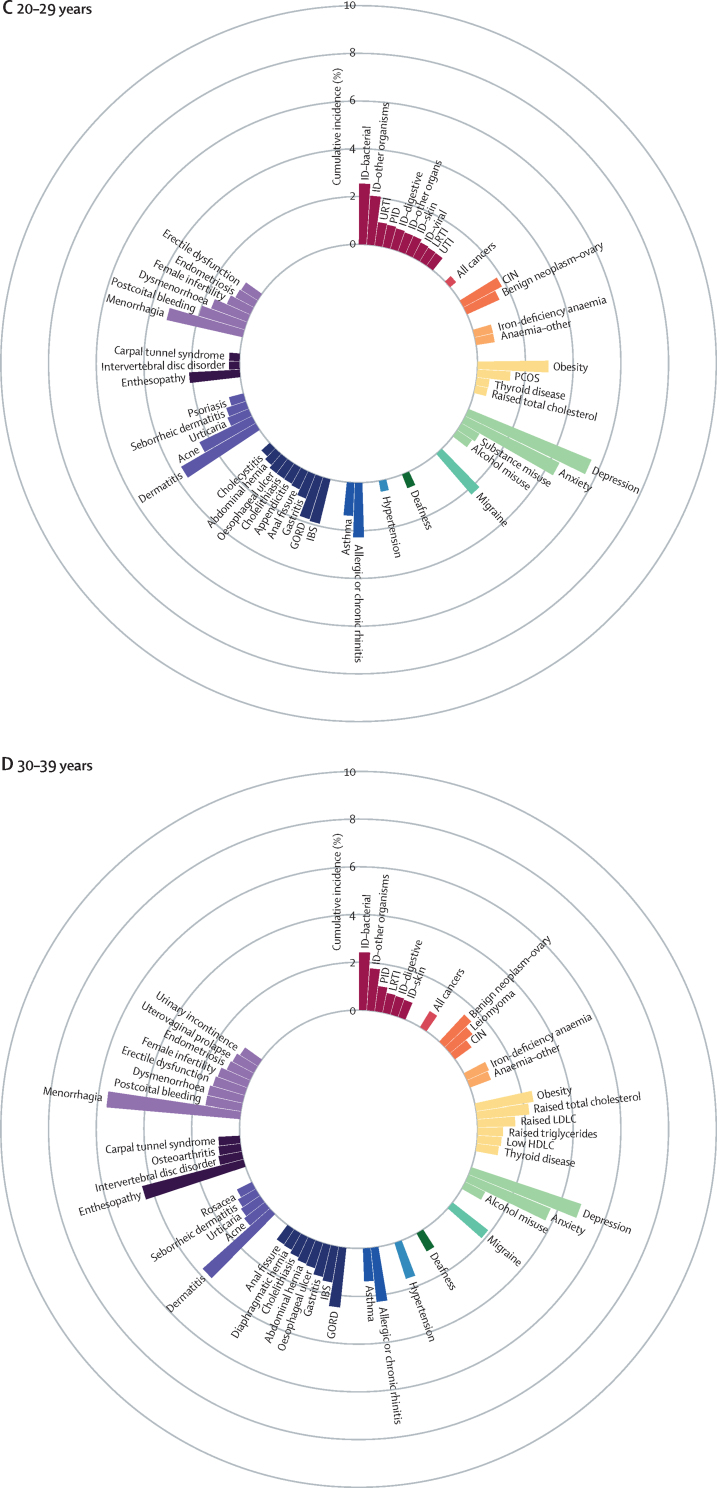

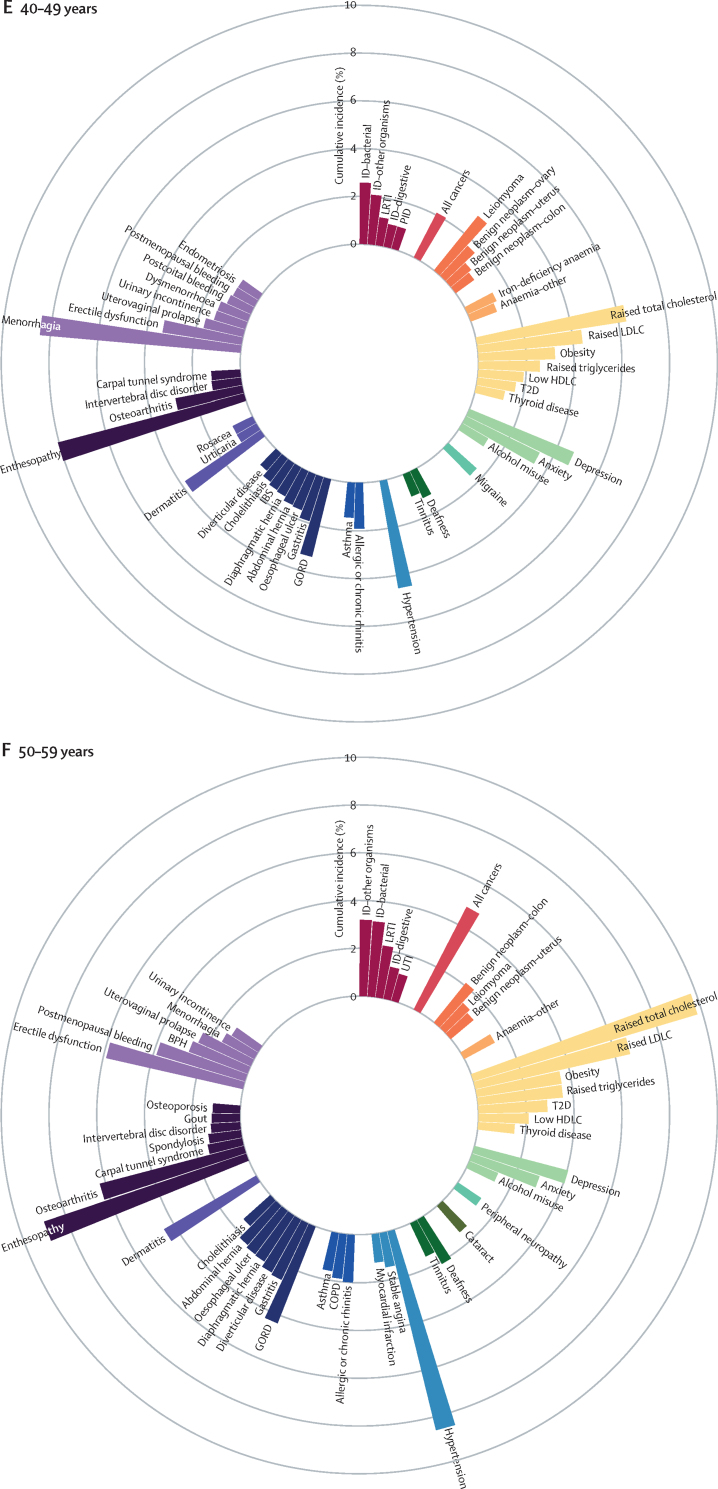

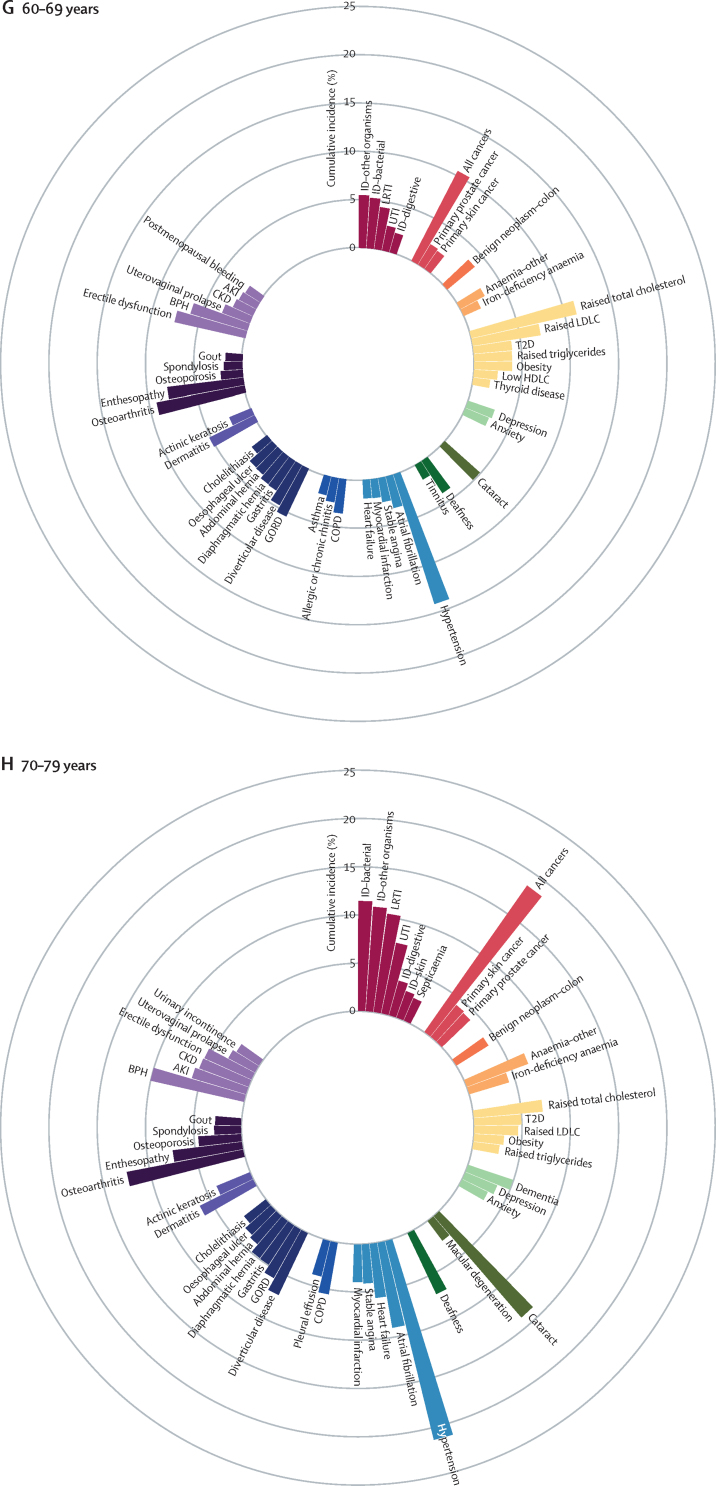

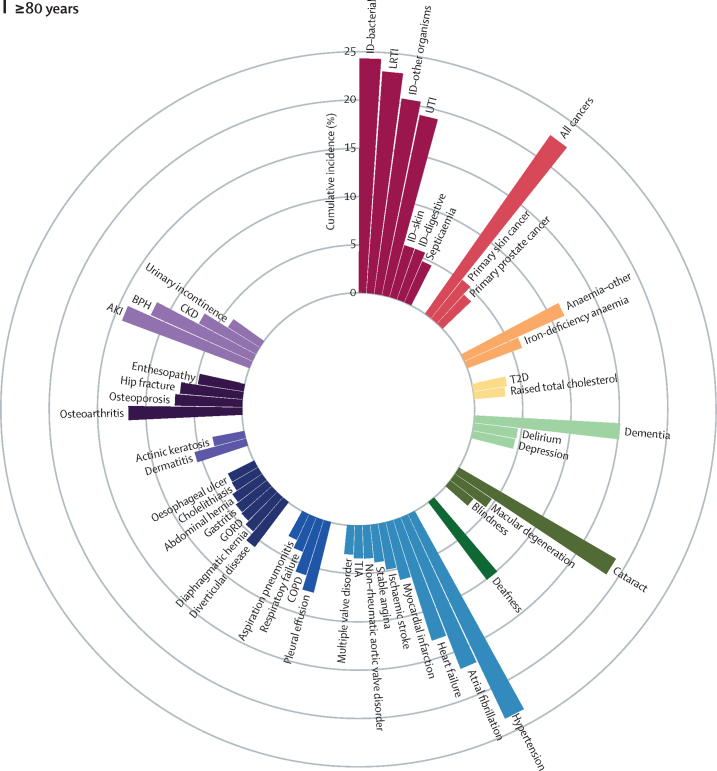
Sex-standardised cumulative incidence (%) of the top 50 diseases and all cancers between April 1, 2010, and March 31, 2015, for individuals aged 1–9 years (A), 10–19 years (B), 20–29 years (C), 30–39 years (D), 40–49 years (E), 50–59 years (F), 60–69 years (G), 70–79 years (H), or 80 years or more (I) on April 1, 2010 AKI=acute kidney injury. BPH=benign prostatic hyperplasia. CIN=cervical intraepithelial neoplasia. CKD=chronic kidney disease. COPD=chronic obstructive pulmonary disease. GORD=gastro-oesophageal reflux disease. HDLC=high-density lipoprotein cholesterol. IBS=irritable bowel syndrome. ID=infectious disease. LDLC=low-density lipoprotein cholesterol. LRTI=lower respiratory tract infection. PCOS=polycystic ovary syndrome. PID=pelvic inflammatory disease. T1D=type 1 diabetes. T2D=type 2 diabetes. TIA=transient ischaemic attack. URTI=upper respiratory tract infection. UTI=urinary tract infection.

We identified the most common conditions in each phase of life for individuals in the study population during the study period (figure 1; appendix pp 8–16 and 58–60)

The most common childhood conditions in children younger than 10 years were dermatitis (cumulative incidence 8·23%, 95% CI 8·10–8·37; period prevalence 33·12%, 32·96–33·27), allergic rhinitis (4·86%, 4·77–4·94; 6·39%, 6·33–6·46), infections from other or unspecified organisms (ie, no record satisfying criteria for bacterial diseases, tuberculosis, viral diseases, chronic viral hepatitis, HIV, mycoses or parasitic infections 30 days before or 30 days after the first event date; 4·63%, 4·54–4·72; 14·16%, 14·06–14·26), infections of the upper respiratory tract (3·94%, 3·86–4·02; 12·09%, 12·00–12·18), and asthma (3·81%, 3·73–3·89; 7·98%, 7·90–8·05%; figure 1A; appendix pp 8–16 and 58–60).

Acne (cumulative incidence 10·08%, 95% CI 9·96–10·19; period prevalence 17·20%, 17·07–17·32), menorrhagia (5·36%, 5·24–5·48; 8·46%, 8·33–8·58), and dysmenorrhoea (4·99%, 4·88–5·10; 9·03%, 8·90–9·15) were common in individuals aged 10–19 years (figure 1B; appendix pp 8–16 and 58–60).

Depression (cumulative incidence 5·56%, 95% CI 5·47–5·64; period prevalence 15·05%, 14·95–15·15), anxiety (4·38%, 4·31–4·46; 11·67%, 11·58–11·76), obesity (2·96%, 2·89–3·02; 9·53%, 9·45–9·61), and migraine (2·23%, 2·18–2·29; 8·30%, 8·22–8·37) began to emerge from young adulthood (ie, individuals aged 20–29 years; figure 1C; appendix pp 8–16 and 58–60).

Enthesopathy and synovial disorders (cumulative incidence 4·38%, 95% CI 4·31–4·45; period prevalence 11·21%, 11·12–11·30) and gastro-oesophageal reflux disease (2·50%, 2·45–2·55; 6·08%, 6·02–6·15) began to feature more prominently in individuals aged 30–39 years (figure 1D; appendix pp 8–16 and 58–60).

For individuals aged 40–59, raised total cholesterol (cumulative incidence 6·32%, 95% CI 6·24–6·40 [for the 40–49 years age range], and 9·79%, 9·66–9·91 [for the 50–59 years age range]; period prevalence 22·05%, 21·92–22·17 [for the 40–49 years age range], and 41·72%, 41·53–41·91 [for the 50–59 years age range]), hypertension (cumulative incidence 4·55%, 4·49–4·62 [for the 40–49 years age range], and 8·56%, 8·45–8·66 [for the 50–59 years age range]; period prevalence 11·80%, 11·71–11·89 [for the 40–49 years age range], and 26·55%, 26·40–26·70 [for the 50–59 years age range]), raised low-density lipoprotein cholesterol (cumulative incidence 4·39%, 4·33–4·46 [for the 40–49 years age range], and 6·64%, 6·54–6·73 [for the 50–59 years age range]; period prevalence 14·52%, 14·42–14·62 [for the 40–49 years age range], and 27·46%, 27·30–27·61 [for the 50–59 years age range]), and erectile dysfunction (cumulative incidence 3·34%, 3·26–3·42 [for the 40–49 years age range], and 5·86%, 5·75–5·97 [for the 50–59 years age range]; period prevalence 8·14%, 8·04–8·25 [for the 40–49 years age range], and 15·51%, 15·35–15·68 [for the 50–59 years age range]) acquired increased importance (figure 1E–F; appendix pp 8–16 and 58–60).

Conditions that gained prominence among individuals aged 60–79 years were all cancers (cumulative incidence 10·44%, 95% CI 10·32–10·56 [for the 60–69 years age range], and 18·34%, 18·13–18·54 [for the 70–79 years age range]; period prevalence 18·63%, 18·50–18·77 [for the 60–69 years age range], and 30·02%, 29·80–30·24 [for the 70–79 years age range]), cataract (cumulative incidence 4·85%, 4·77–4·92 [for the 60–69 years age range], and 13·73%, 13·56–13·90 [for the 70–79 years age range]; period prevalence 9·46%, 9·36–9·56 [for the 60–69 years age range], and 27·55%, 27·34–27·75 [for the 70–79 years age range]), osteoarthritis (cumulative incidence 9·26%, 9·14–9·38 [for the 60–69 years age range], and 12·14%, 11·96–12·32 [for the 70–79 years age range]; period prevalence 28·68%, 28·51–28·85 [for the 60–69 years age range], and 41·88%, 41·63–42·14 [for the 70–79 years age range]), benign prostatic hyperplasia (cumulative incidence 6·13%, 6·00–6·26 [for the 60–69 years age range], and 9·81%, 9·59–10·03 [for the 70–79 years age range]; period prevalence 16·36%, 16·18–16·54 [for the 60–69 years age range], and 30·99%, 30·66–31·31 [for the 70–79 years age range]), diverticular disease (cumulative incidence 4·74%, 4·66%–4·81% [for the 60–69 years age range], and 6·99%, 6·87–7·10 [for the 70–79 years age range]; period prevalence 9·92%, 9·82–10·02 [for the 60–69 years age range], and 17·17%, 17·00–17·33 [for the 70–79 years age range]), type 2 diabetes (cumulative incidence 3·98%, 3·90–4·05 [for the 60–69 years age range], and 4·83%, 4·73–4·93 [for the 70–79 years age range]; period prevalence 13·23%, 13·11–13·34 [for the 60–69 years age range], and 19·36%, 19·19–19·54 [for the 70–79 years age range]), and deafness (cumulative incidence 3·94%, 3·87–4·01 [for the 60–69 years age range], and 6·89%, 6·77–7·01 [for the 70–79 years age range]; period prevalence 12·85%, 12·74–12·96 [for the 60–69 years age range], and 20·94%, 20·75–21·12 [for the 70–79 years age range]; figure 1G–H and appendix pp 8–16 and 58–60).

Atrial fibrillation (cumulative incidence 16·78%, 95% CI 16·55–17·01; period prevalence 32·29%, 32·02–32·56), dementia (15·03%, 14·84–15·24; 24·51%, 24·29–24·74)), acute kidney injury (14·02%, 13·83–14·21; 16·44%, 16·25–16·63), heart failure (13·04%, 12·85–13·24; 23·18%, 22·96–23·41), anaemia (other or unspecified [not iron-deficiency anaemia, B12-deficiency anaemia, folate-deficiency anaemia, thalassaemia, thalassaemia trait, sickle cell anaemia, other haemolytic anaemia, or aplastic anaemia]; 11·30%, 11·12–11·48; 21·94%, 21·72–22·16), osteoporosis (6·94%, 6·81–7·08; 17·94%, 17·76–18·13), chronic kidney disease (6·65%, 6·50–6·80; 32·09%, 31·83–32·35), hip fracture (6·47%, 6·34–6·60; 12·43%, 12·27–12·58), and myocardial infarction (6·12%, 5·99–6·25; 16·70%, 16·50–16·90) contributed substantially to the disease burden in advanced age (ie, ≥80 years; figure 1I; appendix pp 8–16 and 58–60).

Age-standardised period-prevalence estimates for the 308 conditions stratified by sex and ethnicity are provided in the appendix (pp 17–26).

**Figure 2:**
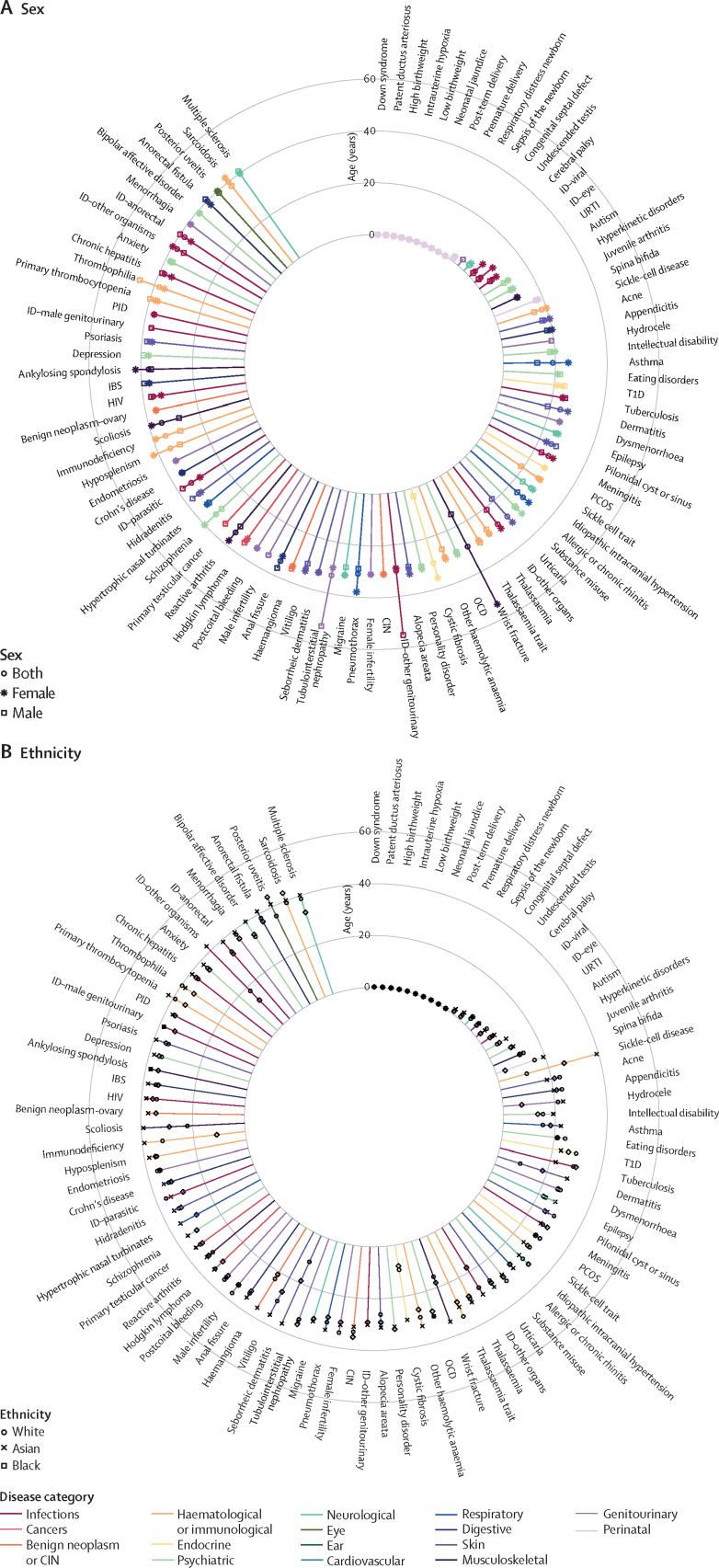
Median age at first record for diseases with median age of diagnosis at or before 40 years, stratified by sex (A) and ethnicity (B) CIN=cervical intraepithelial neoplasia. IBS=irritable bowel syndrome. ID=infectious disease. OCD=obsessive-compulsive disorder. PCOS=polycystic ovary syndrome. PID=pelvic inflammatory disease. T1D=type 1 diabetes. URTI=upper respiratory tract infection.

**Figure 3:**
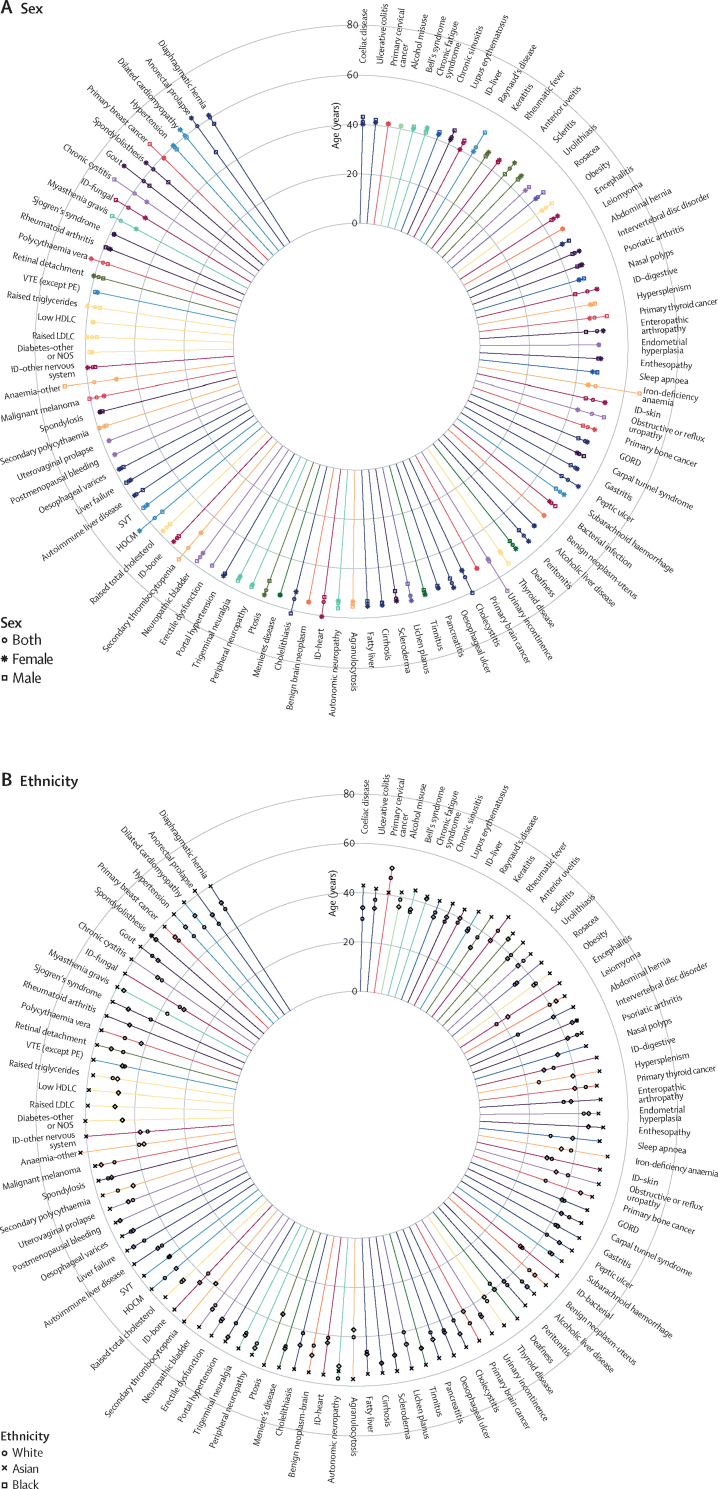
Median age at first record for diseases with median age of diagnosis between 40–60 years, stratified by sex (A) and ethnicity (B) For the key for the disease categories refer to figure 2. GORD=gastro-oesophageal reflux disease. HDLC=high-density lipoprotein cholesterol. HOCM=hypertrophic obstructive cardiomyopathy. ID=infectious disease. LDLC=low-density lipoprotein cholesterol. NOS=not otherwise specified. PE=pulmonary embolism. SVT=supraventricular tachycardia. VTE=venous thromboembolism.

**Figure 4:**
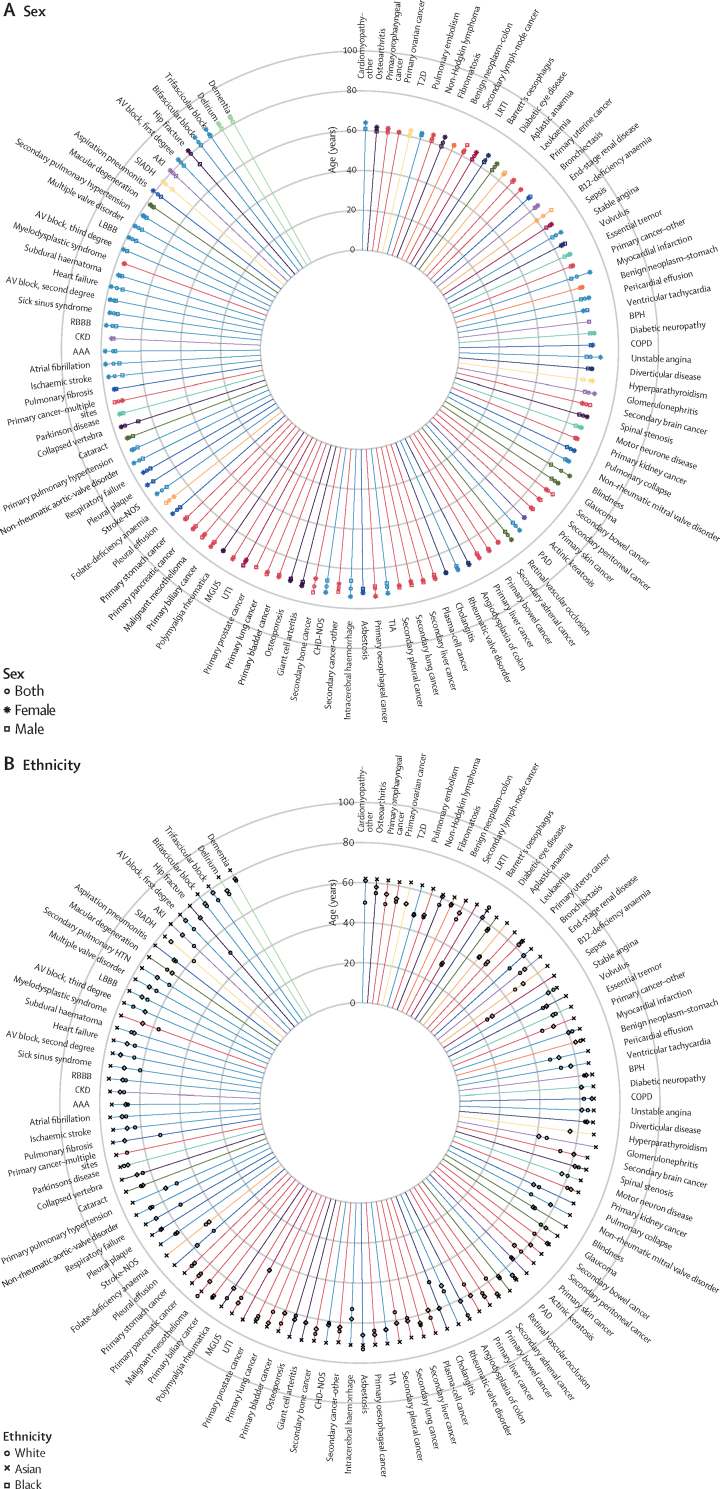
Median age at first record for diseases with median age of diagnosis after 60 years, stratified by sex (A) and ethnicity (B) For the key for the disease categories refer to figure 2. AAA=abdominal aortic aneurysm. AKI=acute kidney injury. AV=atrioventricular. BPH=benign prostatic hyperplasia. CHD=coronary heart disease. CKD=chronic kidney disease. COPD=chronic obstructive pulmonary disease. ID=infectious disease. LBBB=left bundle branch block. LRTI=lower respiratory-tract infection. MGUS= Monoclonal gammopathy of undetermined significance. NOS=not otherwise specified. PAD=peripheral arterial disease. RBBB=right bundle branch block. SIADH=syndrome of inappropriate antidiuretic hormone secretion. T2D=type 2 diabetes. TIA=transient ischaemic attack. UTI=urinary tract infection.

We examined the differences in median age of diagnosis of the health conditions in the study by ethnicity and sex (Figure 2, Figure 3, Figure 4 and appendix pp 27–37). Our ethnicity subanalysis found that white individuals had later median age at first record than black or south-Asian individuals for 258 (84%) of 308 conditions. Conditions with large differences in median age of diagnosis between ethnicities included sepsis (26 years [IQR 0–54] for black individuals *vs* 29 years [0–63] for south-Asian individuals *vs* 66 years [33–80] for white individuals), lower respiratory tract infection (30 years [3–51] *vs* 31 years [2–62] *vs* 65 years [33–80]), hip fracture (49 years [30–78] *vs* 72 years [47–81] *vs* 80 years [69–87]), encephalitis (18 years [6–43] *vs* 23 years [7–48] *vs* 48 years [23–69]), immunodeficiency (32 years [10–43] *vs* 11 years [2–41] *vs* 39 years [8–65]), urinary tract infection (45 years [28–71] *vs* 45 years [26–70] *vs* 73 years [47–84]), seborrheic dermatitis (11 years [2–33] *vs* 19 years [2–34] *vs* 37 years [15–58]), and sleep apnoea (29 years (4–45] *vs* 38 years [7–51] *vs* 49 years [36–60]).

In our subanalysis of differences between sexes, we found that women were diagnosed earlier than men with tubulointerstitial nephritis (median age 30 years [IQR 22–48] for women *vs* 53 years [35–68] for men), iron-deficiency anaemia (45 years [31–67] *vs* 66 years [44–77]), anaemia (other or unspecified; 48 years [32–74] *vs* 69 years [53–79]), chronic cystitis (53 years [36–67] *vs* 69 years [59–77]), urinary incontinence (51 years [40–66] *vs* 66 years [41–78]), secondary thrombocytopenia (48 years [30–69] *vs* 62 years [43–74]), fungal infection (50 years [26–75] *vs* 64 years [42–77]), and obstructive or reflux uropathy (43 years [20–67] *vs* 54 years [21–72]).

Men or boys were diagnosed at younger ages than women or girls with wrist fracture (median age 54 years [15–69] for women or girls *vs* 15 years [11–32] for men or boys), anorectal prolapse (64 years [46–77] *vs* 47 years [6–65]), gout (69 years [58–78] *vs* 56 years [45–67]), hypertrophic cardiomyopathy (65 years [51–76] *vs* 53 years [40–64]), spondylolisthesis (63 years [49–74] *vs* 51 years [37–66]), asthma (25 years [9–45] *vs* 13 years [5–37]), and urticaria (31 years [12–49] *vs* 19 years [6–43]).

We compared the study design and characteristics of our study with those of the GBD 2017 study^1^ and of Barnett and colleagues' study^2^ (appendix p 38). Prevalence values for 112 out of the 308 conditions in our study were previously reported by either one or the other study.^1^, ^2^ We compared the prevalence estimates between the three studies for the 112 overlapping conditions and reported the prevalence estimates for non-overlapping conditions by disease category (appendix pp 39–41 and 42–55).

**Figure 5:**
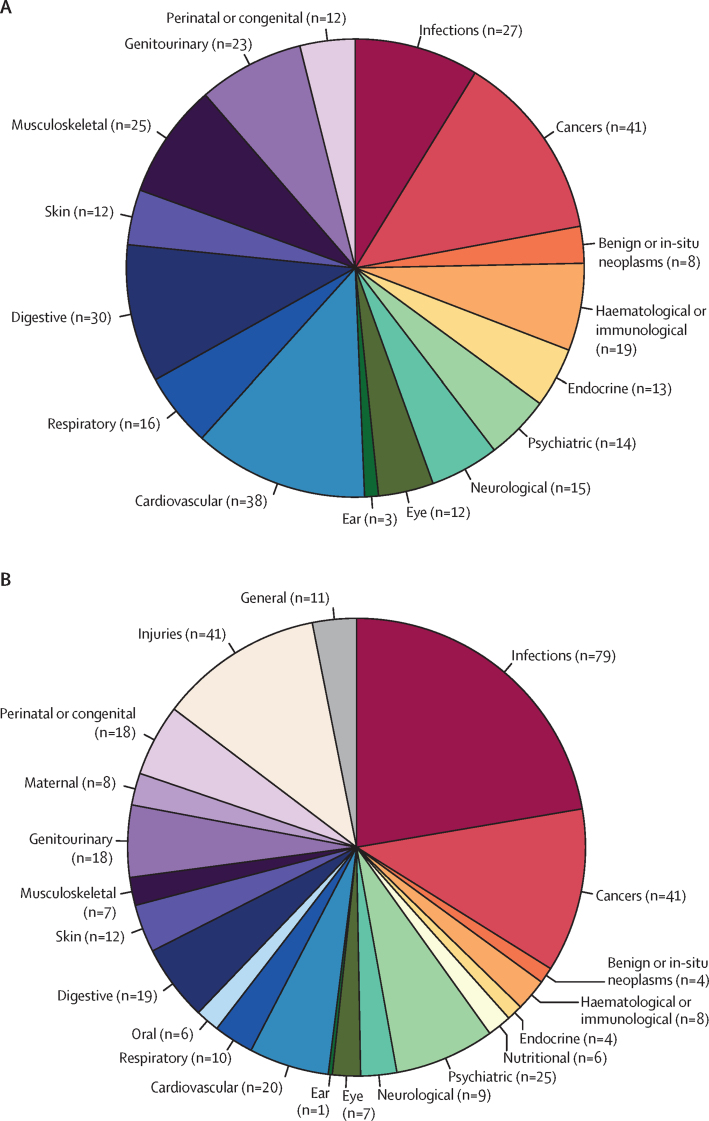
Number of diseases with reported case definitions and prevalences in the UK for each disease category from this study (A) or the Global Burden of Disease 2017 study^1^ (B)

Our study included more long-term conditions than the GBD study, which concentrated on infections, injuries, maternal conditions, and oral diseases (figure 5, appendix pp 42–55). We also reported on a wider range of neoplasms, including secondary malignancies and benign neoplasms, and mental health conditions such as personality disorder and obsessive-compulsive disorder, whereas the GBD study presented results for subtypes of substance use disorders, leukaemia, and liver cancer. 11 of the conditions reported by Barnett and colleagues^2^ were not directly comparable with the disease phenotypes reported here (appendix pp 42–55).

Prevalence estimates for most overlapping conditions were similar between this study and the GBD and Barnett and colleagues' studies (appendix pp 39–41), with the absolute difference in prevalence estimates between studies smaller than 2·5 percentage points for 71 of the 104 conditions included in both the GBD^1^ and this study, and for 19 of 29 conditions included in both this and the Barnett and colleagues' studies.^2^ The widest differences in prevalence estimates between our study and the GBD or Barnett and colleagues' studies^1^, ^2^ were for dermatitis (25·44% in our study *vs* not applicable (NA) in Barnett and colleagues' study^2^ *vs* 6·82% in the GBD study^1^), migraine (6·97% *vs* 0·60%^2^ *vs* 20·65%^1^), depression (17·44% *vs* 8·20%^2^ *vs* 4·36%^1^), deafness (8·86% *vs* 3·40%^2^ *vs* 19·12%^1^), anxiety (12·96% *vs* 3·20%^2^ *vs* 4·43%^1^), tuberculosis (0·68% *vs* NA^2^ *vs* 9·02%^1^), abdominal hernia (7·57% *vs* NA^2^ *vs* 0·38%^1^), asthma (14·99% *vs* 6·00%^2^ *vs* 8·37%^1^), and blindness (1·20% *vs* 0·50%^2^ *vs* 7·05%^1^).

## Discussion

We introduced the first chronological map of human health, with cumulative-incidence estimates, period-prevalence estimates, and median age at first record for 308 conditions from a single, large, clinically representative study population, stratified by age, sex, and ethnicity.

Although this chronological map reflects the burden of health conditions in England, it is likely to be relevant to other high-income countries with similar age and sex profiles. The findings complement the GBD reports, which have a wide geographical remit and hence encompass low-income, middle-income, and high-income countries. Prevalence estimates for some long-term conditions common in the NHS—such as hypertension, dyslipidaemia, irritable bowel syndrome, and thyroid disorders— were not included in GBD 2017.^1^ Our coverage of conditions was both wider (spanning both primary and secondary care) and more granular than in the seminal Scottish primary-care study by Barnett and colleagues.^2^ We classified cancers by major organ system, and subcategorised coronary heart disease into stable angina, myocardial infarction, unstable angina, and coronary heart disease not otherwise specified.

Frequency estimates for health conditions in this study convey the actual clinical experience of individuals in the NHS as documented in their EHRs. Prevalence estimates in the established medical literature vary widely and depend on multiple factors, including case definition, methodology, study sample, and time of measurement. Many disease-prevalence estimates are based on sparse sample sets from dated clinical studies, or surveys not representative of the general population. GBD 2017^1^ collated disease-distribution estimates from various sources including published literature, with renewed analysis of publicly available data and estimated prevalence with statistical models, whereas Barnett and colleagues^2^ reported point prevalence values based on EHRs from Scottish general-practice data in 2007. The period-prevalence estimates in this study are expected to be higher than the point-prevalence measures reported in the GBD or Barnett and colleagues' studies.^1^, ^2^ This is because we included all individuals who had ever been recorded with a condition, not just individuals presumed to have the condition at the time of analysis. Our period-prevalence calculation included people who had died during the study period; hence, conditions more common in those who died could have higher estimates in this report than in previous reports.^1^, ^2^ Another potential reason for higher prevalence estimates in this report than in Barnett and colleagues' study^2^ is the inclusion of secondary-care data in our case definitions. Comparison between these three large studies illustrates the similarities and differences in estimates using different methodologies, and allows the reader to select the method most relevant for their objectives. We reviewed disease frequency estimates from other published studies for conditions with the largest disparities between this study and the GBD or Barnett studies in the appendix (p 1).

Our age-stratified analysis enabled us to chronicle the passage of health conditions across the lifecourse. Hospital-admitted infections affected individuals at the extremes of life (<10 years and ≥80 years). Atopic conditions were common in childhood (<10 years). Mental health disorders were most prevalent from early adulthood (≥20 years). Menstrual disorders and migraine afflicted women of childbearing age (20–49 years). Metabolic conditions, particularly dyslipidaemias and obesity, together with hypertension, increased in prevalence from middle age (≥40 years). Cardiovascular diseases emerged later in life (≥60 years), following the surge in metabolic conditions in middle-age. Degenerative conditions involving the sense organs, musculoskeletal, genitourinary, and neurological systems were prominent in older individuals (≥80 years).

This is the first study to systematically document the age of diagnosis for hundreds of health conditions contemporaneously. Age of first recorded diagnosis encapsulates information regarding the age of onset and diagnosis, consultation patterns, and diagnostic and recording practices. It might also reveal different subtypes of disease according to sex and ethnicity.

The median age at first record was later in white individuals than in other ethnicities for 258 (84%) of the 308 conditions. Similar results have previously been reported for cancers.^15^ We found no significant differences between ethnicities in age-specific prevalence for acute infections, for which discrepancies in the age at first record between ethnicities were wide. Therefore, the later age at diagnosis for acute infections, and potentially other conditions, could be attributed to the higher proportion of older people in the white population than in other ethnicities. Other reasons for the younger ages at diagnosis of ethnic minorities could be a combination of genetic predisposition, socioeconomic status, or culturally determined health beliefs and practices.

Different pathological pathways might also be responsible for disorders affecting different ethnicities at different ages. Black individuals were diagnosed earlier with sleep apnoea than white individuals. This finding was consistent with our prevalence estimates, which identified a higher prevalence among black boys (aged <20 years) and white men older than 30 years. Sleep apnoea is usually associated with adenotonsillar hypertrophy in children,^16^ whereas in adults the main contributing factors are obesity, male sex, and age.^17^

Distinct disease mechanisms could also give rise to disparities in age of diagnosis between the sexes. Up to the age of 40 years, wrist fractures were more common in men and boys, but after this age, the incidence was higher in women. This difference could be attributed to higher-risk physical activities in young men or boys than in women or girls, and to decreased bone mineral density in older women (≥40 years). Male individuals were diagnosed with asthma at a younger age than female individuals, with earlier age at first record and higher cumulative incidence in boys younger than 10 years, whereas more women and girls were diagnosed with asthma after this age. This pattern of early-onset asthma in men or boys but late-onset in women or girls has been reported elsewhere.^18^, ^19^ Asthma is a heterogeneous disease, and early-onset asthma has been related to atopy and has substantial genetic susceptibility, whereas late-onset asthma tends to be non-allergic and induced by environmental and hormonal triggers.^18^, ^19^ Tubulointerstitial nephritis was diagnosed earlier in women than in men. An Australian study found an increase in acute interstitial nephritis in young women, which they attributed to immune-mediated conditions or analgesic nephropathy.^20^

Potential beneficiaries from this study include individual patients, patient groups, medical charities, practising clinicians (in primary and secondary care), health-care providers, public health organisations, policy makers, and medical researchers both in academia and industry, including those involved in drug development and evaluation.

Knowing the age-specific and sex-specific incidence and prevalence could help patients gain perspective into their conditions. Patient organisations can use these data for awareness campaigns and to support fundraising.

Our chronological map can guide clinicians assessing individual patients on the likelihood of possible diagnoses on the basis of their frequency distribution in the general population at different ages. It could also be the first step towards the creation of decision-support tools from EHRs using artificial intelligence.^21^

Age-specific incidence data on a wide range of preventable health conditions such as those presented in this Article are essential to realise the ambitions of the NHS Five Year Forward View^22^ and the Life Sciences Industrial Strategy,^23^ which have prioritised disease prevention and the development of new technologies to achieve this goal.

Commissioners of clinical services can use the findings from this study to inform budget allocation. The high prevalence of mental health, metabolic syndrome, musculoskeletal, and gynaecological conditions identified in this study highlights health-care delivery needs for these conditions. The incidence and prevalence of dementia will rise as the population ages. This will require not only effective drugs to prevent the onset of this condition, but also adequate social services to maintain the quality of life for affected individuals for as long as possible.

Our analysis lends support to calls for workforce expansion in key specialties.^24^ Adequate staffing is urgently needed to treat highly prevalent conditions at different stages of the lifecourse, such as mental health and gynaecological disorders from young adulthood to middle age, and musculoskeletal, neurodegenerative, and eye conditions in later life.

High degrees of disparity between research funding and disease burden have been shown in mental health, musculoskeletal, and cardiovascular conditions.^25^ Our findings reinforce the need for increased research investment into these conditions.

Delineating unmet health-care needs is crucial when planning and prioritising the initiation of new drug-development programmes. Understanding when specific disease endpoints are most likely to occur, and in which individuals, is essential in designing and planning clinical trials.

By providing the case definitions for hundreds of conditions and their median age at diagnosis, we are laying the foundation for future studies into multimorbidity and ageing-related diseases using EHRs. The need for this research has been highlighted in a 2018 report published by the Academy of Medical Sciences.^26^

The phenotyping algorithms in our platform can also be applied to EHRs linked to research-based cohort studies to provide disease-phenotype enrichment to support large-scale genetic-association studies.^27^, ^28^, ^29^ This integration of EHRs with genetic and other biomedical data enables a systems approach to the pathophysiology of disease. For example, phenome-wide association studies based on hospital EHRs are helping to identify diseases with common biological mechanisms.^30^ Collectively, these methods could unlock new opportunities for drug target discovery and repositioning.^31^

The main limitation in this study is its dependence on the accuracy of data recording. Although general practitioners directly enter codes into patients' EHRs during primary-care consultations, in secondary care, records are primarily paper-based and trained coders extract information from handwritten notes to allocate diagnoses and procedural codes for a hospital episode, during which process vital information could be misinterpreted and incorrectly reported. We expect the accuracy of secondary-care EHRs to improve with widespread adoption by clinicians of computerised hospital medical records.

Conditions might be under-represented in EHRs compared with surveys, as patients with mild to moderate symptoms might not present to health-care services. However, surveys are susceptible to non-response, response, selection, and volunteer biases, so the results might not be generalisable to the wider population.^32^ Asymptomatic cases can also lead to underestimates in conditions in which diagnosis requires clinical examination or investigations. Although clinical studies might detect asymptomatic cases, they are seldom representative of the general population.

A time-lag might occur between disease onset and the age of first record because of delays in clinical manifestation, presentation to the doctor, and documentation of the condition in the patients' records. Age at first diagnosis, therefore, might not reflect the actual age of onset, especially for diseases with a long subclinical phase.

The NHS Health Checks programme^33^ began in 2009 with the aim of reducing cardiovascular-disease risks and events. This has led to increased lipid profiling, blood pressure and BMI measurements in patients aged 40–74 years. Although this might have biased our estimation of incidence, prevalence, and age of first recorded diagnosis of dyslipidaemia towards middle-aged patients, it nevertheless allowed us to capture all relevant clinical measurements in a large population-based study, as opposed to relying on surveys or statistical estimations.

NHS England offers a range of other screening tests to different sections of the population, depending on their risk of developing specific conditions. These programmes aim to detect early signs of disease in asymptomatic individuals. Neonates are screened for rare metabolic conditions, including cystic fibrosis and sickle-cell disease. Pregnant women are screened for fetal anomalies, HIV, syphilis, hepatitis B, sickle-cell disease, and thalassaemia. Patients with diabetes are screened for eye complications. Cervical screening is offered to women aged 25–64 years and breast screening is offered to women aged 50–70 years. Bowel-cancer screening is offered to individuals aged 55 years in some parts of England and 60–74 years throughout England, and screening for abdominal aortic aneurysm is offered to men aged 65 years. The eligibility criteria for screening, together with differing response rates within the invited population might bias the generalisability of prevalence estimates based on EHRs. Nevertheless, these screening programmes allow more cases to be identified from EHRs than other study samples, which would not be devoid of biases in any case.

We have identified anomalies in the records due to inaccurate coding for rare conditions and disorders with asymptomatic or carrier states. Autosomal recessive disorders such as thalassaemia and cystic fibrosis had median ages at first record of 29 years and 31 years, later than would have been anticipated. These conditions had a bimodal distribution of age at first record, with a first peak in early childhood and the second peak at child-bearing age (appendix p 61). One explanation for these results could be that patients considering parenthood were erroneously coded as having these conditions after genetic screening tests revealed that they were heterozygous carriers. Another explanation is that mothers of neonates with these conditions were coded in lieu of their affected children who had not yet been registered with a general practice. Researchers using EHR data for these conditions should employ quality-control measures before analysis.

Caution needs to be exercised when interpreting the data for HIV, chronic hepatitis, and other sexually transmitted infections. In the UK, most consultations involving sexually transmitted infections are diagnosed and treated at sexual health service centres.^34^ The records from these services are not linked to primary or secondary care for reasons of confidentiality. Therefore, these conditions are under-reported in the CPRD linked dataset.

As the population ages and multimorbidity becomes more prevalent, clinicians, health-care planners, policy makers, and researchers need to know which sections of the population are vulnerable to which health conditions at which ages to prevent, detect, and treat these conditions effectively. We have generated a compendium of health conditions consisting of a comprehensive reference of case-definition algorithms and frequency-distribution patterns, together with a chronological map of human health conditions over the lifecourse to address this need.

## Data sharing

Algorithms and codelists for all 308 conditions included in our study are available on the CALIBER Portal. Our phenotyping algorithms and codelists are publicly available for readers to adopt and adapt for their own research, and can be downloaded in a machine-readable CSV format from a github data repository.
