# Supplementary material for: A chronological map of 308 physical and mental health conditions from 4 million individuals in the English National Health Service
Source: Lancet Digit Health. 2019 May 20;1(2):e63–77. doi: 10.1016/S2589-7500(19)30012-3 (PMC6798263; doi:10.1016/S2589-7500(19)30012-3)
Supplement: Supplementary appendix [file mmc1.pdf]

THE LANCET
Digital Health

Supplementary appendix

This appendix formed part of the original submission and has been peer reviewed. We post it as supplied by the authors.

Supplement to: Kuan V, Denaxas S, Gonzalez-Izquierdo A, et al. A chronological map of 308 physical and mental health conditions from 4 million individuals in the National Health Service. *Lancet Digital Health* 2019; published online May 20. http://dx.doi.org/10.1016/S2589-7500(19)30012-3.

Supplementary File.

Comparison of disease frequency estimates from previous reports for specific conditions
Different studies may describe disease frequency using different measures such as incidence, cumulative incidence, lifetime prevalence, or lifetime risk, making direct comparisons difficult. Comparison of disease distribution between studies is further complicated by variation in case definitions. For example, the term "dermatitis" may include any or all of atopic eczema, contact dermatitis, seborrheic dermatitis, or any unspecified skin rash.

Our prevalence estimate for dermatitis (25.43%) was much higher than the GBD[1] estimate (6.82%), and the prevalence for "psoriasis or eczema" (0.7%) from the Barnett[2] study. The lifetime prevalence of atopic eczema from a 1994 survey of 1,077 English children aged 3 to 11 years is often cited at 20%[3], and a 2003 Swedish survey of 8,469 individuals aged 20-59y reported the prevalence of eczema in adults at 14.6%[4]. Our estimates of 33% in children aged 1-9+years and 25.43% overall reflects the increase in prevalence of dermatitis over the years, as well as the inclusion of contact and unspecified dermatitis in our case definition.

A 2004 US survey of 162,705 individuals above the age of 12 found that 17.6% of females and 5.6% of males had current migraine[5]. We estimated the period prevalence of migraine to be 10.08% in females, and 3.85% in males. Our overall period prevalence of 6.97% is higher than the Barnett[2] estimate of 0.6%, but lower than the GBD[1] estimate of 20.65%. Migraneurs with mild to moderate symptoms may elect to self-treat and not consult their GPs, resulting in an underestimate of the prevalence from EHRs compared to surveys.

A survey in Great Britain conducted by Singleton et al in 2000 on 8,580 adults aged 16 to 74 years estimated the prevalence of depression to be 11%[6], while the QOF prevalence was 7.3% for 2014-2015[7]. These were higher than the GBD[1] prevalence of 4.36%, and similar to the Barnett[2] estimate of 8.20%. Our period prevalence of 17.44% included past as well as present cases, and was therefore higher than these point estimates. The Singleton prevalence for anxiety of 9% was higher than those for GBD[1] (4.43%) and Barnett[2] (3.2%), but was, as expected, lower than our estimate of 12.96%.

Action on Hearing Loss estimates the prevalence for hearing loss to be 17% in the UK[8], based on a study by Davis (1995)[9]. This is lower than the GBD 2017[1] estimate of 19.12% but higher than our (8.86%) and the Barnett[2] (3.4%) estimates. Many patients with hearing loss do not present to their GPs.

Public Health England (PHE) publishes the number of tuberculosis cases per annum, but not the prevalence. The case rate for 2015 was 1.05/10000[10]. This was consistent with our five-year cumulative incidence of TB of 5/10,000. Therefore, our period prevalence of 0.68% appears more consistent with PHE's figures than the GBD[1] estimate of 9.02%.

The Respiratory Health of the Nation project in the UK based on The Health Improvement Network (THIN) primary care EHRs estimated the lifetime prevalence of asthma from 12,600,000 patient records to be 12.6% in 2012[11]. This was higher than the Barnett[2] point estimate of 6% and the GBD[1] prevalence of 8.37% but lower than our period prevalence of 14.99%, which also included hospital admissions.

The 20-year cumulative incidence of inguinal hernia from American hospital records for 13,452 people from 1971-1993 was calculated at 13.9% for men and 2.1% for women[12]. The lifetime risk from birth of inguinal hernias for a lifespan of 85 years from hospital admission records in 1976-1986 in Oxford, UK, was estimated to be 27.2% for males and 2.6% for females[13]. Our period prevalence estimates (11.75% for males, 3.39% for females, 7.57% for both sexes) included all abdominal wall hernias as did the GBD[1] estimate (0.38%).

Supplementary Table S1. Medical Conditions with the abbreviated terms used in this article and their disease categories

**Condition**	**Abbreviated Term**	**Category**
Benign Neoplasm – Brain	Benign Neo – Brain	Benign Neoplasm/CIN
Benign Neoplasm – Colon	Benign Neo – Colon	Benign Neoplasm/CIN
Benign Neoplasm – Ovary	Benign Neo – Ovary	Benign Neoplasm/CIN
Benign Neoplasm – Stomach	Benign Neo – Stomach	Benign Neoplasm/CIN
Benign Neoplasm – Uterus	Benign Neo – Uterus	Benign Neoplasm/CIN
Cervical Intra-epithelial Neoplasia	CIN	Benign Neoplasm/CIN
Haemangioma	Haemangioma	Benign Neoplasm/CIN
Leiomyoma	Leiomyoma	Benign Neoplasm/CIN
Hodgkins Lymphoma	Hodgkins Lymphoma	Cancers
Leukaemia	Leukaemia	Cancers
Monoclonal Gammopathy of Undetermined Significance	MGUS	Cancers
Myelodysplastic Syndrome	Myelodysplastic Syndrome	Cancers
Non Hodgkins Lymphoma	Non Hodgkins Lymphoma	Cancers
Plasma Cell Malignancy	Plasma Cell Ca	Cancers
Polycythaemia vera	Polycythaemia vera	Cancers
Primary Malignancy – Biliary	Pri Ca – Biliary	Cancers
Primary Malignancy – Bladder	Pri Ca – Bladder	Cancers
Primary Malignancy – Bone	Pri Ca – Bone	Cancers
Primary Malignancy – Bowel	Pri Ca – Bowel	Cancers
Primary Malignancy – Brain	Pri Ca – Brain	Cancers
Primary Malignancy – Breast	Pri Ca – Breast	Cancers
Primary Malignancy – Cervix	Pri Ca – Cervix	Cancers
Primary Malignancy – Kidney	Pri Ca – Kidney	Cancers
Primary Malignancy – Liver	Pri Ca – Liver	Cancers
Primary Malignancy – Lung	Pri Ca – Lung	Cancers
Primary Malignancy – Melanoma	Pri Ca – Melanoma	Cancers
Primary Malignancy – Mesothelioma	Pri Ca – Mesothelioma	Cancers
Primary Malignancy – Multiple Sites	Pri Ca – Multiple Sites	Cancers
Primary Malignancy – Oesophageal	Pri Ca – Oesophageal	Cancers
Primary Malignancy – Oropharyngeal	Pri Ca – Oropharyngeal	Cancers
Primary Malignancy – other	Pri Ca – other	Cancers
Primary Malignancy – Ovary	Pri Ca – Ovary	Cancers
Primary Malignancy – Pancreas	Pri Ca – Pancreas	Cancers
Primary Malignancy – Prostate	Pri Ca – Prostate	Cancers
Primary Malignancy – Skin	Pri Ca – Skin	Cancers
Primary Malignancy – Stomach	Pri Ca – Stomach	Cancers
Primary Malignancy – Testis	Pri Ca – Testis	Cancers
Primary Malignancy – Thyroid	Pri Ca – Thyroid	Cancers
Primary Malignancy – Uterus	Pri Ca – Uterus	Cancers
Secondary Malignancy – Adrenal	Sec Ca – Adrenal	Cancers
Secondary Malignancy – Bone	Sec Ca – Bone	Cancers
Secondary Malignancy – Bowel	Sec Ca – Bowel	Cancers
Secondary Malignancy – Brain	Sec Ca – Brain	Cancers
Secondary Malignancy – Liver	Sec Ca – Liver	Cancers
Secondary Malignancy – Lung	Sec Ca – Lung	Cancers
Secondary Malignancy – Lymph Nodes	Sec Ca – Lymph Nodes	Cancers
Secondary Malignancy – other	Sec Ca – other	Cancers
Secondary Malignancy – Peritoneum	Sec Ca – Peritoneum	Cancers
Secondary Malignancy – Pleura	Sec Ca – Pleura	Cancers
Abdominal Aortic Aneurysm	AAA	Diseases of the Circulatory System
Atrial Fibrillation	Atrial Fibrillation	Diseases of the Circulatory System
Atrioventricular Block, first degree	AV Block, first degree	Diseases of the Circulatory System
Atrioventricular Block, second degree	AV Block, second degree	Diseases of the Circulatory System
Atrioventricular Block, third degree	AV Block, third degree	Diseases of the Circulatory System
Bifascicular Block	Bifascicular Block	Diseases of the Circulatory System
Cardiomyopathy – other	Cardiomyopathy – other	Diseases of the Circulatory System
Coronary Heart Disease (not otherwise specified)	CHD – nos	Diseases of the Circulatory System
Dilated cardiomyopathy	Dilated cardiomyopathy	Diseases of the Circulatory System
Heart Failure	Heart Failure	Diseases of the Circulatory System
Hypertension	Hypertension	Diseases of the Circulatory System
Hypertrophic cardiomyopathy	HOCM	Diseases of the Circulatory System
Intracerebral Haemorrhage	Intracerebral Haemorrhage	Diseases of the Circulatory System
Ischaemic Stroke	Ischaemic Stroke	Diseases of the Circulatory System
Left Bundle Branch Block	LBBB	Diseases of the Circulatory System
Multiple valve disorder	Multiple valve disorder	Diseases of the Circulatory System

Myocardial Infarction	Myocardial Infarction	Diseases of the Circulatory System
Non-rheumatic Aortic valve disorder	Non-rheum Aortic valve dz	Diseases of the Circulatory System
Non-rheumatic Mitral valve disorder	Non-rheum Mitral valve dz	Diseases of the Circulatory System
Pericardial Effusion	Pericardial Effusion	Diseases of the Circulatory System
Peripheral Arterial Disease	PAD	Diseases of the Circulatory System
Primary Pulmonary Hypertension	Pri Pulmonary HTN	Diseases of the Circulatory System
Pulmonary Embolism	Pulmonary Embolism	Diseases of the Circulatory System
Raynauds Disease	Raynauds Disease	Diseases of the Circulatory System
Rheumatic Valve Disorder	Rheum Valve dz	Diseases of the Circulatory System
Right Bundle Branch Block	RBBB	Diseases of the Circulatory System
Secondary Pulmonary Hypertension	Sec Pulmonary HTN	Diseases of the Circulatory System
Sick Sinus Syndrome	Sick Sinus Syndrome	Diseases of the Circulatory System
Stable Angina	Stable Angina	Diseases of the Circulatory System
Stroke – not otherwise specified	Stroke – nos	Diseases of the Circulatory System
Subarachnoid Haemorrhage	Subarach Haemorrhage	Diseases of the Circulatory System
Subdural haematoma	Subdural haematoma	Diseases of the Circulatory System
Supraventricular Tachycardia	SVT	Diseases of the Circulatory System
Transient Ischaemic Attack	TIA	Diseases of the Circulatory System
Trifascicular Block	Trifascicular Block	Diseases of the Circulatory System
Unstable Angina	Unstable Angina	Diseases of the Circulatory System
Venous thrombolism (Excl PE)	VTE (Excl PE)	Diseases of the Circulatory System
Ventricular Tachycardia	Ventricular Tachycardia	Diseases of the Circulatory System
Abdominal Hernia	Abdominal Hernia	Diseases of the Digestive System
Alcoholic Liver Disease	Alcoholic Liver Disease	Diseases of the Digestive System
Anal Fissure	Anal Fissure	Diseases of the Digestive System
Angiodysplasia of colon	Angiodysplasia of colon	Diseases of the Digestive System
Anorectal Fistula	Anorectal Fistula	Diseases of the Digestive System
Anorectal Prolapse	Anorectal Prolapse	Diseases of the Digestive System
Appendicitis	Appendicitis	Diseases of the Digestive System
Autoimmune liver disease	Autoimmune liver disease	Diseases of the Digestive System
Barrett's Oesophagus	Barrett's Oesophagus	Diseases of the Digestive System
Cholangitis	Cholangitis	Diseases of the Digestive System
Cholecystitis	Cholecystitis	Diseases of the Digestive System
Cholelithiasis	Cholelithiasis	Diseases of the Digestive System
Cirrhosis	Cirrhosis	Diseases of the Digestive System
Coeliac Disease	Coeliac Disease	Diseases of the Digestive System
Crohns Disease	Crohns Disease	Diseases of the Digestive System
Diaphragmatic Hernia	Diaphragmatic Hernia	Diseases of the Digestive System
Diverticular Disease	Diverticular Disease	Diseases of the Digestive System
Fatty Liver	Fatty Liver	Diseases of the Digestive System
Gastritis	Gastritis	Diseases of the Digestive System
Gastro-oesophageal Reflux Disease	GORD	Diseases of the Digestive System
Irritable Bowel Syndrome	IBS	Diseases of the Digestive System
Liver Failure	Liver Failure	Diseases of the Digestive System
Oesophageal Ulcer	Oesophageal Ulcer	Diseases of the Digestive System
Oesophageal Varices	Oesophageal Varices	Diseases of the Digestive System
Pancreatitis	Pancreatitis	Diseases of the Digestive System
Peptic Ulcer	Peptic Ulcer	Diseases of the Digestive System
Peritonitis	Peritonitis	Diseases of the Digestive System
Portal Hypertension	Portal Hypertension	Diseases of the Digestive System
Ulcerative Colitis	Ulcerative Colitis	Diseases of the Digestive System
Volvulus	Volvulus	Diseases of the Digestive System
Deafness	Deafness	Diseases of the Ear
Meniere's Disease	Meniere's Disease	Diseases of the Ear
Tinnitus	Tinnitus	Diseases of the Ear
Cystic Fibrosis	Cystic Fibrosis	Diseases of the Endocrine System
Diabetes Mellitus – other or not specified	DM – other or nos	Diseases of the Endocrine System
Hyperparathyroidism	Hyperparathyroidism	Diseases of the Endocrine System
Low HDL-C	Low HDL-C	Diseases of the Endocrine System
Obesity	Obesity	Diseases of the Endocrine System
Polycystic Ovarian Syndrome	PCOS	Diseases of the Endocrine System
Raised LDL-C	Raised LDL-C	Diseases of the Endocrine System
Raised Total Cholesterol	Raised Total Cholesterol	Diseases of the Endocrine System
Raised Triglycerides	Raised Triglycerides	Diseases of the Endocrine System
Syndrome of Inappropriate AntiDiuretic Hormone	SIADH	Diseases of the Endocrine System
Thyroid Disease	Thyroid Disease	Diseases of the Endocrine System
Type 1 Diabetes Mellitus	T1DM	Diseases of the Endocrine System
Type 2 Diabetes Mellitus	T2DM	Diseases of the Endocrine System
Anterior Uveitis	Anterior Uveitis	Diseases of the Eye
Blindness	Blindness	Diseases of the Eye

Cataract	Cataract	Diseases of the Eye
Diabetic Eye Disease	Diabetic Eye Disease	Diseases of the Eye
Glaucoma	Glaucoma	Diseases of the Eye
Keratitis	Keratitis	Diseases of the Eye
Macular Degeneration	Macular Degeneration	Diseases of the Eye
Posterior Uveitis	Posterior Uveitis	Diseases of the Eye
Ptosis	Ptosis	Diseases of the Eye
Retinal Detachment	Retinal Detachment	Diseases of the Eye
Retinal Vascular Occlusion	Retinal Vascular Occlusion	Diseases of the Eye
Scleritis	Scleritis	Diseases of the Eye
Acute Kidney Injury	AKI	Diseases of the Genitourinary system
Benign Prostatic Hyperplasia	BPH	Diseases of the Genitourinary system
Chronic Cystitis	Chronic Cystitis	Diseases of the Genitourinary system
Chronic Kidney Disease	CKD	Diseases of the Genitourinary system
Dysmenorrhoea	Dysmenorrhoea	Diseases of the Genitourinary system
End Stage Renal Disease	End Stage Renal Disease	Diseases of the Genitourinary system
Endometrial Hyperplasia	Endometrial Hyperplasia	Diseases of the Genitourinary system
Endometriosis	Endometriosis	Diseases of the Genitourinary system
Erectile Dysfunction	Erectile Dysfunction	Diseases of the Genitourinary system
Female Infertility	Female Infertility	Diseases of the Genitourinary system
Glomerulonephritis	Glomerulonephritis	Diseases of the Genitourinary system
Hydrocele	Hydrocele	Diseases of the Genitourinary system
Male infertility	Male infertility	Diseases of the Genitourinary system
Menorrhagia	Menorrhagia	Diseases of the Genitourinary system
Neuropahtic Bladder	Neuropahtic Bladder	Diseases of the Genitourinary system
Obstructive and reflux uropathy	Obstruct/reflux uropathy	Diseases of the Genitourinary system
Postcoital Bleeding	Postcoital Bleeding	Diseases of the Genitourinary system
Postmenopausal Bleeding	Postmenopausal Bleeding	Diseases of the Genitourinary system
Tubulo-interstitial Nephropathy	Tubulo-interstitial Nephro	Diseases of the Genitourinary system
Undescended Testis	Undescended Testis	Diseases of the Genitourinary system
Urinary Incontinence	Urinary Incontinence	Diseases of the Genitourinary system
Urolithiasis	Urolithiasis	Diseases of the Genitourinary system
Uterovaginal Prolapse	Uterovaginal Prolapse	Diseases of the Genitourinary system
Allergic/chronic Rhinitis	Allergic/chronic Rhinitis	Diseases of the Respiratory System
Asbestosis	Asbestosis	Diseases of the Respiratory System
Aspiration Pneumonitis	Aspiration Pneumonitis	Diseases of the Respiratory System
Asthma	Asthma	Diseases of the Respiratory System
Bronchiectasis	Bronchiectasis	Diseases of the Respiratory System
Chronic Obstructive Pulmonary Disease	COPD	Diseases of the Respiratory System
Chronic Sinusitis	Chronic Sinusitis	Diseases of the Respiratory System
Hypertrophic Nasal Turbinates	Hypertroph Nasal Turbs	Diseases of the Respiratory System
Nasal Polyps	Nasal Polyps	Diseases of the Respiratory System
Pleural Effusion	Pleural Effusion	Diseases of the Respiratory System
Pleural Plaque	Pleural Plaque	Diseases of the Respiratory System
Pneumothorax	Pneumothorax	Diseases of the Respiratory System
Pulmonary Collapse	Pulmonary Collapse	Diseases of the Respiratory System
Pulmonary Fibrosis	Pulmonary Fibrosis	Diseases of the Respiratory System
Respiratory Failure	Respiratory Failure	Diseases of the Respiratory System
Sleep apnoea	Sleep apnoea	Diseases of the Respiratory System
Agranulocytosis	Agranulocytosis	Haematological/Immunological conditions
Anaemia – other	Anaemia – other	Haematological/Immunological conditions
Aplastic Anaemia	Aplastic Anaemia	Haematological/Immunological conditions
Folate Deficiency Anaemia	Folate Def Anaemia	Haematological/Immunological conditions
Hypersplenism	Hypersplenism	Haematological/Immunological conditions
Hyposplenism	Hyposplenism	Haematological/Immunological conditions
Immunodeficiency	Immunodeficiency	Haematological/Immunological conditions
Iron Deficiency Anaemia	Iron Def Anaemia	Haematological/Immunological conditions
Other haemolytic anaemia	Other haemolytic anaemia	Haematological/Immunological conditions
Primary thrombocytopaenia	Pri thrombocytopaenia	Haematological/Immunological conditions
Sarcoidosis	Sarcoidosis	Haematological/Immunological conditions
Secondary Polycythaemia	Sec Polycythaemia	Haematological/Immunological conditions
Secondary Thrombocytopaenia	Sec Thrombocytopaenia	Haematological/Immunological conditions
Sickle Cell Disease	Sickle Cell Disease	Haematological/Immunological conditions
Sickle Cell Trait	Sickle Cell Trait	Haematological/Immunological conditions
Thalassaemia	Thalassaemia	Haematological/Immunological conditions
Thalassaemia Trait	Thalassaemia Trait	Haematological/Immunological conditions
Thrombophilia	Thrombophilia	Haematological/Immunological conditions
Vitamin B12 deficiency anaemia	B12 def anaemia	Haematological/Immunological conditions
Bacterial Infection	ID-Bacterial	Infectious Diseases
Chronic Viral Hepatitis	Chronic Hepatitis	Infectious Diseases
Encephalitis	Encephalitis	Infectious Diseases

Fungal Infection	ID-Fungal	Infectious Diseases
HIV	HIV	Infectious Diseases
Infection – Anorectal	ID–Anorectal	Infectious Diseases
Infection – Bone	ID–Bone	Infectious Diseases
Infection – Digestive System	ID–Digestive	Infectious Diseases
Infection – Ear/Upper Respiratory Tract	URTI	Infectious Diseases
Infection – Eye	ID–Eye	Infectious Diseases
Infection – Heart	ID–Heart	Infectious Diseases
Infection – Liver	ID–Liver	Infectious Diseases
Infection – Lower Respiratory Tract	LRTI	Infectious Diseases
Infection – Male Genitourinary	ID–Male GU	Infectious Diseases
Infection – Other Genitourianry	ID–Other GU	Infectious Diseases
Infection – Other nervous system	ID–Other nervous sys	Infectious Diseases
Infection – Other organisms	ID–Other organisms	Infectious Diseases
Infection – Other organs	ID–Other organs	Infectious Diseases
Infection – Skin	ID–Skin	Infectious Diseases
Meningitis	Meningitis	Infectious Diseases
Parasitic Infection	ID-Parasitic	Infectious Diseases
Pelvic Inflammatory Disease	PID	Infectious Diseases
Rheumatic Fever	Rheumatic Fever	Infectious Diseases
Septicaemia	Septicaemia	Infectious Diseases
Tuberculosis	Tuberculosis	Infectious Diseases
Urinary Tract Infection	UTI	Infectious Diseases
Viral Infection	ID-Viral	Infectious Diseases
Alcohol Misuse	Alcohol Misuse	Mental Health Disorders
Anxiety	Anxiety	Mental Health Disorders
Autism	Autism	Mental Health Disorders
Bipolar Affective Disorder	Bipolar Affective Disorder	Mental Health Disorders
Delirium	Delirium	Mental Health Disorders
Dementia	Dementia	Mental Health Disorders
Depression	Depression	Mental Health Disorders
Eating Disorders	Eating Disorders	Mental Health Disorders
Hyperkinetic Disorders	Hyperkinetic Disorders	Mental Health Disorders
Intellectual Disability	Intellectual Disability	Mental Health Disorders
Obsessive Compulsive Disorder	OCD	Mental Health Disorders
Personality Disorder	Personality Disorder	Mental Health Disorders
Schizophrenia	Schizophrenia	Mental Health Disorders
Substance Misuse	Substance Misuse	Mental Health Disorders
Ankylosing Spondylosis	Ankylosing Spondylosis	Musculoskeletal conditions
Carpal Tunnel Syndrome	Carpal Tunnel Syndrome	Musculoskeletal conditions
Collapsed Vertebra	Collapsed Vertebra	Musculoskeletal conditions
Enteropathic Arthropathy	Enteropathic Arthropathy	Musculoskeletal conditions
Enthesopathy and synovial disorder	Enthesopathy	Musculoskeletal conditions
Fibromatosis	Fibromatosis	Musculoskeletal conditions
Fracture – Hip	Fracture – Hip	Musculoskeletal conditions
Fracture – Wrist	Fracture – Wrist	Musculoskeletal conditions
Giant Cell Arteritis	Giant Cell Arteritis	Musculoskeletal conditions
Gout	Gout	Musculoskeletal conditions
Intervertebral Disc Disorder	Intervertebral Disc Disorder	Musculoskeletal conditions
Juvenile Arthritis	Juvenile Arthritis	Musculoskeletal conditions
Lupus Erythematosus	Lupus Erythematosus	Musculoskeletal conditions
Osteoarthritis	Osteoarthritis	Musculoskeletal conditions
Osteoporosis	Osteoporosis	Musculoskeletal conditions
Polymyalgia Rheumatica	Polymyalgia Rheumatica	Musculoskeletal conditions
Psoriatic Arthritis	Psoriatic Arthritis	Musculoskeletal conditions
Reactive Arthritis	Reactive Arthritis	Musculoskeletal conditions
Rheumatoid Arthritis	Rheumatoid Arthritis	Musculoskeletal conditions
Scleroderma	Scleroderma	Musculoskeletal conditions
Scoliosis	Scoliosis	Musculoskeletal conditions
Sjogren Syndrome	Sjogren Syndrome	Musculoskeletal conditions
Spinal Stenosis	Spinal Stenosis	Musculoskeletal conditions
Spondylolisthesis	Spondylolisthesis	Musculoskeletal conditions
Spondylosis	Spondylosis	Musculoskeletal conditions
Autonomic Neuropathy	Autonomic Neuropathy	Neurological conditions
Bell's Palsy	Bell's Palsy	Neurological conditions
Cerebral Palsy	Cerebral Palsy	Neurological conditions
Chronic Fatigue Syndrome	Chronic Fatigue Syndrome	Neurological conditions
Diabetic Neuropathy	Diabetic Neuropathy	Neurological conditions
Epilepsy	Epilepsy	Neurological conditions
Essential Tremor	Essential Tremor	Neurological conditions
Idiopathic Intracranial Hypertension	Idiopathic Intracranial HTN	Neurological conditions

Migraine	Migraine	Neurological conditions
Motor Neurone Disease	Motor Neurone Disease	Neurological conditions
Multiple Sclerosis	Multiple Sclerosis	Neurological conditions
Myasthenia Gravis	Myasthenia Gravis	Neurological conditions
Parkinson's Disease	Parkinson's Disease	Neurological conditions
Peripheral Neuropathy	Peripheral Neuropathy	Neurological conditions
Trigeminal Neuralgia	Trigeminal Neuralgia	Neurological conditions
Congenital Septal Defect	Congenital Septal Defect	Perinatal conditions
Down Syndrome	Down Syndrome	Perinatal conditions
High Birth Weight	High Birth Weight	Perinatal conditions
Intrauterine Hypoxia	Intrauterine Hypoxia	Perinatal conditions
Low Birth Weight	Low Birth Weight	Perinatal conditions
Neonatal Jaundice	Neonatal Jaundice	Perinatal conditions
Patent Ductus Arteriosus	Patent Ductus Arteriosus	Perinatal conditions
Post-term Delivery	Post-term Delivery	Perinatal conditions
Premature Delivery	Premature Delivery	Perinatal conditions
Respiratory Distress of the Newborn	Resp Distress Newborn	Perinatal conditions
Sepsis of the Newborn	Sepsis of the Newborn	Perinatal conditions
Spina Bifida	Spina Bifida	Perinatal conditions
Acne	Acne	Skin conditions
Actinic keratosis	Actinic keratosis	Skin conditions
Alopecia Areata	Alopecia Areata	Skin conditions
Dermatitis	Dermatitis	Skin conditions
Hidradenitis suppurativa	Hidradenitis	Skin conditions
Lichen Planus	Lichen Planus	Skin conditions
Pilonidal cyst/sinus	Pilonidal cyst/sinus	Skin conditions
Psoriasis	Psoriasis	Skin conditions
Rosacea	Rosacea	Skin conditions
Seborrheic Dermatitis	Seborrheic Dermatitis	Skin conditions
Urticaria	Urticaria	Skin conditions
Vitiligo	Vitiligo	Skin conditions

Supplementary Table S2. Ethnicity in CALIBER mapped to Five-level Ethnicity Category

CALIBER Category	CALIBER Definition	Five-level Ethnicity Category
1	White British	White
2	White Irish	White
3	White Other	White
4	White NOS	White
5	Mixed White and Black	Mixed
6	Mixed White and Asian	Mixed
7	Mixed Asian and Black	Mixed
8	Mixed Other	Mixed
9	Mixed NOS	Mixed
10	Indian	South Asian
11	Pakistani	South Asian
12	Bangladeshi	South Asian
13	Other Asian	South Asian
15	Black Caribbean	Black
16	Black African	Black
17	Black Other	Black
19	Chinese	Other
20	Other ethnic group	Other
21	Ethnic group not specified	Unknown

Supplementary Table S3. Ethnicity in HES mapped to Five-level Ethnicity Category

gen_ethnicity	Five-level Ethnicity Category
Bangladesi	South Asian
Bl_Afric	Black
Bl_Carib	Black
Bl_Other	Black
Chinese	Other
Indian	South Asian
Mixed	Mixed
Oth_Asian	South Asian
Other	Other
Pakistani	South Asian
Unknown	Other
White	White

Supplementary Table S4. Sex-standardised period prevalence per 10000 (95% confidence intervals) from 1 April 2010 to 31 Mar 2015 for 308 medical conditions , stratified by age on 1 April 2010.

Condition	Category	Age 0-9+ years	Age 10-19+ years	Age 20-29+ years	Age 30-39+ years	Age 40-49+ years	Age 50-59+ years	Age 60-69+ years	Age 70-79+ years	Age 80+ years
Benign Neoplasm – Brain	Benign Neo	--	3 (2, 3)	7 (6, 8)	13 (12, 14)	22 (21, 23)	35 (33, 36)	46 (44, 48)	65 (62, 68)	71 (68, 75)
Benign Neoplasm – Colon	Benign Neo	2 (2, 2)	7 (6, 8)	32 (31, 34)	75 (73, 78)	174 (170, 177)	388 (382, 394)	706 (697, 714)	879 (867, 891)	742 (729, 756)
Benign Neoplasm – Ovary	Benign Neo	4 (3, 5)	113 (108, 117)	319 (313, 326)	496 (487, 504)	678 (668, 688)	593 (582, 603)	421 (411, 430)	325 (315, 335)	243 (234, 252)
Benign Neoplasm – Stomach	Benign Neo	--	--	3 (2, 3)	9 (8, 10)	28 (27, 30)	69 (67, 72)	129 (126, 133)	188 (183, 194)	177 (171, 184)
Benign Neoplasm – Uterus	Benign Neo	--	--	19 (17, 20)	87 (84, 91)	249 (243, 255)	380 (372, 388)	396 (387, 405)	305 (296, 315)	157 (150, 164)
Cervical Intra-epithelial Neoplasia	Benign Neo	--	--	287 (280, 293)	519 (510, 528)	444 (436, 452)	315 (308, 323)	189 (183, 195)	89 (84, 94)	32 (28, 35)
Haemangioma	Benign Neo	149 (146, 153)	103 (100, 106)	75 (73, 78)	104 (101, 106)	130 (127, 133)	153 (150, 157)	161 (157, 165)	150 (145, 155)	130 (124, 135)
Leiomyoma	Benign Neo	--	--	42 (39, 44)	249 (243, 255)	770 (760, 781)	953 (940, 966)	699 (687, 711)	385 (375, 396)	217 (208, 225)
Hodgkins Lymphoma	Cancers	--	3 (2, 3)	5 (5, 6)	7 (6, 8)	9 (9, 10)	9 (9, 10)	12 (11, 13)	11 (9, 12)	7 (6, 8)
Leukaemia	Cancers	5 (5, 6)	6 (5, 7)	6 (5, 7)	7 (6, 7)	9 (9, 10)	18 (17, 20)	41 (39, 43)	74 (71, 78)	92 (88, 97)
Monoclonal Gammopathy of Undetermined Significance	Cancers	--	--	--	--	3 (2, 3)	8 (7, 9)	24 (22, 25)	53 (50, 56)	70 (66, 73)
Myelodysplastic Syndrome	Cancers	--	--	--	--	2 (2, 3)	5 (5, 6)	15 (13, 16)	43 (40, 45)	91 (87, 96)
Non Hodgkins Lymphoma	Cancers	--	3 (2, 3)	5 (5, 6)	10 (9, 10)	16 (15, 17)	31 (30, 33)	63 (61, 66)	95 (91, 99)	98 (94, 103)
Plasma Cell Malignancy	Cancers	--	--	--	--	3 (2, 3)	9 (8, 9)	23 (21, 24)	41 (38, 43)	48 (44, 51)
Polycythaemia vera	Cancers	--	--	--	4 (3, 4)	8 (8, 9)	16 (15, 17)	24 (23, 26)	31 (29, 33)	31 (29, 34)
Primary Malignancy – Biliary	Cancers	--	--	--	--	--	4 (3, 5)	9 (8, 10)	15 (13, 16)	19 (17, 21)
Primary Malignancy – Bladder	Cancers	--	--	--	2 (2, 2)	8 (7, 8)	25 (23, 26)	83 (80, 86)	193 (188, 199)	295 (286, 304)
Primary Malignancy – Bone	Cancers	--	--	2 (2, 2)	3 (2, 3)	3 (3, 4)	4 (3, 4)	6 (5, 7)	8 (7, 9)	9 (8, 11)
Primary Malignancy – Bowel	Cancers	--	--	2 (2, 2)	5 (4, 5)	18 (17, 19)	60 (58, 63)	156 (152, 160)	317 (310, 325)	437 (427, 447)
Primary Malignancy – Brain	Cancers	3 (2, 3)	4 (4, 5)	5 (5, 6)	6 (5, 7)	8 (8, 9)	12 (11, 13)	17 (15, 18)	20 (18, 21)	16 (14, 18)
Primary Malignancy – Breast	Cancers	--	--	3 (3, 4)	21 (20, 23)	100 (97, 102)	209 (205, 214)	350 (344, 355)	383 (376, 391)	366 (358, 374)
Primary Malignancy – Cervix	Cancers	--	--	7 (6, 8)	17 (15, 18)	23 (22, 25)	31 (28, 33)	36 (33, 39)	33 (30, 36)	33 (30, 36)
Primary Malignancy – Kidney	Cancers	--	--	--	--	7 (6, 7)	17 (16, 18)	39 (37, 41)	66 (63, 69)	67 (63, 71)
Primary Malignancy – Liver	Cancers	--	--	--	--	--	5 (5, 6)	10 (9, 11)	16 (15, 18)	15 (13, 16)
Primary Malignancy – Lung	Cancers	--	--	--	--	9 (8, 10)	38 (36, 40)	108 (105, 112)	187 (181, 192)	183 (176, 189)
Primary Malignancy – Melanoma	Cancers	--	--	9 (8, 10)	23 (21, 24)	42 (40, 44)	65 (62, 67)	105 (101, 108)	143 (139, 148)	166 (160, 172)
Primary Malignancy – Mesothelioma	Cancers	--	--	--	--	--	--	7 (6, 8)	15 (13, 17)	12 (10, 14)
Primary Malignancy – Multiple Sites	Cancers	--	--	--	--	--	--	3 (3, 4)	7 (6, 8)	8 (6, 9)
Primary Malignancy – Oesophageal	Cancers	--	--	--	--	3 (2, 3)	13 (12, 14)	30 (28, 32)	49 (46, 52)	57 (54, 61)
Primary Malignancy – Oropharyngeal	Cancers	--	--	--	3 (3, 4)	11 (10, 12)	27 (25, 28)	38 (36, 40)	46 (43, 48)	52 (48, 55)
Primary Malignancy – other	Cancers	3 (3, 4)	4 (3, 4)	4 (4, 5)	8 (7, 9)	16 (15, 17)	36 (34, 38)	68 (66, 71)	106 (102, 110)	131 (126, 136)
Primary Malignancy – Ovary	Cancers	--	--	--	7 (6, 8)	20 (18, 22)	41 (38, 43)	69 (65, 73)	88 (83, 93)	64 (59, 68)
Primary Malignancy – Pancreas	Cancers	--	--	--	--	3 (2, 3)	9 (8, 10)	22 (20, 23)	39 (36, 41)	44 (41, 47)
Primary Malignancy – Prostate	Cancers	--	--	--	--	12 (11, 14)	103 (99, 107)	432 (422, 441)	927 (910, 945)	1211 (1184,

										1238)
Primary Malignancy – Skin	Cancers	--	--	8 (7, 8)	32 (31, 34)	103 (100, 105)	249 (244, 254)	584 (576, 591)	1127 (1114, 1141)	1790 (1770, 1811)
Primary Malignancy – Stomach	Cancers	--	--	--	--	3 (2, 3)	9 (8, 10)	21 (20, 22)	46 (43, 49)	56 (53, 60)
Primary Malignancy – Testis	Cancers	--	--	13 (11, 14)	24 (22, 26)	29 (27, 31)	26 (24, 28)	19 (17, 20)	13 (11, 15)	--
Primary Malignancy – Thyroid	Cancers	--	--	3 (2, 3)	7 (6, 7)	8 (8, 9)	10 (9, 11)	11 (10, 12)	12 (10, 13)	9 (8, 11)
Primary Malignancy – Uterus	Cancers	--	--	--	--	12 (10, 13)	42 (40, 45)	100 (96, 105)	137 (131, 144)	107 (101, 113)
Secondary Malignancy – Adrenal	Cancers	--	--	--	--	--	6 (5, 7)	13 (12, 14)	17 (16, 19)	12 (10, 14)
Secondary Malignancy – Bone	Cancers	--	--	--	4 (3, 4)	13 (12, 14)	33 (31, 35)	77 (74, 80)	135 (131, 140)	155 (148, 161)
Secondary Malignancy – Bowel	Cancers	--	--	--	--	--	3 (3, 4)	8 (7, 9)	12 (10, 13)	8 (7, 10)
Secondary Malignancy – Brain	Cancers	--	--	--	2 (1, 2)	7 (6, 7)	15 (14, 16)	29 (27, 31)	34 (32, 37)	29 (26, 31)
Secondary Malignancy – Liver	Cancers	--	--	--	5 (4, 5)	16 (15, 17)	42 (41, 44)	92 (88, 95)	149 (145, 154)	144 (139, 150)
Secondary Malignancy – Lung	Cancers	--	--	--	5 (4, 5)	12 (11, 13)	29 (27, 30)	62 (60, 65)	104 (100, 108)	91 (86, 95)
Secondary Malignancy – Lymph Nodes	Cancers	--	--	5 (4, 5)	15 (14, 16)	46 (44, 48)	96 (93, 99)	158 (154, 162)	196 (190, 201)	134 (129, 140)
Secondary Malignancy – other	Cancers	--	--	--	2 (2, 3)	8 (7, 8)	18 (16, 19)	37 (35, 38)	65 (61, 68)	82 (78, 86)
Secondary Malignancy – Peritoneum	Cancers	--	--	--	2 (2, 3)	7 (6, 8)	19 (18, 20)	39 (37, 41)	53 (50, 56)	32 (30, 35)
Secondary Malignancy – Pleura	Cancers	--	--	--	--	3 (2, 3)	8 (7, 9)	17 (16, 18)	27 (25, 29)	23 (21, 25)
Abdominal Aortic Aneurysm	Cardiovascular	--	--	--	--	4 (3, 4)	13 (12, 14)	91 (88, 94)	241 (235, 248)	391 (380, 401)
Atrial Fibrillation	Cardiovascular	--	3 (2, 3)	11 (10, 12)	27 (26, 28)	73 (71, 75)	213 (209, 217)	643 (635, 651)	1701 (1684, 1717)	3229 (3202, 3256)
Atrioventricular Block, first degree	Cardiovascular	--	--	--	2 (2, 3)	5 (5, 6)	13 (12, 14)	36 (34, 38)	127 (122, 131)	333 (324, 342)
Atrioventricular Block, second degree	Cardiovascular	--	--	--	--	3 (2, 3)	6 (5, 7)	17 (16, 18)	51 (48, 54)	101 (96, 105)
Atrioventricular Block, third degree	Cardiovascular	--	--	--	--	3 (2, 3)	7 (7, 8)	23 (22, 25)	74 (71, 78)	179 (173, 186)
Bifascicular Block	Cardiovascular	--	--	--	--	--	--	3 (3, 4)	13 (11, 14)	38 (35, 41)
Cardiomyopathy – other	Cardiovascular	--	3 (3, 4)	5 (4, 5)	8 (7, 8)	16 (15, 17)	31 (30, 33)	59 (57, 61)	81 (77, 84)	75 (71, 79)
Coronary Heart Disease (not otherwise specified)	Cardiovascular	--	--	3 (2, 3)	8 (8, 9)	34 (32, 35)	94 (91, 97)	211 (207, 216)	386 (379, 394)	569 (558, 580)
Dilated cardiomyopathy	Cardiovascular	--	--	--	4 (4, 5)	11 (10, 11)	21 (19, 22)	35 (33, 36)	45 (42, 47)	29 (26, 31)
Heart Failure	Cardiovascular	5 (4, 6)	5 (4, 5)	7 (6, 8)	14 (13, 15)	45 (43, 46)	133 (130, 136)	362 (355, 368)	1022 (1010, 1035)	2318 (2296, 2341)
Hypertension	Cardiovascular	9 (8, 10)	20 (18, 21)	91 (89, 94)	370 (365, 375)	1180 (1171, 1189)	2655 (2640, 2670)	4551 (4530, 4573)	6476 (6444, 6508)	7375 (7335, 7415)
Hypertrophic cardiomyopathy	Cardiovascular	--	--	--	2 (2, 3)	4 (4, 5)	8 (7, 9)	11 (10, 12)	15 (13, 16)	14 (12, 15)
Intracerebral Haemorrhage	Cardiovascular	3 (2, 3)	3 (2, 3)	3 (3, 4)	5 (5, 6)	12 (11, 13)	25 (23, 26)	51 (49, 53)	109 (104, 113)	184 (177, 190)
Ischaemic Stroke	Cardiovascular	3 (2, 3)	2 (2, 3)	4 (4, 5)	12 (11, 12)	32 (30, 33)	85 (82, 88)	207 (202, 211)	501 (492, 510)	935 (921, 949)
Left Bundle Branch Block	Cardiovascular	--	--	--	3 (2, 3)	10 (9, 11)	29 (28, 31)	74 (72, 77)	212 (207, 218)	435 (425, 445)
Multiple valve disorder	Cardiovascular	--	--	3 (2, 3)	5 (4, 5)	11 (10, 12)	29 (27, 31)	78 (75, 81)	232 (226, 238)	420 (410, 429)
Myocardial Infarction	Cardiovascular	--	--	4 (3, 4)	18 (17, 19)	89 (86, 91)	271 (266, 275)	558 (550, 565)	1108 (1095, 1122)	1670 (1650, 1690)
Non-rheumatic Aortic valve disorder	Cardiovascular	5 (4, 5)	7 (6, 8)	8 (7, 9)	10 (9, 11)	19 (18, 20)	48 (46, 50)	141 (138, 145)	387 (379, 394)	697 (684, 709)
Non-rheumatic Mitral valve	Cardiovascular	3 (3, 4)	6 (5, 7)	11 (10, 12)	21 (20, 22)	35 (34, 37)	68 (65, 70)	137 (133, 141)	301 (294, 308)	439 (429, 449)

disorder										
Pericardial Effusion	Cardiovascular	3 (3, 4)	3 (2, 3)	3 (3, 4)	5 (4, 6)	9 (8, 10)	17 (16, 18)	32 (30, 34)	52 (49, 55)	56 (53, 60)
Peripheral Arterial Disease	Cardiovascular	3 (2, 3)	4 (3, 4)	6 (5, 6)	12 (11, 13)	36 (34, 37)	118 (115, 121)	307 (302, 313)	637 (626, 647)	912 (898, 927)
Primary Pulmonary Hypertension	Cardiovascular	5 (5, 6)	--	2 (1, 2)	3 (2, 3)	5 (5, 6)	11 (10, 12)	27 (25, 28)	71 (67, 74)	119 (114, 124)
Pulmonary Embolism	Cardiovascular	--	3 (3, 4)	17 (16, 18)	32 (30, 33)	61 (59, 63)	100 (97, 103)	185 (181, 190)	325 (317, 332)	403 (394, 413)
Raynauds Disease	Cardiovascular	4 (3, 4)	52 (49, 54)	71 (69, 74)	86 (84, 88)	109 (106, 112)	131 (128, 134)	160 (156, 164)	181 (175, 186)	167 (161, 173)
Rheumatic Valve Disorder	Cardiovascular	--	--	3 (2, 3)	4 (4, 5)	6 (6, 7)	14 (12, 15)	34 (32, 36)	84 (81, 88)	123 (118, 128)
Right Bundle Branch Block	Cardiovascular	--	6 (5, 7)	9 (8, 10)	11 (10, 11)	17 (16, 18)	34 (32, 35)	72 (69, 75)	182 (176, 187)	402 (393, 412)
Secondary Pulmonary Hypertension	Cardiovascular	--	--	--	--	3 (3, 4)	8 (7, 9)	17 (16, 18)	45 (43, 48)	65 (61, 69)
Sick Sinus Syndrome	Cardiovascular	--	--	--	--	2 (2, 3)	5 (4, 6)	15 (14, 16)	52 (49, 55)	99 (95, 104)
Stable Angina	Cardiovascular	--	3 (2, 3)	6 (5, 7)	23 (22, 24)	122 (119, 125)	403 (397, 409)	913 (903, 923)	1771 (1755, 1788)	2290 (2268, 2313)
Stroke – not otherwise specified	Cardiovascular	--	--	3 (3, 3)	8 (8, 9)	26 (24, 27)	70 (67, 72)	173 (169, 177)	410 (402, 418)	812 (799, 825)
Subarachnoid Haemorrhage	Cardiovascular	--	--	4 (3, 4)	8 (8, 9)	19 (18, 20)	31 (30, 33)	43 (41, 45)	50 (47, 53)	50 (46, 53)
Subdural haematoma	Cardiovascular	--	--	--	--	4 (3, 4)	6 (6, 7)	14 (13, 15)	39 (36, 41)	96 (91, 101)
Supraventricular Tachycardia	Cardiovascular	7 (6, 8)	15 (14, 16)	28 (26, 29)	37 (35, 38)	55 (53, 57)	80 (77, 82)	130 (126, 133)	206 (200, 212)	256 (248, 263)
Transient Ischaemic Attack	Cardiovascular	--	--	4 (3, 4)	11 (10, 12)	40 (38, 42)	119 (116, 122)	305 (300, 311)	736 (725, 747)	1293 (1276, 1310)
Trifascicular Block	Cardiovascular	--	--	--	--	--	--	3 (3, 4)	16 (15, 18)	55 (51, 59)
Unstable Angina	Cardiovascular	--	--	--	11 (10, 11)	56 (54, 58)	161 (158, 165)	311 (305, 316)	582 (572, 591)	798 (785, 812)
Venous thrombolism (Excl PE)	Cardiovascular	2 (2, 3)	7 (6, 8)	31 (30, 33)	70 (67, 72)	107 (104, 110)	166 (162, 170)	276 (271, 281)	444 (436, 452)	606 (594, 617)
Ventricular Tachycardia	Cardiovascular	--	--	3 (3, 4)	5 (4, 5)	9 (8, 10)	19 (17, 20)	39 (37, 40)	73 (69, 76)	88 (84, 93)
Abdominal Hernia	Digestive	292 (288, 297)	283 (278, 288)	294 (289, 298)	423 (417, 428)	625 (618, 631)	853 (844, 861)	1238 (1227, 1249)	1690 (1673, 1706)	2123 (2099, 2146)
Alcoholic Liver Disease	Digestive	--	--	5 (4, 5)	22 (21, 23)	47 (46, 49)	74 (72, 77)	72 (69, 75)	44 (42, 47)	19 (17, 21)
Anal Fissure	Digestive	83 (81, 85)	153 (149, 157)	240 (235, 244)	291 (286, 295)	314 (309, 318)	300 (295, 305)	266 (260, 271)	233 (227, 239)	169 (162, 175)
Angiodysplasia of colon	Digestive	--	--	--	--	3 (2, 3)	7 (7, 8)	17 (15, 18)	34 (32, 36)	38 (35, 41)
Anorectal Fistula	Digestive	5 (5, 6)	12 (11, 14)	29 (28, 31)	50 (48, 51)	63 (61, 65)	70 (68, 73)	65 (62, 67)	61 (57, 64)	50 (46, 53)
Anorectal Prolapse	Digestive	8 (8, 9)	10 (9, 11)	11 (10, 12)	14 (13, 15)	19 (18, 21)	30 (29, 32)	54 (52, 56)	82 (79, 86)	145 (140, 151)
Appendicitis	Digestive	41 (40, 43)	261 (257, 266)	418 (413, 423)	533 (527, 539)	689 (682, 696)	795 (787, 803)	854 (844, 863)	801 (790, 812)	678 (666, 690)
Autoimmune liver disease	Digestive	--	--	2 (2, 2)	2 (2, 3)	5 (5, 6)	10 (9, 11)	15 (14, 16)	21 (19, 23)	11 (9, 12)
Barrett's Oesophagus	Digestive	--	--	4 (3, 4)	10 (10, 11)	30 (29, 31)	68 (66, 71)	122 (119, 126)	166 (161, 171)	180 (174, 187)
Cholangitis	Digestive	--	--	3 (2, 3)	4 (4, 5)	8 (7, 9)	15 (14, 17)	31 (29, 33)	61 (58, 64)	108 (103, 113)
Cholecystitis	Digestive	--	12 (11, 13)	65 (63, 67)	117 (114, 120)	179 (175, 182)	234 (230, 239)	315 (309, 320)	383 (375, 391)	388 (379, 398)
Cholelithiasis	Digestive	6 (5, 7)	25 (23, 26)	115 (112, 117)	205 (201, 209)	323 (318, 328)	442 (436, 449)	621 (613, 628)	832 (821, 843)	958 (943, 972)
Cirrhosis	Digestive	--	--	5 (4, 6)	17 (16, 18)	40 (38, 42)	66 (64, 68)	73 (70, 76)	67 (64, 70)	38 (35, 40)
Coeliac Disease	Digestive	13 (12, 14)	22 (21, 24)	23 (22, 25)	26 (25, 27)	37 (35, 38)	43 (41, 45)	53 (50, 55)	57 (54, 60)	37 (35, 40)
Crohns Disease	Digestive	--	19 (18, 20)	39 (38, 41)	46 (44, 47)	52 (50, 54)	54 (52, 56)	56 (54, 59)	54 (51, 57)	46 (43, 49)
Diaphragmatic Hernia	Digestive	4 (3, 4)	18 (17, 19)	71 (68, 73)	158 (155, 161)	335 (330, 339)	611 (604, 618)	1005 (995, 1015)	1470 (1455, 1485)	1772 (1753, 1792)
Diverticular Disease	Digestive	--	--	9 (8, 10)	37 (36, 39)	149 (146, 152)	450 (444, 457)	992 (982, 1002)	1717 (1700, 1733)	2105 (2083, 2126)
Fatty Liver	Digestive	--	4 (3, 4)	12 (11, 13)	30 (29, 31)	52 (50, 54)	79 (76, 81)	77 (74, 80)	60 (57, 63)	30 (28, 33)
Gastritis	Digestive	66 (63, 68)	185 (181, 189)	303 (298, 307)	412 (406, 417)	615 (608, 621)	840 (831, 848)	1101 (1090, 1111)	1451 (1436, 1466)	1460 (1442, 1478)

Gastro-oesophageal Reflux Disease	Digestive	502 (497, 508)	220 (215, 224)	363 (358, 368)	608 (602, 615)	935 (926, 943)	1255 (1245, 1266)	1614 (1601, 1626)	1869 (1852, 1886)	1699 (1680, 1719)
Irritable Bowel Syndrome	Digestive	8 (7, 8)	186 (182, 191)	549 (543, 555)	719 (712, 726)	839 (832, 847)	865 (856, 874)	840 (831, 849)	727 (717, 738)	528 (518, 539)
Liver Failure	Digestive	--	--	4 (4, 5)	8 (7, 9)	15 (14, 16)	23 (22, 24)	26 (25, 28)	29 (27, 32)	29 (26, 32)
Oesophageal Ulcer	Digestive	106 (104, 109)	67 (65, 70)	154 (151, 157)	307 (302, 312)	536 (530, 542)	810 (802, 819)	1115 (1104, 1126)	1373 (1358, 1388)	1415 (1397, 1433)
Oesophageal Varices	Digestive	--	--	2 (2, 2)	6 (5, 7)	14 (13, 15)	25 (24, 27)	30 (28, 31)	28 (26, 31)	17 (15, 18)
Pancreatitis	Digestive	--	6 (6, 7)	22 (21, 24)	39 (38, 41)	59 (57, 61)	77 (74, 79)	103 (100, 106)	140 (135, 144)	166 (160, 172)
Peptic Ulcer	Digestive	--	8 (7, 9)	30 (28, 31)	68 (66, 71)	155 (152, 159)	293 (288, 298)	471 (464, 478)	764 (753, 775)	976 (961, 991)
Peritonitis	Digestive	11 (10, 11)	22 (20, 23)	27 (26, 29)	33 (32, 35)	52 (50, 54)	77 (74, 80)	110 (107, 114)	159 (154, 164)	198 (192, 205)
Portal Hypertension	Digestive	--	--	2 (2, 3)	7 (6, 7)	15 (14, 16)	25 (23, 26)	26 (24, 27)	23 (21, 25)	10 (9, 12)
Ulcerative Colitis	Digestive	--	15 (14, 16)	41 (39, 43)	57 (55, 59)	70 (68, 73)	89 (86, 92)	115 (112, 118)	122 (118, 127)	112 (107, 117)
Volvulus	Digestive	--	--	3 (2, 3)	4 (3, 4)	7 (6, 7)	12 (11, 13)	21 (20, 23)	36 (33, 38)	57 (53, 60)
Deafness	Ear	301 (296, 305)	556 (549, 563)	480 (474, 486)	385 (380, 390)	529 (523, 535)	795 (786, 803)	1285 (1274, 1296)	2094 (2075, 2112)	3321 (3294, 3348)
Meniere's Disease	Ear	--	--	3 (3, 4)	11 (10, 12)	22 (21, 23)	45 (43, 47)	76 (73, 78)	110 (106, 114)	143 (138, 149)
Tinnitus	Ear	6 (6, 7)	44 (42, 46)	96 (93, 98)	179 (175, 182)	284 (280, 288)	492 (486, 499)	661 (653, 669)	720 (709, 730)	670 (657, 682)
Cystic Fibrosis	Endocrine	5 (4, 5)	5 (4, 5)	4 (3, 4)	3 (3, 3)	5 (4, 5)	5 (4, 6)	7 (6, 8)	5 (4, 6)	--
Diabetes Mellitus – other or not specified	Endocrine	--	5 (5, 6)	12 (11, 12)	22 (21, 24)	30 (28, 31)	43 (41, 45)	61 (58, 63)	90 (86, 93)	97 (92, 101)
Hyperparathyroidism	Endocrine	--	--	3 (2, 3)	5 (5, 6)	12 (11, 13)	23 (21, 24)	41 (39, 43)	73 (70, 77)	90 (86, 94)
Low HDL-C	Endocrine	--	17 (16, 19)	77 (74, 79)	278 (273, 282)	688 (682, 695)	1052 (1042, 1061)	1440 (1428, 1452)	1728 (1712, 1745)	1303 (1286, 1320)
Obesity	Endocrine	--	804 (786, 822)	953 (945, 961)	1470 (1460, 1480)	2154 (2142, 2166)	2639 (2623, 2654)	2969 (2952, 2987)	2973 (2952, 2995)	1894 (1874, 1915)
Polycystic Ovarian Syndrome	Endocrine	--	86 (82, 90)	270 (264, 276)	241 (235, 246)	86 (83, 90)	20 (18, 22)	7 (6, 8)	--	--
Raised LDL-C	Endocrine	--	14 (13, 15)	88 (86, 91)	436 (430, 442)	1452 (1442, 1462)	2746 (2730, 2761)	3698 (3679, 3718)	3683 (3659, 3707)	2595 (2571, 2618)
Raised Total Cholesterol	Endocrine	--	21 (20, 23)	130 (127, 133)	649 (642, 656)	2205 (2192, 2217)	4172 (4153, 4191)	5629 (5606, 5653)	5941 (5911, 5971)	4772 (4740, 4804)
Raised Triglycerides	Endocrine	--	11 (10, 12)	64 (62, 66)	312 (307, 317)	966 (958, 974)	1900 (1887, 1912)	2712 (2696, 2729)	2971 (2949, 2992)	2016 (1995, 2037)
Syndrome of Inappropriate AntiDiuretic Hormone	Endocrine	--	--	--	--	--	3 (2, 3)	7 (6, 8)	21 (19, 23)	43 (40, 46)
Thyroid Disease	Endocrine	13 (13, 14)	52 (50, 54)	144 (141, 147)	298 (294, 303)	468 (462, 474)	710 (702, 718)	943 (934, 953)	1225 (1212, 1239)	1466 (1449, 1483)
Type 1 Diabetes Mellitus	Endocrine	17 (16, 18)	47 (45, 49)	48 (46, 50)	51 (49, 53)	55 (53, 57)	49 (47, 51)	38 (36, 40)	33 (31, 36)	23 (21, 25)
Type 2 Diabetes Mellitus	Endocrine	--	5 (4, 6)	29 (27, 30)	126 (124, 129)	382 (376, 387)	820 (811, 828)	1323 (1311, 1334)	1936 (1919, 1954)	1761 (1741, 1781)
Anterior Uveitis	Eye	3 (2, 3)	12 (11, 13)	27 (26, 28)	49 (48, 51)	75 (73, 78)	105 (102, 108)	123 (120, 127)	147 (142, 151)	157 (151, 162)
Blindness	Eye	18 (17, 19)	32 (30, 33)	35 (34, 37)	37 (35, 38)	55 (53, 57)	82 (79, 85)	135 (131, 139)	308 (301, 315)	874 (861, 888)
Cataract	Eye	9 (8, 10)	16 (15, 17)	19 (18, 20)	33 (32, 35)	92 (90, 95)	297 (292, 302)	946 (936, 956)	2755 (2734, 2775)	5083 (5050, 5116)
Diabetic Eye Disease	Eye	--	11 (10, 12)	24 (22, 25)	51 (49, 53)	121 (118, 124)	252 (248, 257)	424 (418, 431)	685 (675, 695)	609 (598, 621)
Glaucoma	Eye	2 (2, 2)	4 (3, 4)	5 (5, 6)	14 (13, 15)	48 (46, 50)	130 (126, 133)	319 (313, 325)	666 (656, 677)	1167 (1151, 1183)
Keratitis	Eye	5 (4, 6)	17 (16, 18)	31 (30, 33)	44 (43, 46)	56 (54, 58)	64 (61, 66)	75 (72, 77)	95 (91, 99)	114 (109, 119)

Macular Degeneration	Eye	--	--	3 (3, 4)	7 (7, 8)	18 (17, 19)	51 (49, 53)	164 (160, 168)	523 (514, 532)	1348 (1331, 1365)
Posterior Uveitis	Eye	--	--	3 (2, 3)	4 (4, 5)	6 (6, 7)	8 (7, 9)	9 (8, 10)	12 (10, 13)	12 (10, 14)
Ptosis	Eye	17 (16, 18)	22 (21, 24)	17 (16, 18)	17 (16, 18)	24 (23, 25)	38 (37, 40)	63 (61, 66)	99 (95, 103)	121 (116, 126)
Reitnal Detachment	Eye	--	5 (4, 6)	11 (10, 11)	20 (18, 21)	37 (36, 39)	72 (70, 75)	128 (124, 132)	166 (161, 171)	168 (162, 174)
Retinal Vascular Occlusion	Eye	--	--	--	3 (2, 3)	9 (8, 10)	27 (25, 28)	71 (69, 74)	165 (160, 170)	272 (265, 280)
Scleritis	Eye	5 (5, 6)	16 (15, 17)	19 (18, 20)	32 (30, 33)	56 (54, 58)	75 (72, 77)	79 (76, 81)	76 (72, 79)	58 (54, 61)
Acute Kidney Injury	Genitourinary	4 (3, 4)	8 (7, 9)	16 (15, 17)	24 (23, 26)	46 (44, 48)	103 (100, 106)	249 (244, 254)	696 (686, 707)	1644 (1625, 1663)
Benign Prostatic Hyperplasia	Genitourinary	--	--	4 (3, 5)	17 (16, 19)	112 (108, 116)	546 (537, 556)	1636 (1618, 1654)	3099 (3066, 3131)	4201 (4151, 4251)
Chronic Cystitis	Genitourinary	--	--	7 (6, 7)	10 (9, 10)	14 (13, 15)	22 (21, 23)	39 (37, 40)	63 (60, 66)	65 (61, 69)
Chronic Kidney Disease	Genitourinary	--	--	3 (3, 4)	9 (8, 10)	33 (31, 34)	140 (136, 143)	611 (603, 619)	1906 (1889, 1923)	3209 (3183, 3235)
Dysmenorrhoea	Genitourinary	13 (12, 15)	903 (890, 915)	993 (982, 1005)	869 (858, 880)	919 (908, 930)	674 (663, 685)	282 (274, 290)	76 (71, 81)	24 (21, 27)
End Stage Renal Disease	Genitourinary	3 (2, 3)	4 (3, 4)	7 (6, 7)	10 (10, 11)	18 (17, 19)	30 (28, 32)	46 (44, 48)	89 (85, 93)	120 (115, 126)
Endometrial Hyperplasia	Genitourinary	--	--	11 (10, 13)	41 (39, 44)	124 (120, 128)	162 (157, 168)	131 (125, 136)	89 (84, 94)	45 (41, 49)
Endometriosis	Genitourinary	--	28 (26, 30)	166 (161, 171)	379 (371, 386)	511 (503, 520)	373 (365, 381)	187 (181, 193)	93 (87, 98)	48 (44, 52)
Erectile Dysfunction	Genitourinary	--	34 (31, 36)	181 (175, 186)	348 (341, 355)	814 (804, 825)	1551 (1535, 1568)	2306 (2284, 2328)	2227 (2199, 2254)	1084 (1059, 1109)
Female Infertility	Genitourinary	--	14 (12, 15)	215 (209, 220)	598 (589, 608)	575 (566, 585)	305 (297, 312)	134 (128, 139)	58 (54, 62)	24 (21, 27)
Glomerulonephritis	Genitourinary	9 (8, 9)	17 (16, 18)	19 (18, 20)	26 (25, 28)	36 (34, 37)	52 (50, 54)	90 (87, 93)	198 (193, 204)	402 (393, 412)
Hydrocele	Genitourinary	207 (202, 213)	238 (232, 245)	179 (174, 184)	130 (125, 134)	131 (127, 135)	156 (151, 161)	222 (216, 229)	335 (325, 346)	435 (419, 451)
Male infertility	Genitourinary	--	--	51 (49, 54)	140 (136, 145)	136 (132, 140)	85 (81, 88)	44 (41, 47)	14 (12, 16)	--
Menorrhagia	Genitourinary	20 (19, 22)	846 (833, 858)	994 (982, 1006)	1470 (1456, 1485)	2788 (2768, 2808)	2654 (2632, 2675)	1410 (1393, 1427)	487 (475, 499)	191 (183, 199)
Neuropahtic Bladder	Genitourinary	10 (9, 11)	25 (23, 26)	32 (31, 34)	43 (41, 45)	81 (78, 83)	126 (123, 129)	191 (187, 196)	255 (248, 261)	259 (251, 266)
Obstructive and reflux uropathy	Genitourinary	44 (42, 45)	58 (56, 61)	49 (47, 51)	48 (47, 50)	60 (58, 62)	77 (75, 80)	117 (114, 121)	199 (193, 204)	272 (264, 280)
Postcoital Bleeding	Genitourinary	--	106 (102, 111)	391 (383, 398)	451 (443, 459)	476 (468, 484)	342 (334, 350)	166 (160, 172)	61 (57, 66)	14 (12, 17)
Postmenopausal Bleeding	Genitourinary	--	--	--	5 (4, 5)	127 (123, 131)	833 (821, 845)	1170 (1155, 1185)	1051 (1034, 1069)	687 (672, 703)
Tubulo-interstitial Nephropathy	Genitourinary	6 (5, 6)	32 (30, 33)	53 (51, 55)	38 (37, 40)	31 (30, 33)	28 (26, 29)	28 (26, 30)	35 (32, 37)	34 (31, 36)
Undescended Testis	Genitourinary	251 (245, 257)	195 (190, 201)	126 (122, 130)	67 (64, 70)	50 (47, 52)	39 (37, 42)	28 (25, 30)	14 (12, 16)	--
Urinary Incontinence	Genitourinary	81 (79, 83)	117 (114, 120)	96 (93, 99)	184 (181, 188)	388 (382, 393)	536 (529, 543)	673 (665, 681)	881 (869, 892)	1187 (1171, 1202)
Urolithiasis	Genitourinary	3 (3, 4)	25 (23, 26)	94 (91, 96)	172 (169, 176)	270 (266, 274)	349 (344, 355)	437 (431, 444)	493 (484, 502)	454 (443, 465)
Uterovaginal Prolapse	Genitourinary	--	--	40 (38, 42)	166 (161, 171)	400 (392, 407)	736 (725, 748)	1221 (1205, 1236)	1531 (1510, 1552)	1524 (1501, 1547)
Agranulocytosis	Haem/Imm	19 (18, 20)	25 (24, 26)	25 (23, 26)	33 (31, 34)	51 (49, 53)	85 (82, 87)	120 (116, 123)	128 (124, 133)	80 (76, 84)
Anaemia – other	Haem/Imm	51 (49, 52)	108 (105, 111)	216 (212, 220)	318 (314, 323)	431 (426, 437)	489 (482, 495)	649 (641, 657)	1271 (1257, 1285)	2194 (2172, 2216)
Aplastic Anaemia	Haem/Imm	2 (2, 3)	4 (3, 4)	3 (3, 4)	4 (4, 5)	6 (6, 7)	12 (11, 13)	21 (20, 23)	35 (33, 37)	46 (43, 50)
Folate Deficiency Anaemia	Haem/Imm	--	--	3 (2, 3)	5 (4, 6)	9 (8, 10)	14 (13, 15)	21 (20, 23)	43 (41, 46)	110 (105, 115)
Hypersplenism	Haem/Imm	6 (5, 7)	8 (8, 9)	10 (9, 11)	11 (10, 12)	13 (12, 14)	17 (16, 19)	23 (22, 25)	27 (25, 29)	22 (19, 24)
Hyposplenism	Haem/Imm	--	2 (2, 3)	6 (6, 7)	9 (9, 10)	16 (15, 17)	22 (21, 23)	27 (25, 28)	30 (28, 32)	25 (23, 27)
Immunodeficiency	Haem/Imm	6 (6, 7)	8 (7, 9)	4 (4, 5)	3 (3, 4)	4 (4, 5)	5 (5, 6)	8 (7, 9)	10 (9, 11)	9 (8, 11)

Iron Deficiency Anaemia	Haem/Imm	66 (64, 68)	149 (146, 153)	230 (226, 234)	320 (315, 324)	434 (429, 440)	462 (456, 469)	521 (514, 528)	925 (913, 937)	1441 (1424, 1459)
Other haemolytic anaemia	Haem/Imm	10 (9, 11)	9 (8, 10)	11 (10, 12)	11 (10, 12)	10 (9, 11)	9 (8, 10)	12 (11, 13)	18 (16, 20)	22 (20, 24)
Primary thrombocytopaenia	Haem/Imm	8 (7, 9)	11 (10, 12)	11 (10, 12)	12 (11, 13)	13 (12, 14)	15 (14, 16)	20 (18, 21)	29 (27, 31)	37 (34, 40)
Sarcoidosis	Haem/Imm	--	--	3 (3, 4)	12 (11, 13)	25 (24, 27)	36 (35, 38)	39 (37, 41)	38 (35, 40)	22 (20, 24)
Secondary Polycythaemia	Haem/Imm	--	--	2 (2, 2)	4 (3, 4)	9 (8, 10)	16 (15, 17)	22 (20, 23)	24 (22, 26)	18 (16, 20)
Secondary Thrombocytopaenia	Haem/Imm	11 (10, 12)	13 (11, 14)	22 (21, 23)	29 (28, 31)	31 (30, 33)	44 (42, 46)	67 (64, 70)	107 (103, 111)	135 (130, 141)
Sickle Cell Disease	Haem/Imm	5 (4, 6)	3 (2, 3)	3 (3, 3)	4 (3, 4)	3 (2, 3)	--	--	--	--
Sickle Cell Trait	Haem/Imm	18 (17, 19)	12 (11, 13)	19 (18, 20)	25 (23, 26)	21 (20, 22)	10 (9, 11)	5 (4, 6)	6 (5, 7)	--
Thalassaemia	Haem/Imm	4 (3, 4)	4 (4, 5)	8 (7, 8)	8 (8, 9)	7 (6, 8)	5 (4, 6)	4 (4, 5)	--	--
Thalassaemia Trait	Haem/Imm	6 (5, 7)	12 (11, 13)	20 (18, 21)	24 (23, 25)	17 (16, 18)	14 (12, 15)	9 (8, 10)	10 (9, 11)	6 (5, 7)
Thrombophilia	Haem/Imm	--	4 (4, 5)	12 (11, 13)	20 (19, 21)	19 (18, 20)	14 (13, 15)	13 (12, 14)	9 (8, 10)	--
Vitamin B12 deficiency anaemia	Haem/Imm	--	4 (3, 4)	12 (11, 13)	21 (19, 22)	32 (30, 33)	45 (43, 47)	71 (68, 73)	149 (144, 154)	271 (263, 278)
Bacterial Infection	Infections	487 (481, 492)	464 (457, 470)	602 (595, 608)	716 (709, 724)	796 (788, 803)	857 (848, 865)	1149 (1139, 1160)	2045 (2027, 2063)	3881 (3853, 3910)
Chronic Hepatitis	Infections	--	3 (3, 4)	19 (18, 20)	41 (39, 43)	46 (44, 48)	40 (38, 42)	22 (21, 24)	13 (12, 15)	7 (6, 8)
Encephalitis	Infections	2 (2, 2)	3 (2, 3)	--	--	2 (2, 3)	--	4 (3, 4)	5 (4, 6)	--
Fungal Infection	Infections	37 (36, 39)	30 (28, 32)	50 (48, 52)	49 (47, 51)	44 (43, 46)	59 (57, 62)	103 (99, 106)	175 (170, 180)	265 (257, 272)
HIV	Infections	--	--	7 (6, 7)	18 (17, 19)	21 (20, 22)	12 (11, 13)	5 (4, 6)	--	--
Infection – Anorectal	Infections	5 (4, 6)	12 (11, 13)	35 (33, 36)	55 (53, 57)	57 (55, 59)	49 (47, 52)	37 (35, 39)	27 (25, 29)	17 (15, 19)
Infection – Bone	Infections	10 (9, 11)	13 (12, 14)	11 (10, 12)	14 (13, 15)	22 (21, 23)	32 (31, 34)	43 (41, 45)	71 (68, 74)	89 (85, 94)
Infection – Digestive System	Infections	380 (374, 385)	219 (215, 224)	117 (114, 120)	113 (110, 116)	134 (131, 137)	186 (182, 190)	276 (271, 281)	483 (474, 491)	757 (744, 770)
Infection – Ear/Upper Respiratory Tract	Infections	1209 (1200, 1218)	1010 (1001, 1019)	354 (349, 358)	197 (193, 201)	154 (151, 158)	132 (128, 135)	132 (129, 136)	139 (135, 144)	146 (140, 151)
Infection – Eye	Infections	96 (94, 99)	34 (32, 36)	7 (6, 7)	7 (6, 7)	8 (7, 8)	10 (9, 11)	15 (14, 16)	34 (32, 37)	88 (84, 92)
Infection – Heart	Infections	--	--	3 (3, 4)	4 (4, 5)	5 (5, 6)	6 (5, 7)	9 (8, 10)	14 (13, 16)	14 (12, 16)
Infection – Liver	Infections	--	4 (3, 4)	14 (13, 15)	30 (29, 32)	34 (33, 36)	32 (30, 33)	22 (21, 24)	21 (19, 22)	22 (19, 24)
Infection – Lower Respiratory Tract	Infections	634 (627, 641)	295 (290, 300)	141 (138, 145)	191 (188, 195)	270 (266, 274)	416 (410, 422)	754 (746, 763)	1566 (1550, 1582)	3207 (3180, 3234)
Infection – Male Genitourinary	Infections	42 (40, 44)	69 (66, 73)	60 (57, 63)	50 (48, 53)	59 (57, 62)	57 (54, 60)	69 (66, 73)	100 (94, 106)	105 (97, 113)
Infection – Other Genitourianry	Infections	2 (2, 3)	16 (15, 17)	51 (49, 53)	47 (46, 49)	26 (25, 27)	15 (14, 16)	10 (9, 12)	12 (11, 14)	19 (17, 20)
Infection – Other nervous system	Infections	3 (3, 4)	5 (5, 6)	5 (5, 6)	6 (5, 7)	8 (7, 9)	11 (10, 12)	18 (17, 20)	26 (24, 28)	35 (32, 37)
Infection – Other organisms	Infections	1416 (1406, 1426)	1166 (1156, 1176)	494 (488, 500)	399 (394, 405)	456 (450, 462)	600 (593, 607)	948 (938, 958)	1706 (1689, 1722)	2978 (2952, 3003)
Infection – Other organs	Infections	662 (655, 669)	345 (340, 351)	206 (203, 210)	217 (213, 221)	208 (204, 212)	230 (226, 234)	310 (305, 316)	477 (468, 486)	598 (586, 609)
Infection – Skin	Infections	170 (166, 173)	170 (166, 174)	192 (188, 196)	216 (213, 220)	237 (233, 241)	270 (265, 275)	343 (337, 349)	544 (535, 553)	966 (952, 981)
Meningitis	Infections	15 (14, 17)	13 (12, 14)	17 (15, 18)	15 (14, 16)	12 (11, 13)	8 (7, 9)	6 (5, 7)	6 (5, 7)	--
Parasitic Infection	Infections	9 (8, 10)	18 (17, 19)	12 (11, 13)	10 (10, 11)	12 (11, 13)	12 (11, 13)	11 (10, 12)	12 (11, 14)	18 (16, 20)
Pelvic Inflammatory Disease	Infections	--	36 (34, 39)	176 (171, 181)	328 (321, 335)	386 (379, 393)	248 (241, 254)	136 (131, 141)	92 (87, 97)	52 (48, 56)
Rheumatic Fever	Infections	--	3 (2, 3)	4 (3, 4)	8 (7, 8)	13 (12, 14)	33 (31, 34)	71 (69, 74)	134 (130, 139)	173 (167, 179)
Septicaemia	Infections	164 (161, 167)	39 (38, 41)	23 (22, 24)	31 (30, 33)	52 (50, 54)	89 (86, 91)	169 (165, 173)	331 (324, 338)	596 (584, 607)
Tuberculosis	Infections	6 (6, 7)	16 (15, 17)	30 (29, 32)	45 (43, 47)	45 (43, 47)	64 (61, 66)	107 (103, 110)	199 (193, 204)	213 (206, 220)
Urinary Tract Infection	Infections	132 (129, 135)	150 (146, 153)	161 (158, 164)	156 (152, 159)	185 (182, 189)	257 (252, 262)	479 (472, 486)	1186 (1173, 1200)	2921 (2896, 2946)
Viral Infection	Infections	1244 (1234, 1253)	558 (551, 565)	204 (200, 208)	178 (175, 182)	150 (147, 154)	132 (128, 135)	136 (133, 140)	188 (183, 194)	301 (293, 309)

Ankylosing Spondylosis	Musculoskeletal	--	--	8 (7, 9)	15 (14, 16)	22 (21, 24)	30 (28, 31)	38 (36, 40)	32 (30, 34)	29 (27, 32)
Carpal Tunnel Syndrome	Musculoskeletal	--	11 (10, 12)	76 (74, 79)	228 (224, 232)	427 (421, 432)	619 (612, 627)	719 (710, 727)	722 (711, 732)	759 (746, 771)
Collapsed Vertebra	Musculoskeletal	--	--	3 (3, 4)	5 (4, 5)	8 (8, 9)	19 (18, 21)	43 (41, 45)	103 (99, 107)	197 (191, 203)
Enteropathic Arthropathy	Musculoskeletal	--	--	--	--	--	--	--	--	--
Enthesopathy	Musculoskeletal	60 (58, 62)	300 (295, 306)	582 (576, 589)	1121 (1112, 1130)	2260 (2248, 2273)	3188 (3171, 3205)	3517 (3498, 3536)	3357 (3334, 3380)	2796 (2771, 2821)
Fibromatosis	Musculoskeletal	--	--	4 (3, 5)	12 (11, 13)	46 (44, 48)	141 (138, 145)	288 (283, 294)	378 (370, 386)	340 (331, 350)
Fracture – Hip	Musculoskeletal	3 (3, 4)	9 (8, 10)	13 (12, 13)	16 (15, 17)	24 (22, 25)	48 (46, 51)	110 (107, 113)	370 (363, 378)	1243 (1227, 1258)
Fracture – Wrist	Musculoskeletal	123 (120, 126)	393 (387, 399)	312 (308, 317)	226 (223, 230)	221 (217, 225)	266 (261, 271)	352 (346, 358)	533 (524, 542)	810 (798, 822)
Giant Cell Arteritis	Musculoskeletal	--	--	--	--	3 (2, 3)	10 (9, 11)	30 (28, 32)	77 (73, 80)	116 (112, 121)
Gout	Musculoskeletal	--	2 (2, 3)	22 (21, 24)	90 (88, 93)	213 (209, 217)	408 (402, 414)	643 (635, 651)	973 (960, 985)	1135 (1118, 1151)
Intervertebral Disc Disorder	Musculoskeletal	--	12 (11, 13)	68 (66, 70)	211 (207, 215)	396 (391, 402)	509 (502, 516)	587 (579, 595)	640 (630, 650)	558 (547, 570)
Juvenile Arthritis	Musculoskeletal	7 (6, 7)	15 (14, 16)	11 (10, 12)	7 (7, 8)	5 (5, 6)	3 (3, 4)	--	--	--
Lupus Erythematosus	Musculoskeletal	--	3 (2, 3)	7 (6, 8)	13 (12, 14)	21 (20, 23)	26 (25, 28)	28 (27, 30)	26 (24, 28)	19 (17, 21)
Osteoarthritis	Musculoskeletal	--	10 (9, 11)	50 (48, 52)	166 (162, 169)	586 (580, 593)	1561 (1549, 1572)	2868 (2851, 2885)	4188 (4163, 4214)	4848 (4816, 4880)
Osteoporosis	Musculoskeletal	2 (2, 2)	6 (5, 7)	11 (10, 12)	22 (21, 24)	61 (59, 63)	228 (224, 233)	569 (561, 576)	1153 (1140, 1166)	1794 (1776, 1813)
Polymyalgia Rheumatica	Musculoskeletal	--	--	--	2 (2, 3)	9 (8, 9)	40 (39, 42)	142 (138, 146)	402 (394, 410)	614 (603, 625)
Psoriatic Arthritis	Musculoskeletal	--	--	7 (7, 8)	19 (18, 20)	33 (31, 34)	43 (42, 45)	45 (43, 47)	38 (35, 40)	20 (18, 23)
Reactive Arthritis	Musculoskeletal	6 (6, 7)	6 (5, 7)	7 (6, 7)	11 (10, 11)	14 (13, 15)	16 (15, 17)	15 (14, 16)	10 (9, 11)	7 (6, 9)
Rheumatoid Arthritis	Musculoskeletal	--	6 (5, 6)	17 (16, 18)	38 (36, 39)	80 (77, 82)	149 (146, 153)	240 (235, 245)	336 (328, 343)	370 (361, 378)
Scleroderma	Musculoskeletal	--	--	--	--	4 (3, 4)	7 (6, 8)	10 (9, 11)	11 (10, 12)	7 (6, 8)
Scoliosis	Musculoskeletal	13 (12, 14)	74 (72, 77)	64 (62, 66)	63 (61, 65)	59 (57, 61)	65 (63, 67)	91 (88, 94)	146 (142, 151)	228 (222, 235)
Sjogren Syndrome	Musculoskeletal	--	--	--	4 (3, 4)	8 (7, 9)	16 (15, 17)	26 (24, 27)	30 (28, 32)	21 (19, 23)
Spinal Stenosis	Musculoskeletal	--	--	4 (3, 4)	14 (13, 15)	38 (36, 39)	83 (80, 85)	164 (160, 168)	301 (294, 308)	309 (300, 317)
Spondylolisthesis	Musculoskeletal	--	3 (2, 3)	5 (4, 6)	13 (12, 14)	25 (24, 26)	44 (42, 46)	74 (71, 77)	107 (103, 111)	111 (107, 116)
Spondylosis	Musculoskeletal	--	3 (2, 3)	17 (16, 18)	70 (68, 72)	230 (226, 234)	546 (539, 553)	1018 (1008, 1028)	1521 (1506, 1537)	1726 (1706, 1745)
Autonomic Neuropathy	Neurological	2 (2, 3)	7 (6, 7)	8 (8, 9)	13 (12, 14)	24 (22, 25)	34 (32, 35)	46 (44, 48)	62 (59, 65)	62 (58, 65)
Bell's Palsy	Neurological	7 (6, 8)	27 (25, 28)	43 (41, 44)	62 (60, 64)	79 (77, 81)	100 (97, 103)	118 (114, 121)	144 (139, 148)	148 (142, 153)
Cerebral Palsy	Neurological	22 (21, 24)	30 (28, 32)	22 (20, 23)	14 (13, 15)	14 (13, 15)	12 (11, 13)	10 (9, 11)	7 (6, 8)	--
Chronic Fatigue Syndrome	Neurological	2 (2, 3)	34 (32, 35)	81 (79, 83)	139 (136, 143)	209 (206, 213)	263 (258, 268)	238 (233, 243)	187 (181, 192)	126 (121, 131)
Diabetic Neuropathy	Neurological	--	--	2 (2, 2)	6 (6, 7)	18 (17, 20)	45 (43, 47)	84 (81, 87)	159 (154, 164)	154 (148, 160)
Epilepsy	Neurological	61 (59, 63)	134 (130, 137)	157 (154, 160)	169 (166, 173)	198 (195, 202)	201 (197, 205)	212 (208, 217)	253 (246, 259)	287 (279, 295)
Essential Tremor	Neurological	--	7 (7, 8)	10 (10, 11)	10 (9, 10)	12 (11, 13)	19 (17, 20)	44 (42, 46)	93 (89, 97)	92 (88, 97)
Idiopathic Intracranial Hypertension	Neurological	--	6 (5, 7)	9 (9, 10)	8 (8, 9)	6 (5, 6)	5 (4, 5)	3 (2, 4)	--	--
Migraine	Neurological	46 (44, 47)	500 (493, 506)	830 (822, 837)	906 (898, 914)	945 (937, 953)	895 (886, 903)	778 (769, 786)	590 (581, 600)	388 (379, 397)
Motor Neurone Disease	Neurological	--	--	--	--	2 (2, 3)	4 (4, 5)	10 (9, 11)	17 (15, 18)	14 (12, 16)
Multiple Sclerosis	Neurological	--	--	8 (8, 9)	23 (22, 24)	38 (37, 40)	49 (47, 51)	51 (49, 53)	34 (32, 36)	19 (17, 21)
Myasthenia Gravis	Neurological	--	--	--	2 (2, 2)	3 (2, 3)	4 (4, 5)	9 (8, 9)	13 (12, 14)	14 (12, 16)
Parkinson's Disease	Neurological	--	--	--	--	4 (4, 5)	18 (17, 20)	70 (67, 73)	214 (208, 220)	331 (322, 340)
Peripheral Neuropathy	Neurological	4 (4, 5)	17 (16, 19)	49 (47, 51)	110 (107, 113)	211 (208, 215)	326 (321, 331)	432 (425, 439)	529 (520, 539)	481 (470, 491)

Trigeminal Neuralgia	Neurological	--	--	10 (9, 10)	25 (24, 26)	51 (49, 53)	80 (78, 83)	108 (104, 111)	137 (132, 141)	151 (145, 156)
Congenital Septal Defect	Perinatal	85 (83, 88)	59 (57, 61)	38 (36, 40)	30 (29, 32)	27 (25, 28)	24 (22, 25)	22 (21, 24)	23 (21, 25)	17 (15, 19)
Down Syndrome	Perinatal	11 (10, 12)	9 (8, 10)	8 (7, 8)	7 (6, 8)	10 (9, 11)	8 (7, 9)	5 (4, 5)	--	--
High Birth Weight	Perinatal	86 (84, 89)	8 (7, 8)	--	--	--	--	--	--	--
Intrauterine Hypoxia	Perinatal	346 (341, 351)	90 (87, 92)	4 (3, 4)	--	--	--	--	--	--
Low Birth Weight	Perinatal	441 (435, 447)	79 (76, 81)	6 (5, 7)	2 (2, 3)	--	--	--	--	--
Neonatal Jaundice	Perinatal	690 (683, 697)	142 (139, 146)	16 (15, 17)	11 (10, 12)	2 (2, 2)	--	--	--	--
Patent Ductus Arteriosus	Perinatal	50 (48, 52)	18 (17, 19)	9 (8, 10)	4 (4, 5)	4 (3, 4)	3 (3, 4)	3 (2, 3)	--	--
Post-term Delivery	Perinatal	71 (69, 73)	8 (8, 9)	--	--	--	--	--	--	--
Premature Delivery	Perinatal	356 (351, 361)	168 (164, 172)	64 (62, 66)	26 (25, 27)	12 (11, 13)	4 (3, 4)	--	--	--
Respiratory Distress of the Newborn	Perinatal	298 (293, 302)	70 (68, 73)	16 (15, 17)	7 (6, 8)	--	--	--	--	--
Sepsis of the Newborn	Perinatal	134 (131, 137)	14 (13, 15)	--	--	--	--	--	--	--
Spina Bifida	Perinatal	4 (4, 5)	6 (5, 7)	10 (9, 10)	15 (14, 16)	18 (16, 19)	16 (15, 17)	14 (12, 15)	10 (9, 12)	6 (5, 7)
Alcohol Misuse	Psychiatric	--	74 (72, 77)	254 (250, 259)	330 (326, 335)	436 (431, 441)	467 (461, 474)	434 (428, 441)	300 (293, 307)	180 (173, 186)
Anxiety	Psychiatric	46 (45, 48)	525 (518, 532)	1167 (1158, 1176)	1472 (1462, 1482)	1704 (1694, 1715)	1773 (1761, 1786)	1744 (1730, 1757)	1607 (1591, 1622)	1434 (1417, 1451)
Autism	Psychiatric	108 (105, 111)	157 (154, 161)	41 (40, 43)	15 (14, 16)	12 (11, 13)	8 (7, 8)	4 (3, 4)	--	--
Bipolar Affective Disorder	Psychiatric	--	11 (10, 12)	38 (37, 40)	56 (54, 58)	66 (64, 68)	62 (60, 64)	59 (56, 61)	58 (55, 61)	44 (41, 47)
Delirium	Psychiatric	--	3 (3, 4)	4 (3, 4)	4 (4, 5)	8 (7, 8)	17 (16, 18)	47 (45, 49)	192 (187, 198)	610 (599, 622)
Dementia	Psychiatric	--	--	--	--	6 (5, 7)	27 (25, 28)	130 (126, 134)	737 (726, 748)	2451 (2429, 2474)
Depression	Psychiatric	3 (2, 3)	486 (479, 492)	1505 (1495, 1515)	2049 (2037, 2061)	2492 (2479, 2505)	2510 (2496, 2525)	2277 (2262, 2293)	2031 (2013, 2048)	2019 (1999, 2039)
Eating Disorders	Psychiatric	--	33 (31, 34)	60 (58, 62)	70 (68, 72)	50 (48, 52)	28 (26, 29)	12 (11, 14)	5 (4, 6)	--
Hyperkinetic Disorders	Psychiatric	55 (53, 57)	168 (164, 172)	59 (57, 61)	10 (9, 11)	5 (5, 6)	3 (3, 4)	--	--	--
Intellectual Disability	Psychiatric	42 (41, 44)	110 (107, 114)	89 (86, 91)	61 (59, 63)	70 (68, 72)	66 (63, 68)	53 (50, 55)	37 (35, 39)	20 (18, 22)
Obsessive Compulsive Disorder	Psychiatric	4 (4, 5)	41 (39, 42)	65 (63, 67)	66 (63, 68)	58 (56, 60)	47 (45, 49)	35 (33, 37)	26 (24, 28)	22 (20, 24)
Personality Disorder	Psychiatric	--	30 (29, 32)	69 (67, 71)	85 (83, 88)	95 (92, 97)	81 (78, 83)	72 (69, 75)	56 (54, 59)	48 (45, 51)
Schizophrenia	Psychiatric	--	19 (18, 20)	67 (65, 69)	92 (89, 94)	102 (100, 105)	97 (95, 100)	91 (88, 94)	102 (98, 106)	114 (109, 118)
Substance Misuse	Psychiatric	2 (2, 3)	90 (87, 93)	274 (270, 279)	329 (324, 334)	225 (221, 229)	128 (124, 131)	95 (92, 98)	90 (86, 93)	79 (75, 83)
Allergic/chronic Rhinitis	Respiratory	639 (633, 646)	1705 (1692, 1717)	1730 (1719, 1741)	1719 (1708, 1730)	1602 (1591, 1612)	1517 (1506, 1529)	1492 (1480, 1504)	1477 (1462, 1492)	1299 (1282, 1316)
Asbestosis	Respiratory	--	--	--	--	--	4 (4, 5)	25 (24, 27)	60 (57, 64)	63 (59, 68)
Aspiration Pneumonitis	Respiratory	5 (4, 6)	5 (4, 6)	6 (6, 7)	8 (7, 9)	13 (12, 14)	22 (21, 24)	49 (47, 51)	121 (116, 125)	341 (332, 350)
Asthma	Respiratory	798 (790, 805)	2040 (2026, 2053)	1970 (1959, 1982)	1562 (1552, 1573)	1408 (1398, 1418)	1333 (1322, 1344)	1362 (1351, 1374)	1536 (1521, 1552)	1427 (1410, 1445)
Bronchiectasis	Respiratory	4 (3, 4)	6 (5, 7)	7 (6, 7)	11 (10, 12)	21 (20, 22)	52 (49, 54)	128 (125, 132)	210 (204, 216)	208 (201, 215)
Chronic Obstructive Pulmonary Disease	Respiratory	4 (4, 5)	8 (7, 9)	12 (11, 13)	33 (31, 34)	127 (124, 130)	374 (368, 379)	826 (817, 835)	1360 (1346, 1375)	1574 (1555, 1593)
Chronic Sinusitis	Respiratory	8 (7, 9)	45 (43, 47)	110 (108, 113)	185 (182, 189)	285 (280, 289)	371 (365, 376)	393 (387, 399)	328 (321, 335)	216 (210, 223)
Hypertrophic Nasal Turbinates	Respiratory	8 (7, 8)	33 (31, 34)	48 (46, 50)	49 (47, 51)	49 (47, 51)	44 (42, 46)	35 (34, 37)	22 (21, 24)	10 (9, 12)
Nasal Polyps	Respiratory	7 (6, 8)	26 (25, 28)	44 (42, 46)	68 (66, 71)	104 (101, 106)	148 (144, 152)	205 (201, 210)	238 (232, 244)	217 (210, 225)
Pleural Effusion	Respiratory	13 (12, 14)	15 (14, 16)	23 (22, 24)	36 (35, 38)	64 (62, 66)	123 (119, 126)	250 (245, 255)	538 (529, 548)	1017 (1002, 1033)
Pleural Plaque	Respiratory	--	--	--	--	--	9 (8, 9)	42 (40, 45)	102 (98, 106)	126 (121, 132)

Pneumothorax	Respiratory	10 (9, 11)	17 (16, 18)	35 (33, 36)	43 (41, 44)	49 (47, 51)	59 (56, 61)	75 (72, 78)	88 (84, 91)	86 (81, 90)
Pulmonary Collapse	Respiratory	13 (12, 14)	10 (9, 11)	11 (10, 12)	16 (15, 17)	28 (27, 30)	56 (54, 58)	101 (98, 104)	170 (165, 176)	234 (226, 241)
Pulmonary Fibrosis	Respiratory	--	--	--	2 (2, 2)	6 (5, 7)	16 (14, 17)	45 (43, 47)	123 (119, 128)	174 (168, 181)
Respiratory Failure	Respiratory	7 (6, 7)	6 (6, 7)	10 (9, 10)	14 (13, 15)	29 (28, 31)	64 (61, 66)	147 (143, 150)	289 (282, 296)	436 (427, 446)
Sleep apnoea	Respiratory	84 (82, 87)	46 (44, 48)	26 (25, 27)	59 (57, 61)	122 (119, 124)	178 (174, 182)	185 (181, 190)	123 (118, 127)	48 (44, 51)
Acne	Skin	47 (45, 49)	1720 (1707, 1732)	1820 (1809, 1831)	1190 (1181, 1200)	633 (626, 639)	332 (326, 337)	153 (149, 157)	87 (83, 91)	49 (46, 53)
Actinic keratosis	Skin	--	--	3 (3, 4)	18 (17, 19)	78 (76, 80)	239 (235, 244)	586 (578, 594)	1035 (1022, 1048)	1337 (1319, 1355)
Alopecia Areata	Skin	10 (9, 11)	34 (32, 36)	43 (42, 45)	49 (47, 51)	49 (47, 51)	46 (44, 48)	38 (36, 40)	32 (29, 34)	22 (20, 24)
Dermatitis	Skin	3312 (3296, 3327)	3283 (3266, 3300)	2278 (2266, 2291)	2010 (1999, 2022)	2136 (2124, 2148)	2290 (2276, 2305)	2480 (2464, 2496)	2789 (2768, 2810)	2907 (2881, 2932)
Hidradenitis	Skin	--	15 (13, 16)	36 (34, 37)	47 (45, 49)	48 (46, 49)	37 (35, 39)	20 (19, 22)	8 (7, 9)	--
Lichen Planus	Skin	--	6 (5, 7)	14 (13, 15)	30 (29, 32)	52 (50, 54)	94 (91, 97)	133 (130, 137)	149 (144, 154)	123 (118, 128)
Pilonidal cyst/sinus	Skin	19 (17, 20)	51 (49, 54)	134 (131, 137)	154 (151, 158)	120 (118, 123)	89 (86, 92)	71 (69, 74)	51 (48, 54)	32 (29, 34)
Psoriasis	Skin	25 (24, 26)	147 (143, 150)	272 (268, 277)	341 (336, 346)	409 (404, 414)	464 (458, 470)	521 (513, 528)	540 (531, 549)	447 (438, 457)
Rosacea	Skin	2 (2, 2)	79 (76, 81)	148 (145, 152)	207 (203, 210)	312 (307, 317)	388 (383, 394)	417 (411, 423)	394 (386, 402)	321 (313, 330)
Seborrheic Dermatitis	Skin	474 (468, 480)	499 (492, 505)	405 (400, 411)	391 (386, 396)	440 (435, 446)	504 (497, 511)	579 (572, 587)	688 (678, 699)	683 (670, 695)
Urticaria	Skin	514 (508, 520)	724 (716, 732)	521 (515, 527)	439 (433, 445)	489 (483, 494)	509 (503, 516)	517 (509, 524)	520 (511, 529)	427 (418, 437)
Vitiligo	Skin	15 (14, 16)	34 (32, 36)	38 (36, 39)	37 (35, 38)	41 (39, 43)	42 (40, 44)	43 (41, 45)	37 (34, 39)	28 (26, 31)

--Small numbers: to protect patient confidentiality, where prevalence counts were <100, period prevalence estimates have been replaced with "--".

Supplementary Table S5. Age-standardised period prevalence per 10000 (95% confidence intervals) from 1 April 2010 to 31 Mar 2015 for 308 medical conditions, stratified by sex.

Condition	Category	Female (Age-standardised)	Male (Age-standardised)	Both Sexes (Age- and sex-standardised)
Benign Neoplasm – Brain	Benign Neo	31 (30, 32)	20 (19, 21)	25 (25, 26)
Benign Neoplasm – Colon	Benign Neo	249 (246, 251)	330 (327, 333)	289 (287, 291)
Benign Neoplasm – Ovary	Benign Neo	383 (380, 386)	--	383 (380, 386)
Benign Neoplasm – Stomach	Benign Neo	65 (63, 66)	47 (46, 48)	56 (55, 56)
Benign Neoplasm – Uterus	Benign Neo	181 (179, 183)	--	181 (179, 183)
Cervical Intra-epithelial Neoplasia	Benign Neo	241 (239, 243)	--	241 (239, 243)
Haemangioma	Benign Neo	149 (147, 150)	106 (105, 108)	127 (126, 129)
Leiomyoma	Benign Neo	401 (398, 404)	--	401 (398, 404)
Hodgkins Lymphoma	Cancers	6 (6, 7)	8 (8, 8)	7 (7, 7)
Leukaemia	Cancers	18 (17, 19)	27 (27, 28)	23 (22, 23)
Monoclonal Gammopathy of Undetermined Significance	Cancers	11 (11, 12)	14 (13, 14)	12 (12, 13)
Myelodysplastic Syndrome	Cancers	10 (9, 10)	13 (13, 14)	11 (11, 12)
Non Hodgkins Lymphoma	Cancers	25 (25, 26)	34 (33, 35)	30 (29, 30)
Plasma Cell Malignancy	Cancers	8 (8, 9)	13 (12, 13)	10 (10, 11)
Polycythaemia vera	Cancers	7 (7, 8)	15 (15, 16)	11 (11, 12)
Primary Malignancy – Biliary	Cancers	4 (3, 4)	4 (4, 5)	4 (4, 4)
Primary Malignancy – Bladder	Cancers	20 (19, 21)	73 (72, 74)	46 (46, 47)
Primary Malignancy – Bone	Cancers	3 (3, 4)	4 (4, 5)	4 (4, 4)
Primary Malignancy – Bowel	Cancers	67 (66, 68)	93 (91, 94)	80 (79, 81)
Primary Malignancy – Brain	Cancers	9 (8, 9)	10 (10, 11)	10 (9, 10)
Primary Malignancy – Breast	Cancers	274 (272, 277)	3 (2, 3)	139 (137, 140)
Primary Malignancy – Cervix	Cancers	19 (19, 20)	--	19 (19, 20)
Primary Malignancy – Kidney	Cancers	12 (12, 13)	23 (22, 24)	17 (17, 18)
Primary Malignancy – Liver	Cancers	3 (2, 3)	6 (6, 6)	4 (4, 5)
Primary Malignancy – Lung	Cancers	38 (37, 39)	53 (51, 54)	45 (44, 46)
Primary Malignancy – Melanoma	Cancers	57 (56, 58)	47 (46, 48)	52 (51, 53)
Primary Malignancy – Mesothelioma	Cancers	1 (1, 1)	5 (5, 6)	3 (3, 3)
Primary Malignancy – Multiple Sites	Cancers	1 (1, 1)	2 (2, 2)	2 (1, 2)
Primary Malignancy – Oesophageal	Cancers	7 (7, 8)	19 (18, 19)	13 (13, 13)
Primary Malignancy – Oropharyngeal	Cancers	12 (12, 13)	21 (21, 22)	17 (16, 17)
Primary Malignancy – other	Cancers	31 (30, 32)	36 (35, 37)	33 (33, 34)
Primary Malignancy – Ovary	Cancers	29 (28, 30)	--	29 (28, 30)
Primary Malignancy – Pancreas	Cancers	9 (9, 10)	11 (10, 11)	10 (10, 10)
Primary Malignancy – Prostate	Cancers	--	209 (207, 212)	209 (207, 212)
Primary Malignancy – Skin	Cancers	272 (270, 275)	351 (348, 354)	312 (310, 314)
Primary Malignancy – Stomach	Cancers	7 (6, 7)	15 (15, 16)	11 (11, 11)
Primary Malignancy – Testis	Cancers	--	16 (16, 17)	16 (16, 17)
Primary Malignancy – Thyroid	Cancers	10 (9, 10)	3 (3, 4)	7 (6, 7)
Primary Malignancy – Uterus	Cancers	37 (36, 38)	--	37 (36, 38)
Secondary Malignancy – Adrenal	Cancers	4 (4, 4)	6 (5, 6)	5 (5, 5)
Secondary Malignancy – Bone	Cancers	28 (27, 29)	44 (43, 45)	36 (35, 37)
Secondary Malignancy – Bowel	Cancers	4 (3, 4)	2 (2, 3)	3 (3, 3)
Secondary Malignancy – Brain	Cancers	11 (11, 12)	11 (11, 12)	11 (11, 12)
Secondary Malignancy – Liver	Cancers	37 (36, 38)	43 (42, 44)	40 (39, 41)
Secondary Malignancy – Lung	Cancers	26 (25, 27)	29 (28, 30)	28 (27, 28)
Secondary Malignancy – Lymph Nodes	Cancers	79 (78, 81)	50 (48, 51)	65 (64, 65)
Secondary Malignancy – other	Cancers	18 (17, 19)	18 (17, 19)	18 (18, 18)
Secondary Malignancy – Peritoneum	Cancers	19 (18, 20)	11 (10, 11)	15 (14, 15)
Secondary Malignancy – Pleura	Cancers	8 (8, 8)	6 (6, 7)	7 (7, 7)
Abdominal Aortic Aneurysm	Cardiovascular	21 (20, 22)	88 (86, 89)	54 (54, 55)
Atrial Fibrillation	Cardiovascular	349 (347, 352)	516 (513, 520)	433 (431, 435)
Atrioventricular Block, first degree	Cardiovascular	22 (21, 23)	49 (47, 50)	35 (35, 36)
Atrioventricular Block, second degree	Cardiovascular	9 (9, 9)	17 (17, 18)	13 (13, 14)
Atrioventricular Block, third degree	Cardiovascular	14 (13, 14)	27 (26, 28)	20 (20, 21)
Bifascicular Block	Cardiovascular	2 (1, 2)	6 (5, 6)	4 (3, 4)
Cardiomyopathy – other	Cardiovascular	16 (16, 17)	37 (36, 38)	26 (26, 27)
Coronary Heart Disease (not otherwise specified)	Cardiovascular	89 (88, 91)	124 (122, 126)	107 (106, 108)
Dilated cardiomyopathy	Cardiovascular	8 (7, 8)	21 (21, 22)	15 (14, 15)
Heart Failure	Cardiovascular	228 (226, 230)	327 (324, 330)	278 (276, 279)
Hypertension	Cardiovascular	2000 (1994, 2007)	2125 (2118, 2132)	2063 (2058, 2068)
Hypertrophic cardiomyopathy	Cardiovascular	4 (4, 5)	7 (7, 7)	6 (5, 6)
Intracerebral Haemorrhage	Cardiovascular	27 (26, 28)	36 (35, 37)	31 (31, 32)

Ischaemic Stroke	Cardiovascular	116 (114, 117)	153 (151, 155)	134 (133, 135)
Left Bundle Branch Block	Cardiovascular	46 (45, 47)	65 (64, 66)	55 (55, 56)
Multiple valve disorder	Cardiovascular	53 (52, 54)	62 (61, 64)	58 (57, 58)
Myocardial Infarction	Cardiovascular	180 (178, 182)	419 (416, 422)	299 (297, 301)
Non-rheumatic Aortic valve disorder	Cardiovascular	84 (83, 85)	113 (111, 115)	99 (97, 100)
Non-rheumatic Mitral valve disorder	Cardiovascular	85 (83, 86)	84 (82, 85)	84 (83, 85)
Pericardial Effusion	Cardiovascular	15 (15, 16)	18 (17, 18)	16 (16, 17)
Peripheral Arterial Disease	Cardiovascular	115 (113, 116)	210 (207, 212)	162 (161, 164)
Primary Pulmonary Hypertension	Cardiovascular	19 (19, 20)	19 (18, 19)	19 (19, 19)
Pulmonary Embolism	Cardiovascular	103 (102, 105)	96 (94, 97)	100 (98, 101)
Raynauds Disease	Cardiovascular	138 (136, 140)	66 (65, 67)	102 (101, 103)
Rheumatic Valve Disorder	Cardiovascular	24 (23, 25)	19 (18, 20)	22 (21, 22)
Right Bundle Branch Block	Cardiovascular	34 (33, 35)	76 (74, 77)	55 (54, 56)
Secondary Pulmonary Hypertension	Cardiovascular	12 (11, 12)	11 (10, 11)	11 (11, 12)
Sick Sinus Syndrome	Cardiovascular	12 (11, 12)	14 (14, 15)	13 (12, 13)
Stable Angina	Cardiovascular	348 (345, 350)	561 (558, 565)	455 (452, 457)
Stroke – not otherwise specified	Cardiovascular	103 (101, 104)	122 (120, 124)	112 (111, 113)
Subarachnoid Haemorrhage	Cardiovascular	22 (22, 23)	19 (18, 20)	21 (20, 21)
Subdural haematoma	Cardiovascular	8 (7, 8)	16 (15, 17)	12 (12, 12)
Supraventricular Tachycardia	Cardiovascular	83 (82, 84)	68 (66, 69)	75 (74, 76)
Transient Ischaemic Attack	Cardiovascular	167 (165, 169)	213 (210, 215)	190 (188, 191)
Trifascicular Block	Cardiovascular	2 (1, 2)	8 (7, 8)	5 (4, 5)
Unstable Angina	Cardiovascular	112 (111, 114)	206 (204, 209)	159 (158, 161)
Venous thrombolism (Excl PE)	Cardiovascular	163 (161, 165)	144 (142, 146)	153 (152, 155)
Ventricular Tachycardia	Cardiovascular	12 (12, 13)	29 (28, 29)	20 (20, 21)
Abdominal Hernia	Digestive	339 (337, 342)	1175 (1170, 1180)	757 (754, 760)
Alcoholic Liver Disease	Digestive	20 (20, 21)	47 (46, 48)	33 (33, 34)
Anal Fissure	Digestive	258 (256, 260)	218 (216, 220)	238 (236, 239)
Angiodysplasia of colon	Digestive	8 (7, 8)	9 (9, 10)	8 (8, 9)
Anorectal Fistula	Digestive	33 (32, 34)	58 (57, 59)	46 (45, 46)
Anorectal Prolapse	Digestive	47 (46, 48)	19 (18, 19)	33 (32, 33)
Appendicitis	Digestive	605 (601, 609)	521 (518, 525)	563 (561, 566)
Autoimmune liver disease	Digestive	11 (10, 11)	3 (3, 4)	7 (7, 7)
Barrett's Oesophagus	Digestive	36 (35, 37)	71 (69, 72)	53 (53, 54)
Cholangitis	Digestive	18 (17, 18)	20 (19, 21)	19 (18, 19)
Cholecystitis	Digestive	235 (233, 238)	108 (107, 110)	172 (170, 173)
Cholelithiasis	Digestive	472 (469, 475)	216 (214, 218)	344 (342, 346)
Cirrhosis	Digestive	26 (25, 26)	42 (41, 43)	34 (33, 35)
Coeliac Disease	Digestive	44 (43, 45)	24 (23, 25)	34 (34, 35)
Crohns Disease	Digestive	44 (43, 45)	38 (37, 39)	41 (41, 42)
Diaphragmatic Hernia	Digestive	526 (523, 530)	470 (466, 473)	498 (496, 500)
Diverticular Disease	Digestive	494 (491, 498)	429 (426, 433)	462 (459, 464)
Fatty Liver	Digestive	35 (34, 36)	45 (44, 46)	40 (39, 40)
Gastritis	Digestive	650 (646, 653)	648 (644, 651)	649 (646, 651)
Gastro-oesophageal Reflux Disease	Digestive	995 (991, 1000)	888 (884, 892)	942 (938, 945)
Irritable Bowel Syndrome	Digestive	876 (871, 880)	338 (336, 341)	607 (604, 610)
Liver Failure	Digestive	12 (11, 12)	17 (16, 18)	14 (14, 15)
Oesophageal Ulcer	Digestive	570 (566, 573)	601 (598, 605)	585 (583, 588)
Oesophageal Varices	Digestive	10 (9, 10)	17 (16, 18)	13 (13, 14)
Pancreatitis	Digestive	59 (58, 60)	61 (60, 63)	60 (59, 61)
Peptic Ulcer	Digestive	189 (187, 191)	305 (302, 307)	247 (245, 249)
Peritonitis	Digestive	61 (59, 62)	71 (70, 72)	66 (65, 67)
Portal Hypertension	Digestive	9 (9, 10)	15 (15, 16)	12 (12, 13)
Ulcerative Colitis	Digestive	65 (64, 66)	67 (66, 68)	66 (65, 67)
Volvulus	Digestive	13 (12, 13)	12 (11, 12)	12 (12, 13)
Deafness	Ear	811 (807, 815)	961 (956, 966)	886 (883, 889)
Meniere's Disease	Ear	45 (44, 46)	29 (28, 30)	37 (36, 38)
Tinnitus	Ear	313 (310, 315)	331 (328, 334)	322 (320, 324)
Cystic Fibrosis	Endocrine	7 (6, 7)	2 (2, 3)	5 (4, 5)
Diabetes Mellitus – other or not specified	Endocrine	34 (33, 35)	36 (35, 37)	35 (34, 36)
Hyperparathyroidism	Endocrine	30 (29, 31)	13 (13, 14)	22 (21, 22)
Low HDL-C	Endocrine	635 (632, 639)	711 (707, 715)	673 (670, 676)
Obesity	Endocrine	1945 (1939, 1952)	1439 (1433, 1445)	1692 (1688, 1696)
Polycystic Ovarian Syndrome	Endocrine	90 (89, 91)	--	90 (89, 91)
Raised LDL-C	Endocrine	1595 (1589, 1601)	1468 (1462, 1474)	1531 (1527, 1536)
Raised Total Cholesterol	Endocrine	2530 (2523, 2538)	2266 (2259, 2273)	2398 (2393, 2403)
Raised Triglycerides	Endocrine	954 (949, 959)	1291 (1286, 1297)	1123 (1119, 1126)

Syndrome of Inappropriate AntiDiuretic Hormone	Endocrine	6 (6, 6)	5 (5, 6)	6 (5, 6)
Thyroid Disease	Endocrine	809 (805, 814)	227 (224, 229)	518 (516, 521)
Type 1 Diabetes Mellitus	Endocrine	36 (35, 37)	49 (48, 50)	42 (42, 43)
Type 2 Diabetes Mellitus	Endocrine	506 (503, 509)	693 (689, 698)	600 (597, 602)
Anterior Uveitis	Eye	71 (70, 72)	72 (70, 73)	71 (70, 72)
Blindness	Eye	121 (120, 123)	119 (118, 121)	120 (119, 121)
Cataract	Eye	722 (718, 725)	625 (621, 629)	673 (671, 676)
Diabetic Eye Disease	Eye	167 (165, 169)	239 (236, 241)	203 (201, 205)
Glaucoma	Eye	176 (174, 178)	189 (187, 191)	182 (181, 184)
Keratitis	Eye	56 (54, 57)	47 (46, 48)	51 (51, 52)
Macular Degeneration	Eye	160 (158, 162)	129 (127, 131)	144 (143, 146)
Posterior Uveitis	Eye	6 (6, 6)	5 (5, 6)	6 (5, 6)
Ptosis	Eye	43 (42, 44)	35 (34, 36)	39 (39, 40)
Reitnal Detachment	Eye	50 (49, 51)	66 (64, 67)	58 (57, 58)
Retinal Vascular Occlusion	Eye	38 (37, 39)	46 (45, 48)	42 (41, 43)
Scleritis	Eye	56 (55, 57)	35 (35, 36)	46 (45, 46)
Acute Kidney Injury	Genitourinary	165 (163, 167)	236 (233, 238)	200 (199, 202)
Benign Prostatic Hyperplasia	Genitourinary	--	769 (765, 774)	769 (765, 774)
Chronic Cystitis	Genitourinary	27 (26, 27)	15 (14, 15)	21 (20, 21)
Chronic Kidney Disease	Genitourinary	462 (458, 465)	393 (390, 397)	427 (425, 430)
Dysmenorrhoea	Genitourinary	597 (594, 601)	--	597 (594, 601)
End Stage Renal Disease	Genitourinary	23 (22, 23)	35 (34, 36)	29 (28, 29)
Endometrial Hyperplasia	Genitourinary	72 (70, 73)	--	72 (70, 73)
Endometriosis	Genitourinary	228 (226, 230)	--	228 (226, 230)
Erectile Dysfunction	Genitourinary	--	916 (911, 920)	916 (911, 920)
Female Infertility	Genitourinary	252 (249, 254)	--	252 (249, 254)
Glomerulonephritis	Genitourinary	59 (58, 60)	79 (77, 80)	69 (68, 70)
Hydrocele	Genitourinary	--	204 (202, 206)	204 (202, 206)
Male infertility	Genitourinary	--	62 (61, 63)	62 (61, 63)
Menorrhagia	Genitourinary	1377 (1372, 1382)	--	1377 (1372, 1382)
Neuropahtic Bladder	Genitourinary	123 (122, 125)	76 (74, 77)	100 (99, 101)
Obstructive and reflux uropathy	Genitourinary	73 (71, 74)	102 (100, 103)	87 (86, 88)
Postcoital Bleeding	Genitourinary	258 (255, 260)	--	258 (255, 260)
Postmenopausal Bleeding	Genitourinary	395 (392, 398)	--	395 (392, 398)
Tubulo-interstitial Nephropathy	Genitourinary	51 (50, 52)	13 (12, 13)	32 (31, 32)
Undescended Testis	Genitourinary	--	89 (88, 90)	89 (88, 90)
Urinary Incontinence	Genitourinary	663 (659, 666)	138 (136, 140)	400 (398, 403)
Urolithiasis	Genitourinary	154 (153, 156)	326 (323, 328)	240 (238, 242)
Uterovaginal Prolapse	Genitourinary	537 (534, 541)	--	537 (534, 541)
Agranulocytosis	Haem/Imm	69 (67, 70)	51 (50, 52)	60 (59, 61)
Anaemia – other	Haem/Imm	652 (649, 656)	370 (367, 373)	511 (509, 514)
Aplastic Anaemia	Haem/Imm	11 (10, 11)	13 (13, 14)	12 (12, 12)
Folate Deficiency Anaemia	Haem/Imm	17 (17, 18)	15 (14, 16)	16 (16, 17)
Hypersplenism	Haem/Imm	11 (11, 12)	18 (17, 18)	15 (14, 15)
Hyposplenism	Haem/Imm	12 (12, 13)	17 (17, 18)	15 (14, 15)
Immunodeficiency	Haem/Imm	6 (6, 6)	6 (6, 6)	6 (6, 6)
Iron Deficiency Anaemia	Haem/Imm	614 (610, 617)	252 (249, 254)	433 (430, 435)
Other haemolytic anaemia	Haem/Imm	13 (12, 13)	11 (10, 11)	12 (11, 12)
Primary thrombocytopaenia	Haem/Imm	16 (16, 17)	15 (14, 15)	15 (15, 16)
Sarcoidosis	Haem/Imm	20 (19, 20)	20 (19, 20)	20 (19, 20)
Secondary Polycythaemia	Haem/Imm	7 (6, 7)	13 (13, 14)	10 (9, 10)
Secondary Thrombocytopaenia	Haem/Imm	41 (40, 42)	46 (45, 47)	44 (43, 44)
Sickle Cell Disease	Haem/Imm	3 (3, 3)	2 (2, 3)	3 (3, 3)
Sickle Cell Trait	Haem/Imm	19 (19, 20)	10 (9, 10)	14 (14, 15)
Thalassaemia	Haem/Imm	7 (7, 7)	4 (4, 4)	5 (5, 6)
Thalassaemia Trait	Haem/Imm	17 (16, 18)	11 (10, 11)	14 (14, 14)
Thrombophilia	Haem/Imm	18 (17, 18)	6 (6, 6)	12 (11, 12)
Vitamin B12 deficiency anaemia	Haem/Imm	63 (62, 64)	38 (37, 39)	50 (50, 51)
Bacterial Infection	Infections	1104 (1099, 1109)	913 (908, 917)	1008 (1005, 1012)
Chronic Hepatitis	Infections	19 (19, 20)	29 (28, 30)	24 (24, 25)
Encephalitis	Infections	3 (2, 3)	3 (2, 3)	3 (2, 3)
Fungal Infection	Infections	87 (85, 88)	63 (62, 64)	75 (74, 76)
HIV	Infections	6 (6, 7)	11 (11, 12)	9 (9, 9)
Infection – Anorectal	Infections	27 (26, 27)	45 (44, 45)	36 (35, 36)
Infection – Bone	Infections	21 (20, 22)	37 (36, 38)	29 (28, 30)
Infection – Digestive System	Infections	265 (263, 268)	235 (233, 237)	250 (249, 252)
Infection – Ear/Upper Respiratory Tract	Infections	371 (369, 374)	392 (389, 394)	382 (380, 383)
Infection – Eye	Infections	25 (25, 26)	29 (28, 30)	27 (27, 28)
Infection – Heart	Infections	3 (3, 4)	8 (7, 8)	6 (5, 6)
Infection – Liver	Infections	16 (15, 16)	26 (25, 27)	21 (20, 21)

Infection – Lower Respiratory Tract	Infections	581 (578, 585)	666 (663, 670)	624 (621, 626)
Infection – Male Genitourinary	Infections	--	64 (63, 65)	64 (63, 65)
Infection – Other Genitourianry	Infections	45 (44, 46)	1 (1, 2)	23 (23, 24)
Infection – Other nervous system	Infections	11 (10, 11)	12 (11, 12)	11 (11, 11)
Infection – Other organisms	Infections	917 (913, 921)	976 (971, 980)	946 (943, 949)
Infection – Other organs	Infections	341 (339, 344)	319 (317, 322)	330 (328, 332)
Infection – Skin	Infections	268 (266, 271)	322 (319, 325)	295 (293, 297)
Meningitis	Infections	12 (11, 12)	11 (10, 11)	11 (11, 12)
Parasitic Infection	Infections	13 (12, 13)	12 (11, 12)	12 (12, 13)
Pelvic Inflammatory Disease	Infections	183 (181, 185)	--	183 (181, 185)
Rheumatic Fever	Infections	42 (41, 43)	33 (32, 34)	37 (37, 38)
Septicaemia	Infections	114 (113, 116)	139 (138, 141)	127 (126, 128)
Tuberculosis	Infections	70 (68, 71)	66 (65, 67)	68 (67, 69)
Urinary Tract Infection	Infections	526 (523, 529)	353 (350, 356)	439 (437, 441)
Viral Infection	Infections	332 (330, 335)	322 (319, 324)	327 (325, 329)
Ankylosing Spondylosis	Musculoskeletal	11 (11, 12)	27 (26, 27)	19 (19, 19)
Carpal Tunnel Syndrome	Musculoskeletal	504 (501, 507)	236 (234, 239)	370 (368, 372)
Collapsed Vertebra	Musculoskeletal	36 (35, 37)	23 (22, 23)	29 (29, 30)
Enteropathic Arthropathy	Musculoskeletal	1 (1, 2)	1 (1, 1)	1 (1, 1)
Enthesopathy	Musculoskeletal	1871 (1864, 1877)	1837 (1830, 1843)	1854 (1849, 1858)
Fibromatosis	Musculoskeletal	70 (69, 71)	154 (152, 156)	112 (111, 113)
Fracture – Hip	Musculoskeletal	157 (155, 158)	89 (88, 91)	123 (122, 124)
Fracture – Wrist	Musculoskeletal	363 (360, 365)	277 (275, 280)	320 (318, 322)
Giant Cell Arteritis	Musculoskeletal	24 (24, 25)	12 (11, 12)	18 (18, 18)
Gout	Musculoskeletal	133 (131, 135)	504 (500, 507)	318 (316, 320)
Intervertebral Disc Disorder	Musculoskeletal	314 (312, 317)	316 (313, 319)	315 (313, 317)
Juvenile Arthritis	Musculoskeletal	8 (7, 8)	5 (4, 5)	6 (6, 7)
Lupus Erythematosus	Musculoskeletal	26 (25, 27)	6 (6, 6)	16 (16, 16)
Osteoarthritis	Musculoskeletal	1445 (1439, 1450)	1099 (1093, 1104)	1272 (1268, 1275)
Osteoporosis	Musculoskeletal	494 (490, 497)	113 (111, 115)	303 (302, 305)
Polymyalgia Rheumatica	Musculoskeletal	115 (113, 117)	66 (64, 67)	90 (89, 91)
Psoriatic Arthritis	Musculoskeletal	23 (23, 24)	24 (23, 25)	24 (23, 24)
Reactive Arthritis	Musculoskeletal	9 (8, 9)	12 (12, 13)	11 (10, 11)
Rheumatoid Arthritis	Musculoskeletal	156 (154, 158)	75 (74, 76)	115 (114, 117)
Scleroderma	Musculoskeletal	7 (7, 7)	2 (2, 2)	4 (4, 5)
Scoliosis	Musculoskeletal	103 (102, 104)	52 (51, 54)	78 (77, 79)
Sjogren Syndrome	Musculoskeletal	19 (18, 19)	3 (2, 3)	11 (10, 11)
Spinal Stenosis	Musculoskeletal	79 (77, 80)	82 (80, 83)	80 (79, 81)
Spondylolisthesis	Musculoskeletal	42 (41, 43)	30 (29, 31)	36 (35, 36)
Spondylosis	Musculoskeletal	514 (510, 517)	402 (399, 405)	458 (456, 460)
Autonomic Neuropathy	Neurological	27 (26, 28)	24 (23, 25)	26 (25, 26)
Bell's Palsy	Neurological	75 (74, 77)	76 (74, 77)	76 (75, 76)
Cerebral Palsy	Neurological	14 (13, 14)	18 (17, 18)	16 (15, 16)
Chronic Fatigue Syndrome	Neurological	218 (216, 220)	78 (76, 79)	148 (146, 149)
Diabetic Neuropathy	Neurological	30 (30, 31)	52 (51, 54)	41 (41, 42)
Epilepsy	Neurological	174 (172, 176)	184 (182, 186)	179 (178, 181)
Essential Tremor	Neurological	24 (23, 25)	27 (26, 28)	26 (25, 26)
Idiopathic Intracranial Hypertension	Neurological	8 (8, 8)	2 (2, 2)	5 (5, 5)
Migraine	Neurological	1008 (1004, 1013)	385 (382, 388)	697 (694, 699)
Motor Neurone Disease	Neurological	3 (3, 4)	6 (5, 6)	5 (4, 5)
Multiple Sclerosis	Neurological	36 (35, 37)	16 (15, 17)	26 (26, 27)
Myasthenia Gravis	Neurological	4 (4, 5)	4 (4, 5)	4 (4, 4)
Parkinson's Disease	Neurological	35 (34, 36)	59 (58, 60)	47 (46, 48)
Peripheral Neuropathy	Neurological	205 (203, 207)	231 (229, 233)	218 (216, 220)
Trigeminal Neuralgia	Neurological	73 (72, 74)	37 (36, 38)	55 (54, 56)
Congenital Septal Defect	Perinatal	39 (38, 40)	34 (34, 35)	37 (36, 37)
Down Syndrome	Perinatal	7 (7, 7)	7 (7, 7)	7 (7, 7)
High Birth Weight	Perinatal	7 (7, 7)	13 (13, 13)	10 (10, 10)
Intrauterine Hypoxia	Perinatal	43 (43, 44)	50 (49, 51)	47 (46, 47)
Low Birth Weight	Perinatal	58 (57, 59)	54 (53, 55)	56 (56, 57)
Neonatal Jaundice	Perinatal	80 (79, 81)	104 (102, 105)	92 (91, 93)
Patent Ductus Arteriosus	Perinatal	11 (11, 12)	10 (9, 10)	10 (10, 11)
Post-term Delivery	Perinatal	8 (8, 9)	9 (8, 9)	8 (8, 9)
Premature Delivery	Perinatal	63 (62, 64)	76 (75, 77)	69 (69, 70)
Respiratory Distress of the Newborn	Perinatal	33 (32, 34)	51 (50, 52)	42 (41, 43)
Sepsis of the Newborn	Perinatal	13 (13, 14)	18 (18, 19)	16 (15, 16)
Spina Bifida	Perinatal	13 (12, 13)	11 (10, 11)	12 (11, 12)
Alcohol Misuse	Psychiatric	189 (187, 191)	398 (395, 400)	293 (292, 295)
Anxiety	Psychiatric	1662 (1657, 1668)	930 (926, 934)	1296 (1293, 1300)
Autism	Psychiatric	16 (16, 17)	62 (61, 63)	39 (38, 40)

Bipolar Affective Disorder	Psychiatric	53 (52, 54)	37 (36, 38)	45 (44, 46)
Delirium	Psychiatric	57 (56, 58)	59 (58, 60)	58 (57, 59)
Dementia	Psychiatric	230 (228, 232)	187 (185, 189)	209 (207, 210)
Depression	Psychiatric	2209 (2203, 2216)	1279 (1274, 1284)	1744 (1740, 1749)
Eating Disorders	Psychiatric	63 (62, 64)	4 (4, 4)	33 (33, 34)
Hyperkinetic Disorders	Psychiatric	13 (12, 13)	56 (55, 57)	34 (34, 35)
Intellectual Disability	Psychiatric	53 (52, 54)	76 (75, 78)	64 (64, 65)
Obsessive Compulsive Disorder	Psychiatric	51 (50, 52)	36 (36, 37)	44 (43, 44)
Personality Disorder	Psychiatric	63 (62, 64)	63 (62, 64)	63 (62, 64)
Schizophrenia	Psychiatric	67 (66, 68)	84 (82, 85)	75 (75, 76)
Substance Misuse	Psychiatric	114 (112, 115)	205 (203, 207)	159 (158, 161)
Allergic/chronic Rhinitis	Respiratory	1537 (1531, 1542)	1449 (1444, 1455)	1493 (1489, 1497)
Asbestosis	Respiratory	1 (1, 1)	23 (23, 24)	12 (12, 13)
Aspiration Pneumonitis	Respiratory	32 (31, 32)	51 (50, 52)	41 (41, 42)
Asthma	Respiratory	1565 (1560, 1571)	1432 (1427, 1438)	1499 (1495, 1503)
Bronchiectasis	Respiratory	59 (58, 61)	55 (54, 56)	57 (56, 58)
Chronic Obstructive Pulmonary Disease	Respiratory	332 (329, 334)	411 (408, 415)	371 (369, 374)
Chronic Sinusitis	Respiratory	272 (270, 275)	167 (165, 169)	219 (218, 221)
Hypertrophic Nasal Turbinates	Respiratory	28 (27, 29)	44 (43, 45)	36 (36, 37)
Nasal Polyps	Respiratory	78 (77, 79)	139 (137, 141)	109 (107, 110)
Pleural Effusion	Respiratory	144 (142, 145)	185 (183, 187)	164 (163, 166)
Pleural Plaque	Respiratory	4 (4, 4)	40 (39, 41)	22 (21, 22)
Pneumothorax	Respiratory	28 (27, 29)	69 (67, 70)	48 (48, 49)
Pulmonary Collapse	Respiratory	51 (50, 52)	61 (60, 62)	56 (55, 57)
Pulmonary Fibrosis	Respiratory	21 (20, 22)	36 (35, 37)	28 (28, 29)
Respiratory Failure	Respiratory	75 (74, 76)	89 (87, 90)	82 (81, 83)
Sleep apnoea	Respiratory	55 (54, 56)	146 (144, 148)	101 (100, 102)
Acne	Skin	858 (854, 862)	611 (608, 615)	734 (732, 737)
Actinic keratosis	Skin	215 (212, 217)	332 (329, 335)	273 (272, 275)
Alopecia Areata	Skin	43 (42, 43)	33 (33, 34)	38 (37, 39)
Dermatitis	Skin	2779 (2771, 2786)	2308 (2301, 2315)	2543 (2538, 2548)
Hidradenitis	Skin	40 (39, 41)	15 (14, 15)	27 (27, 28)
Lichen Planus	Skin	71 (70, 72)	52 (51, 53)	62 (61, 62)
Pilonidal cyst/sinus	Skin	55 (54, 56)	120 (119, 122)	88 (87, 89)
Psoriasis	Skin	353 (350, 356)	343 (341, 346)	348 (346, 350)
Rosacea	Skin	297 (294, 299)	203 (201, 206)	250 (249, 252)
Seborrheic Dermatitis	Skin	516 (513, 519)	481 (478, 484)	498 (496, 501)
Urticaria	Skin	637 (633, 640)	403 (401, 406)	520 (518, 522)
Vitiligo	Skin	39 (38, 39)	33 (32, 34)	36 (35, 36)

--Small numbers: to protect patient confidentiality, where prevalence counts were <100, period prevalence estimates have been replaced with “--”.

Supplementary Table S6. Age and sex-standardised period prevalence per 10000 (95% confidence intervals) from 1 April 2010 to 31 Mar 2015 for 308 medical conditions, stratified by ethnicity.

Condition	Category	Asian	Black	White
Benign Neoplasm – Brain	Benign Neo	31 (28, 35)	33 (28, 38)	29 (28, 30)
Benign Neoplasm – Colon	Benign Neo	246 (234, 257)	234 (219, 250)	346 (344, 349)
Benign Neoplasm – Ovary	Benign Neo	286 (273, 298)	335 (319, 351)	444 (440, 447)
Benign Neoplasm – Stomach	Benign Neo	58 (52, 64)	56 (48, 63)	66 (65, 67)
Benign Neoplasm – Uterus	Benign Neo	196 (183, 208)	205 (190, 219)	208 (205, 210)
Cervical Intra-epithelial Neoplasia	Benign Neo	74 (68, 80)	181 (169, 192)	284 (281, 287)
Haemangioma	Benign Neo	74 (70, 79)	37 (33, 41)	146 (144, 147)
Leiomyoma	Benign Neo	473 (455, 491)	1210 (1178, 1242)	409 (405, 412)
Hodgkins Lymphoma	Cancers	--	--	9 (8, 9)
Leukaemia	Cancers	17 (15, 20)	--	26 (26, 27)
Monoclonal Gammopathy of Undetermined Significance	Cancers	--	35 (29, 42)	14 (13, 14)
Myelodysplastic Syndrome	Cancers	--	--	13 (12, 13)
Non Hodgkins Lymphoma	Cancers	30 (26, 34)	28 (23, 32)	35 (34, 36)
Plasma Cell Malignancy	Cancers	--	--	12 (11, 12)
Polycythaemia vera	Cancers	--	--	14 (13, 14)
Primary Malignancy – Biliary	Cancers	--	--	5 (4, 5)
Primary Malignancy – Bladder	Cancers	19 (15, 22)	--	54 (53, 55)
Primary Malignancy – Bone	Cancers	--	--	5 (4, 5)
Primary Malignancy – Bowel	Cancers	44 (39, 50)	60 (52, 69)	92 (91, 94)
Primary Malignancy – Brain	Cancers	--	--	12 (12, 12)
Primary Malignancy – Breast	Cancers	98 (91, 106)	92 (84, 101)	159 (158, 161)
Primary Malignancy – Cervix	Cancers	--	--	22 (22, 23)
Primary Malignancy – Kidney	Cancers	--	--	21 (20, 21)
Primary Malignancy – Liver	Cancers	--	--	5 (5, 5)
Primary Malignancy – Lung	Cancers	21 (17, 25)	--	53 (52, 54)
Primary Malignancy – Melanoma	Cancers	--	--	61 (60, 62)
Primary Malignancy – Mesothelioma	Cancers	--	--	4 (3, 4)
Primary Malignancy – Multiple Sites	Cancers	--	--	2 (2, 2)
Primary Malignancy – Oesophageal	Cancers	--	--	15 (15, 16)
Primary Malignancy – Oropharyngeal	Cancers	--	--	20 (20, 21)
Primary Malignancy – other	Cancers	24 (21, 28)	28 (23, 32)	39 (38, 40)
Primary Malignancy – Ovary	Cancers	--	--	33 (32, 34)
Primary Malignancy – Pancreas	Cancers	--	--	11 (11, 12)
Primary Malignancy – Prostate	Cancers	111 (97, 125)	418 (383, 453)	233 (230, 236)
Primary Malignancy – Skin	Cancers	20 (17, 24)	--	351 (349, 353)
Primary Malignancy – Stomach	Cancers	--	--	13 (12, 13)
Primary Malignancy – Testis	Cancers	--	--	22 (21, 23)
Primary Malignancy – Thyroid	Cancers	--	--	8 (7, 8)
Primary Malignancy – Uterus	Cancers	35 (28, 42)	--	42 (41, 43)
Secondary Malignancy – Adrenal	Cancers	--	--	6 (5, 6)
Secondary Malignancy – Bone	Cancers	19 (16, 23)	45 (37, 52)	42 (41, 43)
Secondary Malignancy – Bowel	Cancers	--	--	4 (3, 4)
Secondary Malignancy – Brain	Cancers	--	--	14 (13, 14)
Secondary Malignancy – Liver	Cancers	21 (17, 24)	41 (34, 47)	47 (46, 48)
Secondary Malignancy – Lung	Cancers	15 (12, 18)	33 (27, 39)	33 (32, 33)
Secondary Malignancy – Lymph Nodes	Cancers	38 (33, 42)	44 (38, 50)	79 (78, 80)
Secondary Malignancy – other	Cancers	--	--	21 (20, 21)
Secondary Malignancy – Peritoneum	Cancers	--	--	18 (17, 18)
Secondary Malignancy – Pleura	Cancers	--	--	8 (8, 9)
Abdominal Aortic Aneurysm	Cardiovascular	--	--	62 (61, 63)
Atrial Fibrillation	Cardiovascular	262 (248, 276)	236 (219, 254)	489 (486, 492)
Atrioventricular Block, first degree	Cardiovascular	35 (30, 41)	--	39 (39, 40)
Atrioventricular Block, second degree	Cardiovascular	--	--	15 (15, 15)
Atrioventricular Block, third degree	Cardiovascular	22 (18, 26)	--	23 (22, 23)
Bifascicular Block	Cardiovascular	--	--	4 (4, 4)
Cardiomyopathy – other	Cardiovascular	32 (28, 37)	45 (39, 51)	31 (30, 32)
Coronary Heart Disease (not otherwise specified)	Cardiovascular	153 (142, 163)	109 (98, 121)	118 (117, 120)
Dilated cardiomyopathy	Cardiovascular	--	25 (21, 29)	18 (17, 18)
Heart Failure	Cardiovascular	359 (342, 376)	274 (256, 293)	309 (307, 311)
Hypertension	Cardiovascular	2546 (2508, 2584)	2871 (2823, 2920)	2185 (2179, 2191)
Hypertrophic cardiomyopathy	Cardiovascular	--	--	6 (6, 7)
Intracerebral Haemorrhage	Cardiovascular	35 (30, 40)	50 (43, 57)	35 (35, 36)
Ischaemic Stroke	Cardiovascular	150 (140, 161)	169 (155, 183)	150 (149, 152)
Left Bundle Branch Block	Cardiovascular	68 (61, 75)	46 (38, 54)	62 (61, 63)
Multiple valve disorder	Cardiovascular	68 (61, 75)	54 (46, 62)	65 (64, 66)

Myocardial Infarction	Cardiovascular	478 (460, 497)	212 (196, 228)	341 (339, 343)
Non-rheumatic Aortic valve disorder	Cardiovascular	94 (86, 102)	73 (64, 82)	111 (109, 112)
Non-rheumatic Mitral valve disorder	Cardiovascular	91 (83, 98)	63 (56, 71)	94 (93, 95)
Pericardial Effusion	Cardiovascular	21 (18, 24)	29 (24, 33)	19 (19, 20)
Peripheral Arterial Disease	Cardiovascular	147 (136, 157)	144 (130, 158)	184 (183, 186)
Primary Pulmonary Hypertension	Cardiovascular	25 (21, 29)	36 (30, 41)	21 (21, 22)
Pulmonary Embolism	Cardiovascular	45 (40, 50)	100 (91, 109)	118 (117, 119)
Raynauds Disease	Cardiovascular	88 (82, 94)	57 (52, 63)	114 (113, 116)
Rheumatic Valve Disorder	Cardiovascular	26 (23, 30)	25 (21, 30)	24 (24, 25)
Right Bundle Branch Block	Cardiovascular	73 (66, 80)	53 (45, 61)	62 (61, 63)
Secondary Pulmonary Hypertension	Cardiovascular	--	--	13 (12, 13)
Sick Sinus Syndrome	Cardiovascular	--	--	14 (14, 15)
Stable Angina	Cardiovascular	704 (682, 726)	321 (301, 340)	511 (508, 514)
Stroke – not otherwise specified	Cardiovascular	108 (99, 117)	134 (121, 147)	123 (121, 124)
Subarachnoid Haemorrhage	Cardiovascular	14 (11, 16)	23 (19, 27)	25 (24, 25)
Subdural haematoma	Cardiovascular	--	--	13 (13, 14)
Supraventricular Tachycardia	Cardiovascular	65 (60, 71)	49 (43, 55)	89 (88, 90)
Transient Ischaemic Attack	Cardiovascular	168 (157, 179)	158 (144, 172)	212 (210, 213)
Trifascicular Block	Cardiovascular	--	--	5 (5, 5)
Unstable Angina	Cardiovascular	310 (296, 325)	140 (127, 153)	182 (181, 184)
Venous thrombolism (Excl PE)	Cardiovascular	83 (76, 89)	131 (121, 141)	181 (179, 182)
Ventricular Tachycardia	Cardiovascular	19 (16, 22)	--	24 (24, 25)
Abdominal Hernia	Digestive	501 (486, 516)	645 (627, 663)	869 (865, 873)
Alcoholic Liver Disease	Digestive	23 (19, 26)	18 (14, 21)	44 (43, 45)
Anal Fissure	Digestive	330 (320, 340)	174 (165, 183)	264 (262, 266)
Angiodysplasia of colon	Digestive	--	--	10 (9, 10)
Anorectal Fistula	Digestive	73 (68, 78)	47 (42, 51)	53 (52, 54)
Anorectal Prolapse	Digestive	26 (23, 30)	25 (21, 29)	38 (37, 38)
Appendicitis	Digestive	260 (251, 268)	278 (266, 290)	658 (655, 661)
Autoimmune liver disease	Digestive	--	--	8 (8, 9)
Barrett's Oesophagus	Digestive	31 (26, 35)	--	65 (64, 66)
Cholangitis	Digestive	16 (13, 19)	--	22 (21, 23)
Cholecystitis	Digestive	146 (138, 154)	96 (89, 104)	207 (206, 209)
Cholelithiasis	Digestive	306 (294, 318)	198 (187, 209)	404 (402, 406)
Cirrhosis	Digestive	36 (32, 40)	34 (28, 39)	43 (42, 44)
Coeliac Disease	Digestive	30 (27, 33)	--	42 (41, 42)
Crohns Disease	Digestive	35 (32, 38)	18 (15, 22)	53 (52, 54)
Diaphragmatic Hernia	Digestive	466 (450, 482)	316 (299, 332)	588 (585, 591)
Diverticular Disease	Digestive	220 (208, 233)	275 (258, 293)	539 (536, 541)
Fatty Liver	Digestive	54 (49, 58)	30 (25, 34)	49 (48, 50)
Gastritis	Digestive	929 (909, 949)	714 (692, 735)	748 (745, 751)
Gastro-oesophageal Reflux Disease	Digestive	1134 (1113, 1154)	843 (821, 865)	1079 (1075, 1083)
Irritable Bowel Syndrome	Digestive	408 (396, 419)	300 (288, 312)	703 (699, 706)
Liver Failure	Digestive	18 (15, 21)	--	18 (17, 18)
Oesophageal Ulcer	Digestive	651 (634, 668)	423 (406, 440)	684 (681, 687)
Oesophageal Varices	Digestive	15 (13, 18)	--	17 (16, 17)
Pancreatitis	Digestive	71 (65, 77)	48 (42, 54)	73 (72, 74)
Peptic Ulcer	Digestive	310 (297, 324)	291 (275, 306)	277 (275, 279)
Peritonitis	Digestive	44 (40, 48)	56 (50, 62)	80 (79, 81)
Portal Hypertension	Digestive	14 (11, 16)	--	16 (15, 16)
Ulcerative Colitis	Digestive	76 (71, 81)	31 (26, 35)	81 (80, 82)
Volvulus	Digestive	--	--	14 (14, 15)
Deafness	Ear	751 (732, 769)	443 (425, 460)	977 (974, 981)
Meniere's Disease	Ear	24 (21, 28)	--	41 (40, 41)
Tinnitus	Ear	356 (343, 369)	276 (261, 290)	344 (341, 346)
Cystic Fibrosis	Endocrine	--	--	6 (5, 6)
Diabetes Mellitus – other or not specified	Endocrine	70 (64, 75)	66 (59, 72)	37 (36, 37)
Hyperparathyroidism	Endocrine	52 (46, 57)	61 (54, 69)	23 (23, 24)
Low HDL-C	Endocrine	1444 (1419, 1470)	730 (708, 753)	716 (712, 719)
Obesity	Endocrine	1512 (1489, 1536)	2284 (2249, 2319)	1865 (1859, 1870)
Polycystic Ovarian Syndrome	Endocrine	130 (123, 137)	77 (70, 83)	100 (98, 101)
Raised LDL-C	Endocrine	2075 (2044, 2105)	1953 (1915, 1990)	1583 (1578, 1588)
Raised Total Cholesterol	Endocrine	2681 (2647, 2716)	2383 (2341, 2425)	2528 (2522, 2534)
Raised Triglycerides	Endocrine	1845 (1816, 1874)	637 (615, 660)	1212 (1207, 1216)
Syndrome of Inappropriate AntiDiuretic Hormone	Endocrine	--	--	6 (6, 7)
Thyroid Disease	Endocrine	669 (653, 685)	307 (293, 321)	555 (552, 558)
Type 1 Diabetes Mellitus	Endocrine	28 (25, 32)	37 (33, 42)	56 (55, 57)
Type 2 Diabetes Mellitus	Endocrine	1622 (1592, 1652)	1268 (1234, 1303)	612 (609, 615)
Anterior Uveitis	Eye	115 (108, 123)	134 (124, 144)	76 (75, 77)

Blindness	Eye	153 (144, 162)	194 (180, 208)	131 (130, 132)
Cataract	Eye	1311 (1279, 1342)	1105 (1066, 1143)	711 (708, 714)
Diabetic Eye Disease	Eye	580 (561, 598)	436 (415, 457)	213 (211, 215)
Glaucoma	Eye	255 (242, 268)	430 (408, 451)	190 (188, 191)
Keratitis	Eye	60 (56, 65)	37 (32, 41)	55 (55, 56)
Macular Degeneration	Eye	186 (175, 198)	113 (101, 124)	153 (152, 155)
Posterior Uveitis	Eye	--	--	6 (6, 6)
Ptosis	Eye	36 (33, 40)	40 (35, 45)	44 (43, 45)
Reitnal Detachment	Eye	65 (59, 70)	62 (55, 69)	66 (65, 67)
Retinal Vascular Occlusion	Eye	57 (51, 63)	46 (38, 53)	45 (44, 46)
Scleritis	Eye	60 (55, 64)	45 (40, 50)	48 (47, 48)
Acute Kidney Injury	Genitourinary	250 (236, 263)	263 (246, 280)	225 (223, 227)
Benign Prostatic Hyperplasia	Genitourinary	827 (792, 863)	871 (822, 920)	842 (836, 847)
Chronic Cystitis	Genitourinary	14 (11, 16)	--	24 (24, 25)
Chronic Kidney Disease	Genitourinary	561 (539, 582)	494 (469, 520)	462 (459, 464)
Dysmenorrhoea	Genitourinary	503 (488, 518)	612 (592, 632)	657 (653, 662)
End Stage Renal Disease	Genitourinary	62 (56, 68)	80 (72, 89)	32 (32, 33)
Endometrial Hyperplasia	Genitourinary	70 (63, 78)	71 (63, 79)	83 (82, 85)
Endometriosis	Genitourinary	177 (168, 187)	148 (138, 158)	268 (265, 271)
Erectile Dysfunction	Genitourinary	1147 (1115, 1180)	1471 (1421, 1520)	1005 (999, 1010)
Female Infertility	Genitourinary	327 (315, 339)	314 (300, 329)	265 (262, 267)
Glomerulonephritis	Genitourinary	83 (77, 89)	82 (74, 90)	78 (77, 79)
Hydrocele	Genitourinary	105 (97, 113)	146 (133, 160)	241 (238, 243)
Male infertility	Genitourinary	83 (76, 89)	85 (77, 94)	65 (64, 67)
Menorrhagia	Genitourinary	1268 (1241, 1295)	1317 (1286, 1349)	1520 (1513, 1526)
Neuropahtic Bladder	Genitourinary	117 (110, 125)	101 (92, 109)	112 (111, 114)
Obstructive and reflux uropathy	Genitourinary	77 (72, 82)	65 (58, 71)	104 (103, 105)
Postcoital Bleeding	Genitourinary	116 (109, 123)	148 (137, 158)	300 (297, 303)
Postmenopausal Bleeding	Genitourinary	369 (349, 390)	359 (334, 384)	434 (431, 438)
Tubulo-interstitial Nephropathy	Genitourinary	49 (45, 53)	35 (32, 39)	38 (38, 39)
Undescended Testis	Genitourinary	56 (52, 60)	57 (52, 63)	106 (104, 108)
Urinary Incontinence	Genitourinary	468 (454, 483)	369 (353, 385)	446 (444, 449)
Urolithiasis	Genitourinary	258 (248, 267)	150 (140, 160)	278 (276, 281)
Uterovaginal Prolapse	Genitourinary	400 (380, 420)	289 (269, 310)	603 (599, 608)
Agranulocytosis	Haem/Imm	51 (47, 55)	146 (137, 155)	68 (67, 69)
Anaemia – other	Haem/Imm	920 (901, 939)	800 (778, 821)	546 (543, 549)
Aplastic Anaemia	Haem/Imm	15 (12, 18)	23 (19, 27)	14 (13, 14)
Folate Deficiency Anaemia	Haem/Imm	24 (20, 27)	--	18 (17, 18)
Hypersplenism	Haem/Imm	12 (10, 14)	12 (10, 14)	18 (18, 19)
Hyposplenism	Haem/Imm	--	--	18 (17, 18)
Immunodeficiency	Haem/Imm	--	--	8 (7, 8)
Iron Deficiency Anaemia	Haem/Imm	1050 (1031, 1069)	631 (613, 649)	448 (445, 450)
Other haemolytic anaemia	Haem/Imm	21 (19, 23)	38 (34, 42)	12 (11, 12)
Primary thrombocytopaenia	Haem/Imm	19 (16, 21)	18 (15, 21)	18 (17, 18)
Sarcoidosis	Haem/Imm	37 (33, 41)	58 (52, 64)	21 (20, 21)
Secondary Polycythaemia	Haem/Imm	--	--	12 (11, 12)
Secondary Thrombocytopaenia	Haem/Imm	53 (48, 57)	83 (75, 90)	50 (49, 51)
Sickle Cell Disease	Haem/Imm	--	61 (56, 65)	0 (0, 1)
Sickle Cell Trait	Haem/Imm	10 (9, 12)	382 (370, 395)	1 (1, 2)
Thalassaemia	Haem/Imm	34 (31, 37)	33 (29, 36)	2 (2, 3)
Thalassaemia Trait	Haem/Imm	124 (119, 130)	79 (73, 85)	5 (5, 6)
Thrombophilia	Haem/Imm	7 (6, 9)	--	14 (14, 15)
Vitamin B12 deficiency anaemia	Haem/Imm	116 (108, 123)	28 (23, 32)	55 (54, 56)
Bacterial Infection	Infections	1118 (1098, 1138)	1058 (1033, 1082)	1209 (1205, 1213)
Chronic Hepatitis	Infections	45 (41, 48)	82 (76, 88)	25 (25, 26)
Encephalitis	Infections	--	--	3 (3, 3)
Fungal Infection	Infections	78 (73, 84)	89 (82, 96)	90 (88, 91)
HIV	Infections	--	107 (101, 113)	7 (6, 7)
Infection – Anorectal	Infections	44 (40, 47)	36 (32, 40)	46 (45, 47)
Infection – Bone	Infections	35 (31, 39)	45 (40, 50)	35 (35, 36)
Infection – Digestive System	Infections	332 (322, 343)	252 (240, 263)	300 (298, 303)
Infection – Ear/Upper Respiratory Tract	Infections	331 (323, 339)	258 (249, 267)	502 (499, 505)
Infection – Eye	Infections	25 (23, 27)	22 (19, 24)	33 (32, 33)
Infection – Heart	Infections	--	--	7 (7, 7)
Infection – Liver	Infections	42 (38, 46)	60 (55, 66)	23 (23, 24)
Infection – Lower Respiratory Tract	Infections	669 (653, 686)	573 (555, 591)	733 (730, 737)
Infection – Male Genitourinary	Infections	48 (43, 54)	80 (70, 90)	87 (85, 89)
Infection – Other Genitourianry	Infections	20 (18, 22)	26 (23, 29)	28 (28, 29)
Infection – Other nervous system	Infections	16 (13, 18)	16 (13, 19)	13 (13, 14)
Infection – Other organisms	Infections	929 (912, 946)	762 (743, 780)	1166 (1162, 1170)

Infection – Other organs	Infections	387 (377, 397)	419 (406, 432)	400 (397, 402)
Infection – Skin	Infections	281 (271, 290)	239 (229, 250)	366 (364, 369)
Meningitis	Infections	11 (9, 13)	10 (8, 12)	15 (14, 15)
Parasitic Infection	Infections	17 (15, 20)	76 (70, 82)	13 (13, 14)
Pelvic Inflammatory Disease	Infections	141 (132, 150)	203 (190, 215)	222 (220, 225)
Rheumatic Fever	Infections	29 (25, 33)	33 (27, 38)	41 (40, 41)
Septicaemia	Infections	146 (139, 154)	143 (134, 152)	147 (146, 149)
Tuberculosis	Infections	371 (359, 383)	169 (160, 178)	55 (54, 55)
Urinary Tract Infection	Infections	474 (459, 489)	434 (416, 453)	507 (505, 510)
Viral Infection	Infections	317 (309, 325)	272 (263, 281)	409 (406, 411)
Ankylosing Spondylosis	Musculoskeletal	13 (11, 15)	--	23 (22, 23)
Carpal Tunnel Syndrome	Musculoskeletal	330 (319, 342)	313 (299, 328)	420 (418, 423)
Collapsed Vertebra	Musculoskeletal	24 (20, 28)	--	33 (33, 34)
Enteropathic Arthropathy	Musculoskeletal	--	--	1 (1, 2)
Enthesopathy	Musculoskeletal	1783 (1755, 1810)	1467 (1437, 1498)	2029 (2023, 2034)
Fibromatosis	Musculoskeletal	38 (33, 43)	33 (27, 39)	127 (126, 129)
Fracture – Hip	Musculoskeletal	72 (64, 79)	35 (29, 41)	138 (137, 140)
Fracture – Wrist	Musculoskeletal	125 (118, 132)	72 (66, 77)	382 (380, 384)
Giant Cell Arteritis	Musculoskeletal	24 (20, 28)	--	20 (19, 20)
Gout	Musculoskeletal	292 (279, 304)	309 (291, 327)	345 (343, 347)
Intervertebral Disc Disorder	Musculoskeletal	285 (274, 297)	204 (192, 216)	372 (369, 374)
Juvenile Arthritis	Musculoskeletal	--	--	8 (8, 8)
Lupus Erythematosus	Musculoskeletal	25 (22, 28)	34 (30, 38)	17 (17, 18)
Osteoarthritis	Musculoskeletal	1374 (1344, 1404)	1192 (1158, 1227)	1417 (1412, 1422)
Osteoporosis	Musculoskeletal	356 (340, 371)	154 (142, 167)	333 (331, 335)
Polymyalgia Rheumatica	Musculoskeletal	64 (58, 71)	39 (32, 46)	97 (96, 99)
Psoriatic Arthritis	Musculoskeletal	19 (16, 22)	--	28 (27, 29)
Reactive Arthritis	Musculoskeletal	9 (7, 11)	--	12 (12, 13)
Rheumatoid Arthritis	Musculoskeletal	140 (132, 149)	91 (83, 100)	132 (130, 133)
Scleroderma	Musculoskeletal	--	--	5 (5, 5)
Scoliosis	Musculoskeletal	61 (56, 66)	54 (48, 59)	88 (87, 90)
Sjogren Syndrome	Musculoskeletal	19 (16, 23)	--	12 (11, 12)
Spinal Stenosis	Musculoskeletal	98 (91, 106)	104 (93, 115)	92 (91, 93)
Spondylolisthesis	Musculoskeletal	40 (35, 45)	41 (34, 48)	41 (40, 42)
Spondylosis	Musculoskeletal	489 (472, 506)	404 (383, 424)	509 (506, 512)
Autonomic Neuropathy	Neurological	25 (22, 29)	28 (23, 34)	31 (30, 31)
Bell's Palsy	Neurological	86 (80, 92)	73 (67, 79)	82 (81, 83)
Cerebral Palsy	Neurological	11 (10, 12)	12 (10, 14)	20 (20, 21)
Chronic Fatigue Syndrome	Neurological	109 (102, 115)	61 (55, 67)	171 (170, 173)
Diabetic Neuropathy	Neurological	83 (75, 90)	78 (69, 88)	47 (46, 48)
Epilepsy	Neurological	120 (114, 126)	137 (129, 145)	218 (216, 220)
Essential Tremor	Neurological	27 (24, 31)	--	28 (27, 29)
Idiopathic Intracranial Hypertension	Neurological	--	--	6 (6, 7)
Migraine	Neurological	516 (504, 528)	429 (416, 442)	788 (785, 791)
Motor Neurone Disease	Neurological	--	--	5 (5, 5)
Multiple Sclerosis	Neurological	9 (7, 10)	--	32 (31, 33)
Myasthenia Gravis	Neurological	--	--	5 (4, 5)
Parkinson's Disease	Neurological	50 (43, 56)	41 (33, 49)	51 (50, 52)
Peripheral Neuropathy	Neurological	245 (234, 257)	203 (190, 216)	253 (251, 255)
Trigeminal Neuralgia	Neurological	41 (36, 45)	37 (31, 42)	61 (60, 62)
Congenital Septal Defect	Perinatal	31 (28, 33)	23 (21, 26)	44 (43, 44)
Down Syndrome	Perinatal	5 (4, 7)	--	9 (8, 9)
High Birth Weight	Perinatal	4 (3, 4)	5 (4, 6)	12 (12, 13)
Intrauterine Hypoxia	Perinatal	36 (33, 38)	39 (36, 42)	56 (55, 57)
Low Birth Weight	Perinatal	75 (72, 78)	50 (47, 53)	63 (62, 64)
Neonatal Jaundice	Perinatal	90 (87, 94)	44 (41, 47)	106 (105, 107)
Patent Ductus Arteriosus	Perinatal	12 (10, 13)	10 (8, 11)	12 (11, 12)
Post-term Delivery	Perinatal	4 (3, 4)	--	10 (10, 11)
Premature Delivery	Perinatal	50 (47, 53)	56 (53, 60)	80 (79, 81)
Respiratory Distress of the Newborn	Perinatal	31 (29, 33)	38 (36, 41)	49 (49, 50)
Sepsis of the Newborn	Perinatal	17 (15, 18)	18 (16, 19)	18 (17, 18)
Spina Bifida	Perinatal	--	--	14 (14, 15)
Alcohol Misuse	Psychiatric	180 (173, 188)	162 (153, 171)	370 (367, 372)
Anxiety	Psychiatric	677 (662, 692)	587 (571, 604)	1496 (1491, 1501)
Autism	Psychiatric	20 (18, 22)	35 (32, 38)	46 (45, 47)
Bipolar Affective Disorder	Psychiatric	37 (33, 40)	47 (43, 52)	55 (55, 56)
Delirium	Psychiatric	44 (38, 50)	73 (63, 83)	64 (63, 65)
Dementia	Psychiatric	180 (167, 193)	278 (257, 300)	222 (220, 223)
Depression	Psychiatric	1098 (1079, 1118)	989 (967, 1011)	2031 (2025, 2036)
Eating Disorders	Psychiatric	11 (9, 12)	8 (7, 10)	40 (39, 41)
Hyperkinetic Disorders	Psychiatric	7 (6, 8)	15 (13, 17)	45 (44, 46)

Intellectual Disability	Psychiatric	43 (40, 46)	51 (47, 56)	80 (78, 81)
Obsessive Compulsive Disorder	Psychiatric	23 (20, 25)	--	51 (50, 52)
Personality Disorder	Psychiatric	19 (17, 22)	27 (23, 30)	81 (80, 82)
Schizophrenia	Psychiatric	93 (87, 98)	198 (188, 208)	88 (86, 89)
Substance Misuse	Psychiatric	78 (73, 82)	143 (135, 151)	208 (207, 210)
Allergic/chronic Rhinitis	Respiratory	1885 (1862, 1908)	1916 (1888, 1944)	1524 (1519, 1529)
Asbestosis	Respiratory	--	--	14 (13, 14)
Aspiration Pneumonitis	Respiratory	48 (43, 54)	49 (41, 56)	47 (46, 48)
Asthma	Respiratory	1428 (1408, 1449)	1172 (1150, 1195)	1675 (1670, 1680)
Bronchiectasis	Respiratory	70 (63, 76)	42 (36, 48)	65 (64, 66)
Chronic Obstructive Pulmonary Disease	Respiratory	252 (238, 266)	179 (165, 193)	430 (428, 433)
Chronic Sinusitis	Respiratory	158 (150, 165)	147 (138, 156)	250 (248, 252)
Hypertrophic Nasal Turbinates	Respiratory	49 (46, 52)	32 (29, 36)	46 (45, 47)
Nasal Polyps	Respiratory	108 (102, 114)	64 (58, 70)	123 (122, 124)
Pleural Effusion	Respiratory	178 (168, 188)	148 (137, 159)	189 (187, 191)
Pleural Plaque	Respiratory	--	--	25 (25, 26)
Pneumothorax	Respiratory	29 (25, 32)	22 (19, 25)	59 (59, 60)
Pulmonary Collapse	Respiratory	47 (42, 52)	51 (45, 58)	67 (66, 68)
Pulmonary Fibrosis	Respiratory	46 (40, 52)	--	32 (31, 32)
Respiratory Failure	Respiratory	99 (91, 107)	83 (74, 91)	95 (94, 96)
Sleep apnoea	Respiratory	110 (104, 116)	133 (125, 140)	122 (121, 123)
Acne	Skin	714 (702, 726)	587 (573, 601)	767 (763, 770)
Actinic keratosis	Skin	24 (20, 28)	--	299 (297, 301)
Alopecia Areata	Skin	80 (76, 85)	40 (36, 44)	36 (35, 37)
Dermatitis	Skin	2870 (2843, 2898)	2145 (2118, 2173)	2647 (2641, 2653)
Hidradenitis	Skin	24 (21, 26)	36 (33, 40)	31 (31, 32)
Lichen Planus	Skin	146 (138, 154)	51 (45, 56)	63 (62, 64)
Pilonidal cyst/sinus	Skin	82 (77, 86)	40 (36, 44)	106 (105, 108)
Psoriasis	Skin	270 (260, 279)	99 (92, 106)	388 (385, 390)
Rosacea	Skin	149 (143, 156)	55 (51, 60)	275 (273, 277)
Seborrheic Dermatitis	Skin	587 (575, 599)	359 (348, 370)	521 (519, 524)
Urticaria	Skin	633 (620, 647)	366 (354, 378)	551 (548, 554)
Vitiligo	Skin	135 (128, 142)	68 (62, 73)	31 (31, 32)

--Small numbers: to protect patient confidentiality, where prevalence counts were <100, period prevalence estimates have been replaced with "--".

Supplementary Table S7A. Median (interquartile range) age of first record for 308 medical conditions by sex.

Condition	Category	Both sexes	Female	Male
Benign Neo – Brain	Benign Neo	55 (41, 69)	55 (40, 70)	55 (42, 67)
Benign Neo – Colon	Benign Neo	63 (52, 72)	63 (52, 72)	63 (53, 71)
Benign Neo – Ovary	Benign Neo	36 (27, 47)	36 (27, 47)	NA (NA, NA)
Benign Neo – Stomach	Benign Neo	66 (56, 75)	66 (56, 75)	65 (55, 74)
Benign Neo – Uterus	Benign Neo	52 (44, 61)	52 (44, 61)	NA (NA, NA)
CIN	Benign Neo	31 (26, 39)	31 (26, 39)	NA (NA, NA)
Haemangioma	Benign Neo	34 (3, 53)	34 (2, 52)	35 (6, 55)
Leiomyoma	Benign Neo	46 (40, 51)	46 (40, 51)	NA (NA, NA)
Hodgkins Lymphoma	Cancers	34 (23, 53)	33 (23, 55)	35 (23, 52)
Leukaemia	Cancers	64 (47, 76)	65 (45, 77)	64 (49, 75)
MGUS	Cancers	72 (63, 80)	73 (63, 81)	72 (63, 79)
Myelodysplastic Syndrome	Cancers	76 (66, 83)	76 (65, 84)	76 (67, 83)
Non Hodgkins Lymphoma	Cancers	62 (48, 73)	64 (51, 75)	61 (47, 72)
Plasma Cell Ca	Cancers	70 (61, 79)	71 (62, 79)	69 (60, 78)
Polycythaemia vera	Cancers	58 (47, 69)	63 (51, 75)	56 (45, 66)
Pri Ca – Biliary	Cancers	72 (63, 81)	74 (64, 82)	71 (62, 79)
Pri Ca – Bladder	Cancers	71 (62, 78)	71 (62, 79)	70 (62, 78)
Pri Ca – Bone	Cancers	49 (25, 69)	52 (27, 70)	47 (24, 68)
Pri Ca – Bowel	Cancers	69 (60, 78)	70 (60, 79)	69 (60, 77)
Pri Ca – Brain	Cancers	52 (28, 68)	52 (29, 68)	52 (28, 67)
Pri Ca – Breast	Cancers	59 (50, 68)	59 (50, 68)	67 (57, 77)
Pri Ca – Cervix	Cancers	41 (34, 54)	41 (34, 54)	NA (NA, NA)
Pri Ca – Kidney	Cancers	67 (57, 76)	68 (57, 78)	67 (58, 75)
Pri Ca – Liver	Cancers	69 (60, 77)	70.5 (59.75, 78.25)	68 (60, 77)
Pri Ca – Lung	Cancers	71 (64, 79)	71 (63, 80)	72 (64, 79)
Pri Ca – Melanoma	Cancers	57 (42.75, 70)	54 (40, 68)	60 (47, 72)
Pri Ca – Mesothelioma	Cancers	72 (65, 79)	71 (64, 78)	73 (66, 80)
Pri Ca – Multiple Sites	Cancers	74 (66, 83)	72 (61, 80)	76 (68, 84)
Pri Ca – Oesophageal	Cancers	70 (62, 79)	74 (64, 83)	69 (61, 77)
Pri Ca – Oropharyngeal	Cancers	61 (51, 71)	62 (51, 73)	60 (52, 70)
Pri Ca – other	Cancers	65 (51, 76)	65 (51, 77)	64 (52, 75)
Pri Ca – Ovary	Cancers	61 (49, 71)	61 (49, 71)	NA (NA, NA)
Pri Ca – Pancreas	Cancers	72 (63, 81)	73 (64, 82)	71 (62, 79)
Pri Ca – Prostate	Cancers	71 (64, 77)	NA (NA, NA)	71 (64, 77)
Pri Ca – Skin	Cancers	68 (57, 77)	67 (56, 78)	68 (58, 76)
Pri Ca – Stomach	Cancers	72 (62, 79)	72 (62, 81)	71 (63, 79)
Pri Ca – Testis	Cancers	34 (28, 42)	NA (NA, NA)	34 (28, 42)
Pri Ca – Thyroid	Cancers	47 (35, 61)	45 (35, 59)	52 (40, 67)
Pri Ca – Uterus	Cancers	64 (57, 72)	64 (57, 72)	NA (NA, NA)
Sec Ca – Adrenal	Cancers	69 (61, 77)	69 (61, 77)	69 (62, 76)
Sec Ca – Bone	Cancers	71 (62, 80)	67 (57, 77)	74 (66, 81)
Sec Ca – Bowel	Cancers	68 (60, 77)	67 (58, 75)	71 (62, 79)
Sec Ca – Brain	Cancers	67 (57, 76)	65 (55, 75)	69 (60, 77)
Sec Ca – Liver	Cancers	70 (61, 79)	70 (60, 79)	71 (63, 79)
Sec Ca – Lung	Cancers	70 (60, 79)	69 (58, 79)	71 (62, 78)
Sec Ca – Lymph Nodes	Cancers	63 (53, 73)	62 (51, 72)	66 (57, 74)
Sec Ca – other	Cancers	70 (60, 80)	68 (57.75, 79)	72 (63, 81)
Sec Ca – Peritoneum	Cancers	68 (59, 76)	67 (59, 76)	68 (59, 76)
Sec Ca – Pleura	Cancers	70 (61, 79)	70 (61, 78)	71 (63, 79)
AAA	Cardiovascular	74 (66, 81)	78 (70, 84)	73 (65, 80)
Atrial Fibrillation	Cardiovascular	74 (65, 82)	78 (69, 85)	71 (62, 79)
AV Block, first degree	Cardiovascular	80 (71, 86)	82 (74, 88)	78 (69, 85)
AV Block, second degree	Cardiovascular	76 (65, 83)	78 (66.75, 85)	75 (65, 82)
AV Block, third degree	Cardiovascular	77 (68, 84)	79 (69, 86)	77 (67, 83)
Bifascicular Block	Cardiovascular	82 (73, 87)	85 (78, 89)	80 (72.5, 86)
Cardiomyopathy – other	Cardiovascular	61 (49, 72)	64 (49, 75)	61 (49, 71)
CHD – nos	Cardiovascular	70 (60, 79)	73 (63, 82)	67 (58, 76)
Dilated cardiomyopathy	Cardiovascular	60 (49, 70)	63 (50, 73)	59 (49, 69)
Heart Failure	Cardiovascular	76 (66, 84)	79 (70, 86)	73 (63, 81)
HOCM	Cardiovascular	57 (43, 70)	65 (51, 76)	53 (40, 64)
Hypertension	Cardiovascular	59 (49, 69)	60 (50, 71)	58 (49, 67)
Intracerebral Haemorrhage	Cardiovascular	70 (56, 81)	74 (59, 83)	66 (54, 78)
Ischaemic Stroke	Cardiovascular	74 (63, 83)	77 (67, 85)	70 (60, 79)
LBBB	Cardiovascular	77 (67, 85)	79 (69, 86)	75 (66, 83)

Multiple valve disorder	Cardiovascular	77 (67, 84)	79 (70, 86)	75 (65, 83)
Myocardial Infarction	Cardiovascular	65 (55, 76)	72 (61, 82)	62 (53, 72)
Non-rheum Aortic valve dz	Cardiovascular	74 (63, 82)	77 (66, 84)	72 (61, 80)
Non-rheum Mitral valve dz	Cardiovascular	68 (53, 78)	69 (50, 79)	67 (55, 77)
PAD	Cardiovascular	68 (59, 77)	71 (60, 80)	66 (58, 75)
Pericardial Effusion	Cardiovascular	66 (50.25, 77)	68 (53, 78)	64 (49, 75)
Pri Pulmonary HTN	Cardiovascular	74 (60, 82)	75 (61, 83)	72 (58, 80)
Pulmonary Embolism	Cardiovascular	62 (46, 75)	63 (43, 76)	62 (49, 73)
Raynauds Disease	Cardiovascular	44 (29, 60)	42 (28, 58)	51 (35, 64)
RBBB	Cardiovascular	75 (61, 84)	78 (63, 87)	73 (60, 82)
Rheum Valve dz	Cardiovascular	70 (54, 80)	71 (53, 81)	69 (55, 79)
Sec Pulmonary HTN	Cardiovascular	77 (65, 84)	78 (66, 85)	76 (65, 83)
Sick Sinus Syndrome	Cardiovascular	75 (65, 83)	77 (68, 84)	73 (62, 81)
Stable Angina	Cardiovascular	64 (55, 73)	67 (57, 75)	62 (54, 71)
Stroke – nos	Cardiovascular	72 (61, 81)	75 (63, 83)	69 (59, 78)
Subarach Haemorrhage	Cardiovascular	51 (40, 65)	53 (42, 67)	49 (37, 62)
Subdural haematoma	Cardiovascular	76 (61, 84)	77 (63, 86)	75 (60, 83)
SVT	Cardiovascular	57 (39, 71)	56 (38, 71)	58 (41, 70)
TIA	Cardiovascular	70 (61, 79)	73 (63, 81)	68 (60, 77)
Trifascicular Block	Cardiovascular	82 (76, 87)	85 (77, 90)	81 (75, 86)
Unstable Angina	Cardiovascular	66 (57, 76)	71 (60, 80)	64 (55, 74)
Ventricular Tachycardia	Cardiovascular	66 (54, 76)	64 (49, 77)	66 (55, 76)
VTE (Excl PE)	Cardiovascular	58 (41, 72)	57 (38, 73)	58 (44, 70)
Abdominal Hernia	Digestive	46 (23, 62)	43 (18, 63)	46 (24, 61)
Alcoholic Liver Disease	Digestive	52 (43, 61)	52 (43, 61)	52 (43, 61)
Anal Fissure	Digestive	34 (23, 47)	32 (23, 44)	37 (24, 49)
Angiodysplasia of colon	Digestive	70 (61, 79)	71 (61, 79)	70 (61, 78)
Anorectal Fistula	Digestive	40 (30, 51)	38 (28, 48)	41 (31, 52)
Anorectal Prolapse	Digestive	60 (40, 75)	64 (46, 77)	47 (6, 65)
Appendicitis	Digestive	19 (13, 30)	20 (14, 31)	18 (12, 29)
Autoimmune liver disease	Digestive	57 (45, 68)	58 (47, 68)	54 (39, 66)
Barretts Oesophagus	Digestive	64 (54, 74)	67 (57, 77)	63 (52, 72)
Cholangitis	Digestive	70 (55, 81)	70 (54, 82)	70 (56, 79)
Cholecystitis	Digestive	53 (40, 67)	50 (37, 64)	60 (48, 71)
Cholelithiasis	Digestive	55 (41, 68)	52 (38, 66)	61 (48, 73)
Cirrhosis	Digestive	54 (44, 64)	55 (45, 66)	54 (44, 63)
Coeliac Disease	Digestive	41 (23, 57)	40 (24, 56)	43 (17, 59.75)
Crohns Disease	Digestive	35 (24, 52)	35 (24, 52)	35 (23, 51)
Diaphragmatic Hernia	Digestive	60 (47, 71)	61 (50, 72)	57 (44, 68)
Diverticular Disease	Digestive	67 (57, 75)	67 (58, 76)	65 (56, 74)
Fatty Liver	Digestive	54 (43, 64)	55 (45, 66)	53 (43, 63)
Gastritis	Digestive	51 (35, 66)	52 (36, 67)	49 (34, 65)
GORD	Digestive	50 (34, 64)	51 (35, 65)	47 (32, 62)
IBS	Digestive	37 (27, 51)	37 (26, 50)	39 (28, 51)
Liver Failure	Digestive	57 (45, 69.75)	56 (43, 70)	58 (46, 69)
Oesophageal Ulcer	Digestive	53 (40, 66)	55 (41, 67)	51 (38, 64)
Oesophageal Varices	Digestive	57 (47, 67)	58 (47, 69)	56 (46, 65)
Pancreatitis	Digestive	53 (38, 67)	53 (38, 68)	52 (39, 66)
Peptic Ulcer	Digestive	51 (36, 67)	56 (41, 71)	48 (33, 64)
Peritonitis	Digestive	52 (32, 69)	55 (34, 72)	49 (29, 65)
Portal Hypertension	Digestive	56 (46, 66)	57 (46, 68)	56 (46, 65)
Ulcerative Colitis	Digestive	41 (29, 57)	41 (29, 57)	42 (29, 57)
Volvulus	Digestive	65 (48, 78)	64 (48, 78)	66 (49, 79)
Deafness	Ear	52 (23, 70)	54 (26, 73)	50 (19, 67)
Menieres Disease	Ear	55 (44, 67)	55 (44, 68)	55 (44, 66)
Tinnitus	Ear	53 (41, 64)	54 (41, 66)	52 (40, 63)
Cystic Fibrosis	Endocrine	30 (2, 45)	36 (13, 47)	3 (0, 23)
DM – other or nos	Endocrine	57 (41, 71)	57 (38, 72)	58 (44, 70)
Hyperparathyroidism	Endocrine	67 (54, 77.5)	68 (56, 78)	63 (48, 75)
Low HDL	Endocrine	57 (46, 67)	57 (46, 68)	57 (47, 66)
Obesity	Endocrine	45 (33, 58)	43 (31, 56)	48 (38, 59)
PCOS	Endocrine	27 (22, 32)	27 (22, 32)	NA (NA, NA)
Raised LDL	Endocrine	57 (48, 66)	59 (50, 68)	55 (46, 64)
Raised Total Cholesterol	Endocrine	56 (47, 65)	58 (49, 67)	54 (45, 63)
Raised Triglycerides	Endocrine	57 (48, 66)	60 (51, 69)	55 (46, 64)
SIADH	Endocrine	78 (67, 85)	80 (71, 86)	74 (63, 82)
T1DM	Endocrine	23 (12, 37)	21 (11, 37)	24 (13, 38)
T2DM	Endocrine	61 (51, 70)	63 (52, 73)	60 (50, 69)
Thyroid Disease	Endocrine	52 (39, 66)	51 (38, 65)	57 (43, 70)

Anterior Uveitis	Eye	45 (32, 60)	48 (34, 63)	42 (31, 57)
Blindness	Eye	68 (44, 81)	73 (50, 83)	62 (38, 78)
Cataract	Eye	74 (66, 80)	75 (67, 81)	72 (63, 79)
Diabetic Eye Disease	Eye	64 (52, 73)	66 (54, 75)	62 (51, 71)
Glaucoma	Eye	68 (58, 77)	70 (60, 78)	66 (56, 75)
Keratitis	Eye	44 (29, 61)	45 (29, 62)	43 (29, 59)
Macular Degeneration	Eye	77 (69, 84)	78 (70, 84)	76 (67, 82)
Posterior Uveitis	Eye	40 (26, 56)	40 (26, 56)	39 (26, 55)
Ptosis	Eye	55 (24, 71)	57 (35, 72)	51 (10, 69)
Retinal Detachment	Eye	58 (44, 67)	60 (47, 68)	56 (41, 66)
Retinal Vascular Occlusion	Eye	69 (60, 78)	72 (62, 80)	67 (58, 76)
Scleritis	Eye	45 (34, 57)	46 (35, 58)	44 (32, 57)
AKI	Genitourinary	79 (68, 87)	81 (71, 88)	77 (66, 85)
BPH	Genitourinary	66 (59, 74)	NA (NA, NA)	66 (59, 74)
Chronic Cystitis	Genitourinary	59 (41, 72)	53 (36, 67)	69 (59, 77)
CKD	Genitourinary	74 (68, 81)	75 (68, 82)	74 (67, 80)
Dysmenorrhoea	Genitourinary	25 (17, 37)	25 (17, 37)	NA (NA, NA)
End Stage Renal Disease	Genitourinary	64 (45, 78)	65 (45, 79)	63 (44, 77)
Endometrial Hyperplasia	Genitourinary	48 (41, 55)	48 (41, 55)	NA (NA, NA)
Endometriosis	Genitourinary	35 (29, 43)	35 (29, 43)	NA (NA, NA)
Erectile Dysfunction	Genitourinary	56 (46, 64)	NA (NA, NA)	56 (46, 64)
Female Infertility	Genitourinary	31 (27, 35)	31 (27, 35)	NA (NA, NA)
Glomerulonephritis	Genitourinary	67 (40, 81)	70 (44, 82)	65 (38, 79)
Hydrocele	Genitourinary	19 (2, 54)	NA (NA, NA)	19 (2, 54)
Male infertility	Genitourinary	34 (29, 38)	NA (NA, NA)	34 (29, 38)
Menorrhagia	Genitourinary	39 (29, 45)	39 (29, 45)	NA (NA, NA)
Neuropahtic Bladder	Genitourinary	56 (41, 69)	54 (40, 68)	58 (43, 70)
Obstruct/reflux uropathy	Genitourinary	49 (20, 70)	43 (20, 67)	54 (21, 72)
Postcoital Bleeding	Genitourinary	34 (26, 42)	34 (26, 42)	NA (NA, NA)
Postmenopausal Bleeding	Genitourinary	57 (53, 64)	57 (53, 64)	NA (NA, NA)
Prolapse	Genitourinary	57 (46, 67)	57 (46, 67)	NA (NA, NA)
Tubulo-interstitial Nephro	Genitourinary	33 (22, 54)	30 (22, 48)	53 (35, 68)
Undescended Testis	Genitourinary	3 (1, 9)	NA (NA, NA)	3 (1, 9)
Urinary Incontinence	Genitourinary	52 (40, 69)	51 (40, 66)	66 (41, 78)
Urolithiasis	Genitourinary	45 (33, 58)	43 (31, 57)	45 (34, 58)
Agranulocytosis	Haem/Imm	54 (36, 67)	53 (38, 66)	55 (32, 69)
Anaemia – other	Haem/Imm	57 (35, 76)	48 (32, 74)	69 (53, 79)
Aplastic Anaemia	Haem/Imm	64 (45, 77)	64 (44, 78)	65 (47, 77)
B12 def anaemia	Haem/Imm	64 (46, 77)	60 (43, 76)	69 (55, 78)
Folate Def Anaemia	Haem/Imm	72 (52, 84)	69 (46, 84)	74 (61, 84)
Hypersplenism	Haem/Imm	47 (26, 64)	46 (24, 65)	48 (28, 64)
Hyposplenism	Haem/Imm	35 (19, 58)	42 (21, 62)	31 (18, 54)
Immunodeficiency	Haem/Imm	35 (7, 62)	39 (12, 63)	29 (4, 62)
Iron Def Anaemia	Haem/Imm	48 (32, 72)	45 (31, 67)	66 (44, 77)
Other haemolytic anaemia	Haem/Imm	29 (5, 55)	30 (12, 53)	26 (2, 59)
Pri thrombocytopaenia	Haem/Imm	38 (13, 64)	37 (18, 63)	40 (8, 65)
Sarcoidosis	Haem/Imm	40 (31, 51)	42 (32, 54)	38 (31, 48)
Sec Polycythaemia	Haem/Imm	57 (46, 67)	59 (47, 70)	56 (45, 66)
Sec Thrombocytopaenia	Haem/Imm	56 (34, 72)	48 (30, 69)	62 (43, 74)
Sickle Cell Disease	Haem/Imm	18 (1, 34)	20 (1, 34)	15 (1, 33)
Sickle Cell Trait	Haem/Imm	27 (7, 36)	28 (17, 36)	19 (1, 38)
Thalassaemia	Haem/Imm	29 (16, 39)	29 (19, 39)	27 (7, 40)
Thalassaemia Trait	Haem/Imm	29 (18, 40)	29 (20.5, 38)	30 (13, 44)
Thrombophilia	Haem/Imm	38 (29, 51)	36 (28, 47)	46 (33, 58)
Chronic Hepatitis	Infections	38 (30, 47)	35 (28, 46)	39 (31, 48)
Encephalitis	Infections	45 (19, 67)	47 (21, 68)	44 (17, 66)
HIV	Infections	36 (29, 43)	33 (28, 40)	37 (30, 46)
ID-Bacterial	Infections	51 (29, 74)	50 (29, 76)	53 (30, 72)
ID-Fungal	Infections	58 (29, 76)	50 (26, 75)	64 (42, 77)
ID-Parasitic	Infections	34 (13, 54)	30 (12, 51)	38 (16, 55)
ID-Viral	Infections	3 (1, 29)	7 (1, 34)	2 (1, 19)
ID–Anorectal	Infections	38 (28, 50)	35 (25, 46.5)	40 (29, 51)
ID–Bone	Infections	56 (36, 73)	58 (37, 76)	55 (36, 70)
ID–Digestive	Infections	46 (6, 72)	50 (14, 75)	41 (3, 69)
ID–Eye	Infections	3 (0, 60)	9 (0, 70)	2 (0, 50)
ID–Heart	Infections	54 (36, 70)	60 (37.5, 75)	52 (35, 68)
ID–Liver	Infections	43 (33, 55)	40 (31, 55)	44 (35, 55)
ID–Male GU	Infections	37 (18, 58)	NA (NA, NA)	37 (18, 58)

ID–Other GU	Infections	30 (23, 40)	29 (23, 39)	55 (29, 73)
ID–Other nervous sys	Infections	57 (36, 73)	59 (37, 76)	56 (35, 70)
ID–Other organisms	Infections	38 (5, 70)	40 (8, 71)	35 (4, 68)
ID–Other organs	Infections	28 (3, 58)	30 (5, 57)	21 (2, 58)
ID–Skin	Infections	48 (26, 71)	53 (27, 76)	45 (26, 65)
LRTI	Infections	63 (28, 80)	65 (32, 82)	61 (19, 77)
Meningitis	Infections	26 (9, 38)	28 (17, 39)	23 (3, 38)
PID	Infections	37 (29, 46)	37 (29, 46)	NA (NA, NA)
Rheumatic Fever	Infections	44 (12, 73)	45 (12, 74)	44 (12, 71)
Sepsis	Infections	64 (23, 79)	65 (32, 81)	62 (8, 77)
Tuberculosis	Infections	24 (15, 36)	24 (16, 36)	25 (14.75, 37)
URTI	Infections	6 (2, 23)	8 (2, 26)	5 (2, 19)
UTI	Infections	71 (44, 83)	70 (38, 84)	73 (56, 82)
Ankylosing Spondylosis	Musculoskeletal	37 (28, 52)	42 (31, 58)	36 (27, 48)
Carpal Tunnel Syndrome	Musculoskeletal	50 (39, 62)	49 (38, 60)	52 (42, 64)
Collapsed Vertebra	Musculoskeletal	74 (62, 83)	76 (66, 84)	67 (49, 79)
Enteropathic Arthropathy	Musculoskeletal	47.5 (34, 62)	50 (37, 63)	43 (30, 60)
Enthesopathy	Musculoskeletal	48 (38, 59)	49 (39, 60)	48 (37, 58)
Fibromatosis	Musculoskeletal	62 (53, 69)	65 (57, 72)	60 (51, 68)
Fracture – Hip	Musculoskeletal	80 (68, 87)	82 (73, 87)	73 (49, 84)
Fracture – Wrist	Musculoskeletal	29 (12, 60)	54 (15, 69)	15 (11, 32)
Giant Cell Arteritis	Musculoskeletal	71 (63, 78)	72 (63, 78)	70 (62, 77)
Gout	Musculoskeletal	59 (47, 71)	69 (58, 78)	56 (45, 67)
Intervertebral Disc Disorder	Musculoskeletal	46 (37, 59)	48 (37, 61)	45 (36, 57)
Juvenile Arthritis	Musculoskeletal	11 (5, 15)	11 (5, 15)	11 (6, 15)
Lupus Erythematosus	Musculoskeletal	43 (32, 56)	42 (31, 55)	46 (34, 59)
Osteoarthritis	Musculoskeletal	61 (53, 71)	62 (53, 72)	60 (51, 69)
Osteoporosis	Musculoskeletal	71 (61, 80)	71 (62, 80)	70 (57, 80)
Polymyalgia Rheumatica	Musculoskeletal	72 (64, 78)	72 (64, 79)	72 (65, 78)
Psoriatic Arthritis	Musculoskeletal	46 (36, 57)	47 (36, 57)	45 (36, 56)
Reactive Arthritis	Musculoskeletal	34 (22, 47)	38 (23, 51)	31 (21, 44)
Rheumatoid Arthritis	Musculoskeletal	58 (45, 70)	57 (45, 71)	59 (46, 69)
Scelroderma	Musculoskeletal	53 (39, 65)	54 (40, 65)	50 (33, 62)
Scoliosis	Musculoskeletal	35 (16, 66)	39 (16, 71)	28 (15, 54)
Sjogren Syndrome	Musculoskeletal	58 (48, 67)	58 (48, 67)	58 (47, 69)
Spinal Stenosis	Musculoskeletal	67 (57, 76)	69 (58, 77)	65 (55, 75)
Spondylolisthesis	Musculoskeletal	59 (43, 72)	63 (49, 74)	51 (37, 66)
Spondylosis	Musculoskeletal	57 (47, 68)	57 (47, 69)	56 (46, 66)
Autonomic Neuropathy	Neurological	54 (40, 67)	53 (38, 66)	56 (42, 68)
Bells Syndrome	Neurological	42 (27, 58)	41 (26, 60)	42 (28, 57)
Cerebral Palsy	Neurological	3 (1, 16)	4 (1, 19)	3 (1, 14)
Chronic Fatigue Syndrome	Neurological	42 (31, 53)	43 (31, 53)	41 (29, 52)
Diabetic Neuropathy	Neurological	66 (56, 76)	68 (56, 77)	66 (56, 75)
Epilepsy	Neurological	25 (12, 48)	26 (13, 49)	25 (11, 47)
Essential Tremor	Neurological	65 (46, 74)	66 (49, 76)	63 (42, 73)
Idiopathic Intracranial HTN	Neurological	27 (20, 38)	28 (21, 37)	23 (12, 45.25)
Migraine	Neurological	32 (20, 44)	32 (22, 45)	29 (15, 43)
Motor Neurone Disease	Neurological	67 (53, 76)	68 (53.75, 77)	65 (51, 75)
Multiple Sclerosis	Neurological	40 (31, 50)	40 (31, 49)	41 (32, 50)
Myasthenia Gravis	Neurological	58 (36, 71)	51 (31, 69)	62 (49, 73)
Parkinsons Disease	Neurological	74 (66, 81)	75 (66, 82)	73 (65, 80)
Peripheral Neuropathy	Neurological	55 (43, 67)	55 (42, 67)	56 (43, 67)
Trigeminal Neuralgia	Neurological	55 (43, 67)	54 (43, 67)	56 (43, 68)
Congenital Septal Defect	Perinatal	2 (0, 22)	2 (0, 25)	1 (0, 17)
Downs Syndrome	Perinatal	0 (0, 13)	0 (0, 24)	0 (0, 3)
High Birth Weight	Perinatal	0 (0, 0)	0 (0, 0)	0 (0, 0)
Intrauterine Hypoxia	Perinatal	0 (0, 0)	0 (0, 0)	0 (0, 0)
Low Birth Weight	Perinatal	0 (0, 0)	0 (0, 0)	0 (0, 0)
Neonatal Jaundice	Perinatal	0 (0, 0)	0 (0, 0)	0 (0, 0)
Patent Ductus Arteriosus	Perinatal	0 (0, 1)	0 (0, 2)	0 (0, 1)
Post-term Delivery	Perinatal	0 (0, 0)	0 (0, 0)	0 (0, 0)
Premature Delivery	Perinatal	0 (0, 0)	0 (0, 0)	0 (0, 0)
Resp Distress Newborn	Perinatal	0 (0, 0)	0 (0, 0)	0 (0, 0)
Sepsis of the Newborn	Perinatal	0 (0, 0)	0 (0, 0)	0 (0, 0)
Spina Bifida	Perinatal	18 (2, 33)	18 (2, 35)	17 (2, 32)
Alcohol Misuse	Psychiatric	41 (30, 53)	41 (29, 52)	41 (31, 53)
Anxiety	Psychiatric	38 (27, 51)	38 (27, 52)	37 (26, 50)
Autism	Psychiatric	9 (5, 15)	11 (6, 18)	9 (5, 14)

Bipolar Affective Disorder	Psychiatric	39 (29, 51)	39 (29, 51)	39 (28, 51)
Delirium	Psychiatric	83 (74, 88)	84 (77, 90)	81 (71, 87)
Dementia	Psychiatric	83 (77, 88)	84 (78, 89)	81 (75, 86)
Depression	Psychiatric	37 (27, 50)	37 (26, 49)	39 (28, 50)
Eating Disorders	Psychiatric	21 (17, 28)	21 (17, 28)	22 (17, 32)
Hyperkinetic Disorders	Psychiatric	10 (7, 14)	11 (7, 17)	10 (7, 13)
Intellectual Disability	Psychiatric	19 (8, 38)	20 (9, 41)	18 (8, 37)
OCD	Psychiatric	29 (21, 40)	30 (22, 40)	29 (20, 41)
Personality Disorder	Psychiatric	30 (22, 41)	31 (22, 42)	30 (22, 40)
Schizophrenia	Psychiatric	34 (25, 48)	40 (28, 56)	31 (23, 42)
Substance Misuse	Psychiatric	28 (21, 39)	31 (22, 47)	27 (21, 36)
Allergic/chronic Rhinitis	Respiratory	27 (13, 44)	30 (15, 46)	24 (11, 42)
Asbestosis	Respiratory	70 (63, 78)	72 (58, 80)	70 (63, 77)
Aspiration Pneumonitis	Respiratory	78 (62, 87)	81 (66, 89)	76 (59, 85)
Asthma	Respiratory	19 (6, 42)	25 (9, 45)	13 (5, 37)
Bronchiectasis	Respiratory	64 (48, 75)	64 (48, 75)	65 (48, 75)
Chronic Sinusitis	Respiratory	42 (31, 55)	42 (31, 54)	43 (31, 56)
COPD	Respiratory	66 (57, 76)	67 (57, 77)	66 (58, 75)
Hypertroph Nasal Turbs	Respiratory	34 (21, 47)	34 (20, 48)	33 (22, 46)
Nasal Polyps	Respiratory	46 (33, 58)	44 (31, 57)	46 (34, 58)
Pleural Effusion	Respiratory	72 (57, 83)	75 (59, 85)	70 (56, 81)
Pleural Plaque	Respiratory	73 (65, 81)	76.5 (66, 85)	73 (65, 80)
Pneumothorax	Respiratory	31 (21, 56)	38 (24, 62)	29 (21, 53)
Pulmonary Collapse	Respiratory	67 (50, 79)	69 (52, 81)	65 (48, 76)
Pulmonary Fibrosis	Respiratory	74 (64, 82)	75 (64, 82)	74 (65, 81)
Respiratory Failure	Respiratory	73 (61, 82)	75 (62, 84)	71 (60, 81)
Sleep apnoea	Respiratory	48 (33, 59)	46 (12, 58)	48 (35, 59)
Acne	Skin	18 (15, 26)	20 (15, 29)	17 (15, 21)
Actinic keratosis	Skin	68 (59, 76)	68 (58, 77)	68 (60, 76)
Alopecia Areata	Skin	30 (18, 42)	32 (19, 45)	29 (17, 39)
Dermatitis	Skin	24 (3, 47)	27 (5, 47)	19 (2, 46)
Hidradenitis	Skin	34 (26, 44)	33 (25, 42)	37 (28, 47)
Lichen Planus	Skin	53 (39.5, 64)	55 (43, 65)	48 (36, 60)
Pilonidal cyst/sinus	Skin	25 (20, 34)	23 (19, 31)	27 (22, 35)
Psoriasis	Skin	37 (23, 54)	36 (22, 55)	38 (25, 53)
Rosacea	Skin	45 (33, 58)	44 (33, 55)	48 (33, 61)
Seborrheic Dermatitis	Skin	33 (13, 53)	33 (13, 53)	33 (11, 54)
Urticaria	Skin	28 (8, 47)	31 (12, 49)	19 (6, 43)
Vitiligo	Skin	33 (17, 48)	34 (19, 49)	31 (15, 47)

Supplementary Table S7B. Median (interquartile range) age of first record for 308 medical conditions by ethnicity.

Disease	Category	All ethnicities	Black	South Asian	White
Benign Neo – Brain	Benign Neo	55 (41, 69)	45 (34.75, 59)	49 (34, 60)	56 (42, 70)
Benign Neo – Colon	Benign Neo	63 (52, 72)	58.5 (46, 69)	59 (47, 68)	63 (53, 72)
Benign Neo – Ovary	Benign Neo	36 (27, 47)	34 (27, 41)	34 (27, 42)	37 (27, 47)
Benign Neo – Stomach	Benign Neo	66 (56, 75)	64.5 (52.75, 73)	62 (49, 73)	66 (56, 75)
Benign Neo – Uterus	Benign Neo	52 (44, 61)	44 (37, 52)	47 (38, 56)	53 (45, 62)
CIN	Benign Neo	31 (26, 39)	33 (27, 39)	35 (28, 43)	31 (26, 39)
Haemangioma	Benign Neo	34 (3, 53)	23 (1, 44)	21 (1, 43)	36 (4, 55)
Leiomyoma	Benign Neo	46 (40, 51)	40 (34, 45)	43 (37, 48)	47 (41, 52)
Hodgkins Lymphoma	Cancers	34 (23, 53)	34.5 (22.25, 46.5)	30.5 (20.5, 46.25)	35 (24, 55)
Leukaemia	Cancers	64 (47, 76)	43.5 (30, 59.25)	45 (12, 66)	65 (50, 76)
MGUS	Cancers	72 (63, 80)	64 (51.5, 76)	67 (54, 76)	73 (64, 80)
Myelodysplastic Syndrome	Cancers	76 (66, 83)	57 (41, 72)	65.5 (43.25, 75.75)	77 (67, 84)
Non Hodgkins Lymphoma	Cancers	62 (48, 73)	48 (36, 62.5)	49 (34.25, 65)	63 (50, 74)
Plasma Cell Ca	Cancers	70 (61, 79)	63 (53, 75)	59 (49.5, 73.5)	71 (61, 79)
Polycythaemia vera	Cancers	58 (47, 69)	49 (36.25, 59.75)	42 (29, 57)	59 (48, 70)
Pri Ca – Biliary	Cancers	72 (63, 81)	72 (61, 74)	71 (61.5, 80.75)	72 (63, 81)
Pri Ca – Bladder	Cancers	71 (62, 78)	67 (48, 75)	61.5 (51, 70)	71 (62, 79)
Pri Ca – Bone	Cancers	49 (25, 69)	31 (15.25, 54.5)	46 (26.5, 65.75)	51 (27, 69)
Pri Ca – Bowel	Cancers	69 (60, 78)	63 (51, 73)	65 (54, 73.5)	70 (61, 78)
Pri Ca – Brain	Cancers	52 (28, 68)	46 (18.25, 66)	40.5 (22, 53.25)	54 (30, 69)
Pri Ca – Breast	Cancers	59 (50, 68)	51 (44, 62)	53 (45, 62)	59 (50, 69)
Pri Ca – Cervix	Cancers	41 (34, 54)	47 (35, 56.25)	51 (39, 61.5)	41 (34, 54)
Pri Ca – Kidney	Cancers	67 (57, 76)	62 (46, 70)	64 (50.5, 72)	68 (58, 77)
Pri Ca – Liver	Cancers	69 (60, 77)	54.5 (47.5, 69.25)	61 (55.5, 74.5)	70 (61, 78)
Pri Ca – Lung	Cancers	71 (64, 79)	63 (51.75, 75)	69 (58.5, 76)	71 (64, 79)
Pri Ca – Melanoma	Cancers	57 (42.75, 70)	50 (31, 66)	55.5 (39, 62.75)	58 (44, 71)
Pri Ca – Mesothelioma	Cancers	72 (65, 79)	76 (70, 78.75)	66.5 (61.75, 71.25)	73 (66, 79)
Pri Ca – Multiple Sites	Cancers	74 (66, 83)	69 (66, 80)	NA (NA, NA)	74.5 (66, 83)
Pri Ca – Oesophageal	Cancers	70 (62, 79)	64 (58, 77)	66 (58, 78)	71 (62, 79)
Pri Ca – Oropharyngeal	Cancers	61 (51, 71)	50 (38.75, 62.5)	55 (41, 64)	62 (52, 72)
Pri Ca – other	Cancers	65 (51, 76)	48 (37, 63)	54 (31.5, 67)	65 (53, 76)
Pri Ca – Ovary	Cancers	61 (49, 71)	52 (44, 66)	53.5 (42, 66.75)	62 (50, 72)
Pri Ca – Pancreas	Cancers	72 (63, 81)	66.5 (53.25, 76.75)	68 (62.5, 74)	73 (63, 81)
Pri Ca – Prostate	Cancers	71 (64, 77)	68 (60, 74)	69.5 (63, 75)	71 (65, 78)
Pri Ca – Skin	Cancers	68 (57, 77)	59.5 (46.25, 73)	55 (41, 67)	68 (58, 77)
Pri Ca – Stomach	Cancers	72 (62, 79)	67 (55.5, 73.25)	65 (51, 75)	72 (63, 80)
Pri Ca – Testis	Cancers	34 (28, 42)	33 (23.5, 39.75)	32 (23, 42.25)	35 (28, 43)
Pri Ca – Thyroid	Cancers	47 (35, 61)	43.5 (37, 53.75)	39 (33, 50)	48 (36, 63)
Pri Ca – Uterus	Cancers	64 (57, 72)	64 (55.5, 70)	62 (54, 69)	65 (57, 73)
Sec Ca – Adrenal	Cancers	69 (61, 77)	58 (52.5, 65.5)	67.5 (62.75, 70)	69 (62, 77)
Sec Ca – Bone	Cancers	71 (62, 80)	67.5 (54, 76.75)	65 (52, 73)	71 (62, 80)
Sec Ca – Bowel	Cancers	68 (60, 77)	59 (52.5, 74)	67 (55, 72)	68 (60, 77)
Sec Ca – Brain	Cancers	67 (57, 76)	53 (47.75, 61.5)	60 (51, 70)	67 (58, 76)
Sec Ca – Liver	Cancers	70 (61, 79)	65 (52, 76)	64 (54, 72)	71 (62, 79)
Sec Ca – Lung	Cancers	70 (60, 79)	60 (50, 74)	61.5 (48, 70.25)	70 (61, 79)
Sec Ca – Lymph Nodes	Cancers	63 (53, 73)	53.5 (46, 65.75)	57 (46, 66)	64 (53, 73)
Sec Ca – other	Cancers	70 (60, 80)	64.5 (53.75, 76.25)	62.5 (51.75, 69)	70 (60, 80)
Sec Ca – Peritoneum	Cancers	68 (59, 76)	61 (51.25, 73)	61 (51, 69)	68 (59, 76)
Sec Ca – Pleura	Cancers	70 (61, 79)	56 (49, 64)	61.5 (54.75, 71)	71 (62, 79)
AAA	Cardiovascular	74 (66, 81)	66 (54, 74.5)	67 (57.5, 75)	74 (66, 81)
Atrial Fibrillation	Cardiovascular	74 (65, 82)	67 (53, 77)	69 (59, 77)	75 (65, 82)
AV Block, first degree	Cardiovascular	80 (71, 86)	70 (49, 78)	76 (66, 82)	80 (71, 87)
AV Block, second degree	Cardiovascular	76 (65, 83)	65 (44.5, 77)	72 (56, 79)	76 (66, 83)
AV Block, third	Cardiovascular	77 (68, 84)	73 (52.5, 80)	66.5 (55.75, 74)	78 (68, 85)

degree					
Bifascicular Block	Cardiovascular	82 (73, 87)	74.5 (67.75, 79)	76 (71.25, 83.5)	82 (74, 88)
Cardiomyopathy – other	Cardiovascular	61 (49, 72)	50 (37, 61.5)	61 (42.75, 73)	62 (50, 73)
CHD – nos	Cardiovascular	70 (60, 79)	61 (52, 73)	62 (51.75, 71)	71 (61, 80)
Dilated cardiomyopathy	Cardiovascular	60 (49, 70)	48 (39.75, 60)	54 (38, 66)	61 (50, 71)
Heart Failure	Cardiovascular	76 (66, 84)	66 (51, 76)	70 (60, 78)	77 (67, 84)
HOCM	Cardiovascular	57 (43, 70)	52 (37.25, 65)	46 (34.25, 64.25)	59 (45, 71)
Hypertension	Cardiovascular	59 (49, 69)	48 (40, 58)	53 (44, 63)	60 (51, 70)
Intracerebral Haemorrhage	Cardiovascular	70 (56, 81)	53 (41.25, 69)	58 (43, 70)	71 (57, 81)
Ischaemic Stroke	Cardiovascular	74 (63, 83)	63 (49, 75)	67 (53, 76)	74 (64, 83)
LBBB	Cardiovascular	77 (67, 85)	68 (54.5, 79)	71 (60, 79)	78 (67, 85)
Multiple valve disorder	Cardiovascular	77 (67, 84)	66 (50, 75)	70 (55.75, 77)	78 (68, 85)
Myocardial Infarction	Cardiovascular	65 (55, 76)	62 (50, 75)	60 (50, 71)	66 (55, 77)
Non-rheum Aortic valve dz	Cardiovascular	74 (63, 82)	66 (46, 77)	67 (51, 76.5)	75 (64, 82)
Non-rheum Mitral valve dz	Cardiovascular	68 (53, 78)	47 (35, 66)	58 (41, 71)	69 (55, 79)
PAD	Cardiovascular	68 (59, 77)	67 (55, 75)	65 (54, 74)	68 (59, 77)
Pericardial Effusion	Cardiovascular	66 (50.25, 77)	47.5 (27, 69)	52 (28.5, 69.5)	67 (53, 78)
Pri Pulmonary HTN	Cardiovascular	74 (60, 82)	50 (34.75, 72)	56 (12, 72)	74 (62, 83)
Pulmonary Embolism	Cardiovascular	62 (46, 75)	48 (36, 65)	47.5 (31, 66)	63 (47, 75)
Raynauds Disease	Cardiovascular	44 (29, 60)	38 (27, 49)	37 (25, 52)	46 (31, 62)
RBBB	Cardiovascular	75 (61, 84)	67.5 (47, 78)	69 (53, 77)	76 (62, 84)
Rheum Valve dz	Cardiovascular	70 (54, 80)	53 (36, 67)	50 (33.25, 69)	71 (56, 81)
Sec Pulmonary HTN	Cardiovascular	77 (65, 84)	62 (44, 75)	69 (42, 77)	78 (67, 84.5)
Sick Sinus Syndrome	Cardiovascular	75 (65, 83)	61 (39, 73.5)	69 (52.5, 78)	76 (66, 83)
Stable Angina	Cardiovascular	64 (55, 73)	62 (51.75, 71)	59 (51, 67)	64 (56, 73)
Stroke – nos	Cardiovascular	72 (61, 81)	63 (50, 72)	62 (53, 73)	72 (61, 81)
Subarach Haemorrhage	Cardiovascular	51 (40, 65)	46 (36, 55.25)	47 (34.75, 59)	52 (40, 66)
Subdural haematoma	Cardiovascular	76 (61, 84)	69.5 (47.5, 78)	68 (44, 79)	77 (63, 85)
SVT	Cardiovascular	57 (39, 71)	45.5 (30.25, 64)	45 (31, 62)	58 (40, 71)
TIA	Cardiovascular	70 (61, 79)	64 (51, 73)	64 (55, 74)	71 (61, 79)
Trifascicular Block	Cardiovascular	82 (76, 87)	73.5 (69, 83.5)	78 (72, 79)	82 (76, 87)
Unstable Angina	Cardiovascular	66 (57, 76)	64 (50, 74)	61 (52, 70)	67 (57, 77)
Ventricular Tachycardia	Cardiovascular	66 (54, 76)	52 (39, 69)	57 (41, 71.5)	66 (54, 77)
VTE (Excl PE)	Cardiovascular	58 (41, 72)	46 (35, 61)	47 (31, 63)	59 (42, 73)
Abdominal Hernia	Digestive	46 (23, 62)	23 (1, 42)	39 (13.75, 55.25)	48 (28, 63)
Alcoholic Liver Disease	Digestive	52 (43, 61)	45 (41, 55.5)	48 (38.25, 56)	52 (43, 61)
Anal Fissure	Digestive	34 (23, 47)	33 (23, 41)	32 (24, 42)	35 (24, 49)
Angiodysplasia of colon	Digestive	70 (61, 79)	69 (57, 74.25)	61 (53, 73)	71 (62, 79)
Anorectal Fistula	Digestive	40 (30, 51)	38 (31, 46)	36.5 (30, 46)	41 (30, 52)
Anorectal Prolapse	Digestive	60 (40, 75)	45.5 (37, 62)	50 (36, 62)	62 (43, 76)
Appendicitis	Digestive	19 (13, 30)	23 (15, 33)	21 (14, 30)	20 (13, 31)
Autoimmune liver disease	Digestive	57 (45, 68)	45 (38, 54)	47 (34.5, 58)	58.5 (46, 69)
Barretts Oesophagus	Digestive	64 (54, 74)	67 (51.25, 75.25)	61 (50, 71)	65 (54, 74)
Cholangitis	Digestive	70 (55, 81)	46 (33, 57)	60 (40.75, 76)	71 (56, 81)
Cholecystitis	Digestive	53 (40, 67)	42 (33, 54)	48 (35, 61)	54 (40, 67)
Cholelithiasis	Digestive	55 (41, 68)	41 (32, 54)	46 (34, 60)	56 (42, 69)
Cirrhosis	Digestive	54 (44, 64)	51 (42, 61)	51 (38.5, 60)	55 (45, 65)
Coeliac Disease	Digestive	41 (23, 57)	29.5 (7, 43.75)	34 (19, 47)	43 (24, 59)
Crohns Disease	Digestive	35 (24, 52)	33 (23, 42)	32 (23, 45)	35 (24, 53)
Diaphragmatic Hernia	Digestive	60 (47, 71)	49 (40, 64)	52 (40, 65)	60 (48, 71)
Diverticular Disease	Digestive	67 (57, 75)	63 (52, 73)	64 (55, 72)	67 (58, 75)
Fatty Liver	Digestive	54 (43, 64)	46 (38.25, 54)	47 (36, 57)	55 (44, 65)
Gastritis	Digestive	51 (35, 66)	42 (31, 55)	43 (30, 59)	53 (37, 67)
GORD	Digestive	50 (34, 64)	41 (30, 53)	40 (28, 55)	51 (35, 65)
IBS	Digestive	37 (27, 51)	35 (27, 44)	34 (26, 44)	38 (27, 52)
Liver Failure	Digestive	57 (45, 69.75)	50 (35.5, 68.5)	55 (35, 69.75)	58 (45, 70)
Oesophageal Ulcer	Digestive	53 (40, 66)	44 (34, 57)	45 (33, 59)	55 (41, 67)

Oesophageal Varices	Digestive	57 (47, 67)	53 (43, 66.75)	52 (40, 60)	57 (47, 67)
Pancreatitis	Digestive	53 (38, 67)	43 (34, 56)	46 (33, 60)	54 (39, 68)
Peptic Ulcer	Digestive	51 (36, 67)	41 (31, 56)	47 (34, 62)	53 (38, 68)
Peritonitis	Digestive	52 (32, 69)	42 (26.5, 55)	40 (23, 56)	53 (33, 70)
Portal Hypertension	Digestive	56 (46, 66)	47.5 (37, 62.25)	54 (41, 63)	56 (47, 66)
Ulcerative Colitis	Digestive	41 (29, 57)	37.5 (29, 48.25)	34 (25, 45)	42 (29, 59)
Volvulus	Digestive	65 (48, 78)	53 (35, 74)	55 (41, 73)	66 (50, 79)
Deafness	Ear	52 (23, 70)	37 (10, 56.5)	40 (12, 61)	55 (28, 72)
Menieres Disease	Ear	55 (44, 67)	48 (40, 61)	49 (40, 59.75)	57 (45, 68)
Tinnitus	Ear	53 (41, 64)	48 (37, 61)	48 (36, 60)	55 (42, 65)
Cystic Fibrosis	Endocrine	30 (2, 45)	9.5 (0, 32.75)	8 (0, 37)	29 (2, 45)
DM – other or nos	Endocrine	57 (41, 71)	45 (35, 55)	45 (34, 58)	60 (42, 73)
Hyperparathyroidism	Endocrine	67 (54, 77.5)	56 (43, 72)	56 (45.75, 71)	68 (56, 78)
Low HDL	Endocrine	57 (46, 67)	47 (40, 58)	47 (38, 58)	59 (48, 68)
Obesity	Endocrine	45 (33, 58)	40 (32, 48)	39 (30, 51)	47 (34, 59)
PCOS	Endocrine	27 (22, 32)	29 (24, 33)	27 (23, 31)	27 (23, 32)
Raised LDL	Endocrine	57 (48, 66)	48 (42, 58)	48 (40, 57)	59 (49, 67)
Raised Total Cholesterol	Endocrine	56 (47, 65)	48 (42, 58)	47 (39, 56)	57 (48, 66)
Raised Triglycerides	Endocrine	57 (48, 66)	50 (42, 60)	48 (40, 58)	58 (49, 67)
SIADH	Endocrine	78 (67, 85)	59 (50, 76)	74 (62.5, 79.5)	78 (67, 85)
T1DM	Endocrine	23 (12, 37)	29 (14, 41)	26 (13, 40)	23 (12, 37)
T2DM	Endocrine	61 (51, 70)	52 (44, 63)	52 (43, 61)	63 (53, 72)
Thyroid Disease	Endocrine	52 (39, 66)	42 (33, 53)	40 (30, 53)	54 (40, 68)
Anterior Uveitis	Eye	45 (32, 60)	44 (32, 58)	44 (32, 58)	47 (33, 62)
Blindness	Eye	68 (44, 81)	52 (32, 73)	51 (26, 69)	70 (48, 82)
Cataract	Eye	74 (66, 80)	67 (58, 73.5)	66 (58, 72)	75 (67, 81)
Diabetic Eye Disease	Eye	64 (52, 73)	58 (48, 69)	57 (49, 67)	65 (54, 74)
Glaucoma	Eye	68 (58, 77)	59 (48, 69)	62 (51, 70)	69 (59, 78)
Keratitis	Eye	44 (29, 61)	37 (22, 49)	32 (22, 47)	47 (31, 63)
Macular Degeneration	Eye	77 (69, 84)	67 (55, 75)	64 (53, 73)	78 (70, 84)
Posterior Uveitis	Eye	40 (26, 56)	36 (30.25, 44)	44 (29, 54)	41 (26, 57)
Ptosis	Eye	55 (24, 71)	36 (5.5, 53.5)	36 (10, 59)	58 (31, 72)
Retinal Detachment	Eye	58 (44, 67)	48 (36, 60)	53 (33, 63)	59 (46, 68)
Retinal Vascular Occlusion	Eye	69 (60, 78)	62 (49, 71)	61 (52, 70)	70 (61, 79)
Scleritis	Eye	45 (34, 57)	40 (31, 48)	38 (27, 48)	47 (36, 59)
AKI	Genitourinary	79 (68, 87)	64 (47, 77)	72 (57, 80)	80 (69, 87)
BPH	Genitourinary	66 (59, 74)	63 (54, 71)	64 (56, 72)	67 (60, 74)
Chronic Cystitis	Genitourinary	59 (41, 72)	46 (35, 55)	48 (32, 61.25)	61 (42, 73)
CKD	Genitourinary	74 (68, 81)	68 (58, 76)	70 (62, 76)	75 (68, 81)
Dysmenorrhoea	Genitourinary	25 (17, 37)	27 (18, 37)	26 (19, 36)	26 (17, 38)
End Stage Renal Disease	Genitourinary	64 (45, 78)	53 (39, 67)	56 (39.75, 69.25)	66 (46, 79)
Endometrial Hyperplasia	Genitourinary	48 (41, 55)	42 (36, 48)	44 (37, 49.25)	48 (41, 56)
Endometriosis	Genitourinary	35 (29, 43)	36 (30, 42)	35 (30, 42)	35 (29, 43)
Erectile Dysfunction	Genitourinary	56 (46, 64)	50 (41, 60)	49 (38, 58)	57 (48, 65)
Female Infertility	Genitourinary	31 (27, 35)	34 (29, 38)	30 (27, 35)	31 (27, 35)
Glomerulonephritis	Genitourinary	67 (40, 81)	45 (31, 64)	41 (22, 66)	70 (46, 81.5)
Hydrocele	Genitourinary	19 (2, 54)	21 (3, 51)	7 (0, 41)	23 (3, 58)
Male infertility	Genitourinary	34 (29, 38)	37 (32, 41)	33 (30, 37)	33 (29, 38)
Menorrhagia	Genitourinary	39 (29, 45)	38 (30, 43)	36 (28, 44)	39 (29, 45)
Neuropahtic Bladder	Genitourinary	56 (41, 69)	46 (37, 58)	48 (36, 61)	57 (43, 70)
Obstruct/reflux uropathy	Genitourinary	49 (20, 70)	39 (21, 54)	34 (5, 53)	52 (24, 72)
Postcoital Bleeding	Genitourinary	34 (26, 42)	35 (28, 43)	33 (27, 40)	34 (26, 42)
Postmenopausal Bleeding	Genitourinary	57 (53, 64)	55 (51, 60)	55 (52, 61)	57 (53, 64)
Prolapse	Genitourinary	57 (46, 67)	47 (38, 60)	49 (40, 59.25)	57 (47, 68)
Tubulo-interstitial Nephro	Genitourinary	33 (22, 54)	33 (24, 43)	35 (25, 51)	34 (22, 55)
Undescended Testis	Genitourinary	3 (1, 9)	2 (1, 7)	1 (0, 6)	3 (1, 9)
Urinary Incontinence	Genitourinary	52 (40, 69)	45 (33, 59)	46 (34, 59)	54 (41, 70)
Urolithiasis	Genitourinary	45 (33, 58)	42 (33.75, 52)	39 (30, 50)	46 (34, 59)
Agranulocytosis	Haem/Imm	54 (36, 67)	40 (28, 52)	37 (15.75, 55)	57 (39, 68)
Anaemia – other	Haem/Imm	57 (35, 76)	39 (29, 51)	37 (26, 57)	63 (39, 78)
Aplastic Anaemia	Haem/Imm	64 (45, 77)	44.5 (30.75, 66.25)	44 (22, 65)	66 (49, 78)
B12 def anaemia	Haem/Imm	64 (46, 77)	46 (39, 66.5)	51 (38, 64)	66 (48, 78)
Folate Def Anaemia	Haem/Imm	72 (52, 84)	48 (41, 66)	46 (32, 65.25)	73 (55, 84)

Hypersplenism	Haem/Imm	47 (26, 64)	25 (5, 38)	39 (22.5, 55)	50 (29, 66)
Hyposplenism	Haem/Imm	35 (19, 58)	35 (18.75, 49)	35.5 (22.75, 53)	38 (19, 60.5)
Immunodeficiency	Haem/Imm	35 (7, 62)	32 (10, 43)	11 (2, 41)	39 (8, 65)
Iron Def Anaemia	Haem/Imm	48 (32, 72)	38 (27, 46)	34 (23, 48)	53 (36, 75)
Other haemolytic anaemia	Haem/Imm	29 (5, 55)	29 (5, 41)	26 (6, 36)	32.5 (7, 63)
Pri thrombocytopaenia	Haem/Imm	38 (13, 64)	39 (29, 51)	34.5 (19, 54)	42 (15, 66)
Sarcoidosis	Haem/Imm	40 (31, 51)	39 (31, 47)	43 (34, 52)	40 (31, 52)
Sec Polycythaemia	Haem/Imm	57 (46, 67)	51.5 (28, 66.75)	45 (32, 56.5)	58 (47, 68)
Sec Thrombocytopaenia	Haem/Imm	56 (34, 72)	39 (29, 55)	39 (26, 60)	59 (36, 74)
Sickle Cell Disease	Haem/Imm	18 (1, 34)	11 (1, 30)	26 (4, 34)	39 (16.5, 59.5)
Sickle Cell Trait	Haem/Imm	27 (7, 36)	28 (11.75, 37)	27 (12.5, 33)	25 (5, 37.25)
Thalassaemia	Haem/Imm	29 (16, 39)	29 (12, 39)	28 (16, 36)	30.5 (18, 45)
Thalassaemia Trait	Haem/Imm	29 (18, 40)	34 (25.5, 43)	28 (17, 38)	30 (19, 44)
Thrombophilia	Haem/Imm	38 (29, 51)	39.5 (31.25, 47.75)	34 (29, 43)	38 (29, 52)
Chronic Hepatitis	Infections	38 (30, 47)	36 (29, 46)	37 (29, 48)	39 (30, 48)
Encephalitis	Infections	45 (19, 67)	17.5 (5.75, 43.25)	23 (7, 48)	48 (23, 68.75)
HIV	Infections	36 (29, 43)	34 (28, 41)	35.5 (31, 44.75)	38 (30, 46)
ID-Bacterial	Infections	51 (29, 74)	36 (22, 49)	35 (21, 54)	54 (31, 75)
ID-Fungal	Infections	58 (29, 76)	34 (20, 48.5)	32 (21, 61)	60 (31, 77)
ID-Parasitic	Infections	34 (13, 54)	36 (23, 45)	29 (9.5, 45)	34 (13, 58)
ID-Viral	Infections	3 (1, 29)	4 (1, 28)	3 (1, 17)	4 (1, 31)
ID–Anorectal	Infections	38 (28, 50)	34 (24, 43)	33 (25, 44)	39 (28, 50)
ID–Bone	Infections	56 (36, 73)	38 (20, 55)	43 (26, 62)	58 (39, 74)
ID–Digestive	Infections	46 (6, 72)	32 (4, 50)	28 (3, 54)	50 (7, 74)
ID–Eye	Infections	3 (0, 60)	5 (0, 33.25)	2 (0, 24.5)	4 (0, 65)
ID–Heart	Infections	54 (36, 70)	41 (30.75, 52.25)	43 (29, 63)	56 (38, 71)
ID–Liver	Infections	43 (33, 55)	38 (31, 48)	40 (29, 53)	44 (34, 57)
ID–Male GU	Infections	37 (18, 58)	38 (18.5, 55)	33.5 (19, 50)	38 (19, 60)
ID–Other GU	Infections	30 (23, 40)	29 (23, 36)	29 (25, 35)	30 (23, 41)
ID–Other nervous sys	Infections	57 (36, 73)	35 (22, 47.25)	38 (25, 65)	60 (40, 74)
ID–Other organisms	Infections	38 (5, 70)	18 (3, 43)	13 (2, 45)	42 (6, 71)
ID–Other organs	Infections	28 (3, 58)	27 (4, 41)	18 (2, 39)	30 (3, 60)
ID–Skin	Infections	48 (26, 71)	31 (13, 45)	29 (10, 46)	51 (29, 73)
LRTI	Infections	63 (28, 80)	30 (3, 51)	31 (2, 62)	65 (33, 80)
Meningitis	Infections	26 (9, 38)	27 (7, 39)	29 (16, 40)	26 (9, 39)
PID	Infections	37 (29, 46)	36 (31, 43)	36 (30, 43)	37 (29, 47)
Rheumatic Fever	Infections	44 (12, 73)	44 (27, 64)	39 (18.5, 64.5)	51 (13, 75)
Sepsis	Infections	64 (23, 79)	26 (0, 54)	29 (0, 63)	66 (33, 80)
Tuberculosis	Infections	24 (15, 36)	29 (21, 37.75)	30 (23, 41)	22 (14, 35)
URTI	Infections	6 (2, 23)	5 (2, 23)	4 (2, 18)	6 (2, 23)
UTI	Infections	71 (44, 83)	45 (28, 71)	45 (26, 70)	73 (47, 84)
Ankylosing Spondylosis	Musculoskeletal	37 (28, 52)	38 (31, 50)	35 (28.5, 47)	38 (29, 54)
Carpal Tunnel Syndrome	Musculoskeletal	50 (39, 62)	45.5 (37, 55)	42 (34, 53)	51 (40, 63)
Collapsed Vertebra	Musculoskeletal	74 (62, 83)	64 (39, 73)	67 (48, 76.5)	75 (63, 83)
Enteropathic Arthropathy	Musculoskeletal	47.5 (34, 62)	34 (27.75, 46.75)	37 (29.5, 47.5)	49 (34, 62)
Enthesopathy	Musculoskeletal	48 (38, 59)	44 (35, 53)	44 (35, 54)	50 (39, 61)
Fibromatosis	Musculoskeletal	62 (53, 69)	56.5 (47, 64.25)	61 (53, 68)	62 (54, 70)
Fracture – Hip	Musculoskeletal	80 (68, 87)	49 (30, 77.5)	72 (47, 81)	80 (69, 87)
Fracture – Wrist	Musculoskeletal	29 (12, 60)	15.5 (10, 35)	18 (11, 48)	34 (13, 62)
Giant Cell Arteritis	Musculoskeletal	71 (63, 78)	63 (52.5, 72)	63 (55, 70)	72 (64, 78)
Gout	Musculoskeletal	59 (47, 71)	55 (44, 67)	52 (41, 64)	61 (49, 72)
Intervertebral Disc Disorder	Musculoskeletal	46 (37, 59)	47 (39, 58)	45 (36, 57)	47 (37, 60)
Juvenile Arthritis	Musculoskeletal	11 (5, 15)	12.5 (9, 23)	11 (5, 14.5)	11 (5, 15)
Lupus Erythematosus	Musculoskeletal	43 (32, 56)	36 (26, 46)	38 (28, 49)	44 (33, 57)
Osteoarthritis	Musculoskeletal	61 (53, 71)	55 (47, 66)	58 (50, 66)	62 (53, 71)
Osteoporosis	Musculoskeletal	71 (61, 80)	61 (47, 74)	63 (54, 72)	72 (62, 80)
Polymyalgia Rheumatica	Musculoskeletal	72 (64, 78)	68 (55, 76)	62 (52, 71)	73 (65, 79)
Psoriatic Arthritis	Musculoskeletal	46 (36, 57)	45 (37, 51)	41 (31, 51)	47 (36, 57)
Reactive Arthritis	Musculoskeletal	34 (22, 47)	31 (9, 44.5)	33 (6, 45)	34 (22, 48)
Rheumatoid Arthritis	Musculoskeletal	58 (45, 70)	49 (36, 62)	49 (36, 61)	59 (46, 71)
Scelroderma	Musculoskeletal	53 (39, 65)	48.5 (30.25,	48.5 (36.75, 56)	55 (41, 66)

			60.25)		
Scoliosis	Musculoskeletal	35 (16, 66)	22 (15, 42)	29 (15, 53)	39 (16, 70)
Sjogren Syndrome	Musculoskeletal	58 (48, 67)	42 (35, 59)	52 (38.5, 59)	59 (49, 68)
Spinal Stenosis	Musculoskeletal	67 (57, 76)	64 (51, 73)	62 (50, 72)	68 (57, 76)
Spondylolisthesis	Musculoskeletal	59 (43, 72)	60.5 (52, 70)	58 (44.25, 67)	60 (44, 72)
Spondylosis	Musculoskeletal	57 (47, 68)	54 (46, 65)	52.5 (44, 63)	58 (48, 68)
Autonomic Neuropathy	Neurological	54 (40, 67)	57 (39, 68.25)	52 (37.5, 66)	54 (40, 68)
Bells Syndrome	Neurological	42 (27, 58)	35 (22, 49)	36 (24, 51)	44 (28, 60)
Cerebral Palsy	Neurological	3 (1, 16)	3 (1, 12.5)	4 (1, 10.5)	4 (1, 17)
Chronic Fatigue Syndrome	Neurological	42 (31, 53)	42 (34, 51)	42 (34, 51)	43 (31, 54)
Diabetic Neuropathy	Neurological	66 (56, 76)	61 (51.75, 72)	61 (52, 70)	67 (56, 76)
Epilepsy	Neurological	25 (12, 48)	23 (8, 42)	22 (9, 40)	27 (13, 51)
Essential Tremor	Neurological	65 (46, 74)	57.5 (42.25, 71.75)	56 (32, 69)	66 (50, 76)
Idiopathic Intracranial HTN	Neurological	27 (20, 38)	29 (20.5, 37)	32 (22.5, 41.5)	27 (20, 38)
Migraine	Neurological	32 (20, 44)	31 (23, 40)	31 (23, 40)	32 (21, 45)
Motor Neurone Disease	Neurological	67 (53, 76)	60 (48.75, 75.75)	57 (25.5, 71.5)	67 (54, 76)
Multiple Sclerosis	Neurological	40 (31, 50)	38.5 (31.75, 47.25)	33 (27, 41)	40 (32, 50)
Myasthenia Gravis	Neurological	58 (36, 71)	37 (32, 45.5)	57 (40, 69)	60 (40, 73)
Parkinsons Disease	Neurological	74 (66, 81)	72 (65, 78)	70 (62, 76)	74 (66, 81)
Peripheral Neuropathy	Neurological	55 (43, 67)	51 (40, 64)	53 (40, 65)	56 (43, 68)
Trigeminal Neuralgia	Neurological	55 (43, 67)	49 (39.75, 58)	50 (38, 61)	56 (43, 68)
Congenital Septal Defect	Perinatal	2 (0, 22)	1 (0, 6.75)	1 (0, 6)	2 (0, 28)
Downs Syndrome	Perinatal	0 (0, 13)	0 (0, 5)	0 (0, 18.25)	0 (0, 15)
High Birth Weight	Perinatal	0 (0, 0)	0 (0, 0)	0 (0, 0)	0 (0, 0)
Intrauterine Hypoxia	Perinatal	0 (0, 0)	0 (0, 0)	0 (0, 0)	0 (0, 0)
Low Birth Weight	Perinatal	0 (0, 0)	0 (0, 0)	0 (0, 0)	0 (0, 0)
Neonatal Jaundice	Perinatal	0 (0, 0)	0 (0, 0)	0 (0, 0)	0 (0, 0)
Patent Ductus Arteriosus	Perinatal	0 (0, 1)	0 (0, 1)	0 (0, 1)	0 (0, 1)
Post-term Delivery	Perinatal	0 (0, 0)	0 (0, 0)	0 (0, 0)	0 (0, 0)
Premature Delivery	Perinatal	0 (0, 0)	0 (0, 0)	0 (0, 0)	0 (0, 0)
Resp Distress Newborn	Perinatal	0 (0, 0)	0 (0, 0)	0 (0, 0)	0 (0, 0)
Sepsis of the Newborn	Perinatal	0 (0, 0)	0 (0, 0)	0 (0, 0)	0 (0, 0)
Spina Bifida	Perinatal	18 (2, 33)	15.5 (0, 34)	3.5 (0, 24.25)	19 (3, 35)
Alcohol Misuse	Psychiatric	41 (30, 53)	39 (30, 48)	36 (28, 47)	42 (31, 54)
Anxiety	Psychiatric	38 (27, 51)	36 (27, 45)	35 (26, 46)	38 (27, 52)
Autism	Psychiatric	9 (5, 15)	5 (4, 9)	6 (4, 10)	9 (5, 15)
Bipolar Affective Disorder	Psychiatric	39 (29, 51)	33 (25, 41)	34 (25, 45)	40 (29, 52)
Delirium	Psychiatric	83 (74, 88)	73 (54, 80)	75 (59, 82.75)	83 (75, 89)
Dementia	Psychiatric	83 (77, 88)	77 (72, 82)	78 (71, 83)	83 (77, 88)
Depression	Psychiatric	37 (27, 50)	35 (27, 45)	36 (28, 47)	38 (27, 51)
Eating Disorders	Psychiatric	21 (17, 28)	24 (17, 33.5)	21 (18, 27)	21 (17, 28)
Hyperkinetic Disorders	Psychiatric	10 (7, 14)	9 (7, 13)	9 (7, 15)	10 (7, 14)
Intellectual Disability	Psychiatric	19 (8, 38)	15 (7, 27)	13 (6, 23)	19 (8, 41)
OCD	Psychiatric	29 (21, 40)	30 (21.75, 40.25)	28 (21, 36)	30 (22, 41)
Personality Disorder	Psychiatric	30 (22, 41)	32 (24, 38)	32 (23, 40)	31 (23, 41)
Schizophrenia	Psychiatric	34 (25, 48)	31 (24, 40.25)	31 (24, 43)	35 (25, 50)
Substance Misuse	Psychiatric	28 (21, 39)	31 (23, 41)	29 (23, 39)	28 (22, 40)
Allergic/chronic Rhinitis	Respiratory	27 (13, 44)	27 (11, 39)	27 (12, 38)	29 (14, 48)
Asbestosis	Respiratory	70 (63, 78)	73 (68, 76)	66 (54, 72)	71 (64, 78)
Aspiration Pneumonitis	Respiratory	78 (62, 87)	66 (42, 79)	67 (35.25, 80)	79 (63, 87)
Asthma	Respiratory	19 (6, 42)	15 (5, 35)	18 (5, 37)	22 (7, 45)
Bronchiectasis	Respiratory	64 (48, 75)	50.5 (35, 67)	60 (40, 73)	65 (50, 76)
Chronic Sinusitis	Respiratory	42 (31, 55)	38 (30, 48)	36 (28, 46)	43 (32, 56)
COPD	Respiratory	66 (57, 76)	62 (48, 73)	66 (55, 75)	67 (58, 76)
Hypertroph Nasal	Respiratory	34 (21, 47)	28 (11, 38)	28 (19, 37)	35 (22, 48)

Turbs					
Nasal Polyps	Respiratory	46 (33, 58)	35 (24, 45)	35 (25, 46)	47 (34, 60)
Pleural Effusion	Respiratory	72 (57, 83)	47 (33, 67)	58 (35, 74)	73 (59, 83)
Pleural Plaque	Respiratory	73 (65, 81)	68 (60, 76.25)	67.5 (50.5, 74.5)	73 (66, 81)
Pneumothorax	Respiratory	31 (21, 56)	31 (21, 45)	29 (19, 54.75)	34 (22, 59)
Pulmonary Collapse	Respiratory	67 (50, 79)	49 (24, 66)	46 (25, 69.25)	68 (52, 79)
Pulmonary Fibrosis	Respiratory	74 (64, 82)	51 (39, 69.5)	69 (55, 78)	75 (65, 82)
Respiratory Failure	Respiratory	73 (61, 82)	53 (35.25, 74)	67 (45.25, 78)	73 (62, 83)
Sleep apnoea	Respiratory	48 (33, 59)	29 (4, 45)	38 (7, 51)	49 (36, 60)
Acne	Skin	18 (15, 26)	23 (16, 33)	21 (16, 29)	18 (15, 27)
Actinic keratosis	Skin	68 (59, 76)	58 (48, 69)	59 (49, 69)	69 (60, 77)
Alopecia Areata	Skin	30 (18, 42)	26 (14, 37)	29 (20, 36)	31 (19, 44)
Dermatitis	Skin	24 (3, 47)	9 (2, 33)	18 (2, 37)	28 (4, 51)
Hidradenitis	Skin	34 (26, 44)	33 (23, 41)	32 (25, 41)	35 (26, 44)
Lichen Planus	Skin	53 (39.5, 64)	40 (30, 51)	45 (34, 57)	55 (42, 65)
Pilonidal cyst/sinus	Skin	25 (20, 34)	29 (20, 38)	25 (20, 31)	26 (21, 35)
Psoriasis	Skin	37 (23, 54)	34 (22.75, 45)	35 (24, 49)	39 (24, 56)
Rosacea	Skin	45 (33, 58)	33 (21, 43)	34 (24, 44)	47 (35, 60)
Seborrheic Dermatitis	Skin	33 (13, 53)	11 (2, 33)	19 (2, 34)	37 (15, 58)
Urticaria	Skin	28 (8, 47)	27 (9, 41)	28 (9, 42)	30 (9, 50)
Vitiligo	Skin	33 (17, 48)	30 (12, 46)	26 (10, 42)	36 (21, 52)

Supplementary Table S8. Comparison of study design and characteristics between this study and the GBD[1] and Barnett[2] studies.

Study characteristics	Kuan	GBD[1]	Barnett[2]
**Number of diseases**	308	354	40
**Disease selection**	• diseases from the quality and outcomes framework (QOF)<br>• Hospital Episode Statistics Finished Consultant Episodes from all diagnoses for England > 10,000 from 1 April 2014 to 31 March 2015<br>• Period prevalence > 0.01% and clinically important	"reflect the policy relevance, public health, and medical care importance of the causes of major losses of health"	• morbidities recommended as core for any multimorbidity measure by Diederichs and colleagues' 2010 systematic review<br>• diseases in the quality and outcomes framework (QOF) of the UK general practice contract<br>• long-term disorders identified as important by NHS Scotland
**Source data**	Electronic Health Records from<br>• primary care<br>• secondary care<br>with linkage of individual records using unique NHS identification numbers	Various, including:<br>• published literature<br>• surveillance data<br>• survey data<br>• hospital and clinical data<br>• other data	Electronic Health Records from primary care only
**Geographical reach**	England	Results tabulated for 195 countries, comparison made with United Kingdom estimates	Scotland
**Disease frequency measure**	Period prevalence	Point prevalence	Point prevalence
**Calculation method for disease frequency**	Empirical frequency calculation:<br>Cases divided by relevant population	DisMod-MR 2.1, the GBD Bayesian meta-regression tool	Empirical frequency calculation:<br>Cases divided by relevant population
**Single disease prevalence stratified by sex**	Yes	Yes	No
**Single disease prevalence stratified by age**	Yes	Yes	No
**Single disease prevalence stratified by ethnicity**	Yes	No	No
**Age at single disease diagnosis**	Estimated by age at first record	No	No

Supplementary Table S9. Comparison of disease prevalences between this study, the Global Burden of Disease (GBD 2017)[1] study and the Barnett (2012)[2] study. Our study reports age and sex-standardised period prevalences in England from 1 April 2010 to 31 March 2015, whereas the GBD and Barnett studies computed point prevalences as at 2017 for the United Kingdom and 31 March 2007 for Scotland, respectively. Disease categories are arranged according to ICD-10 categories. Within disease categories, diseases are ranked in descending order of period prevalence from our study. Case definitions for some conditions may not be directly comparable – please refer to individual studies for details.

Disease Category	Disease label (Kuan 2018)	Prevalence per 10,000 (Kuan 2018)	Disease label (GBD 2017)[1]	Prevalence per 10,000 (GBD 2017)[1]	Disease label (Barnett 2012)[2]	Prevalence per 10,000 (Barnett 2012)[2]
**Infections**	Infection – Lower Respiratory Tract	623.8	Lower respiratory infections	4.4		
**Infections**	Urinary Tract Infection	439.2	Urinary tract infections	7.1		
**Infections**	Infection – Ear/Upper Respiratory Tract	381.5	Upper respiratory infections	423.9		
**Infections**	Tuberculosis	67.8	Tuberculosis	902.1		
**Infections**	Chronic Viral Hepatitis	24.3			Viral Hepatitis	10
**Infections**	Meningitis	11.2	Meningitis	4.9		
**Infections**	HIV	8.9	HIV/AIDS	14.2		
**Infections**	Encephalitis	2.6	Encephalitis	2.4		
**Benign/In situ Neoplasms**	Leiomyoma	401.2	Uterine fibroids	197.7		
**Benign/In situ Neoplasms**	Benign Neoplasm – Colon	289.1	Benign and in situ intestinal neoplasms	2.5		
**Cancers**	Primary Malignancy – Skin	311.6	Non-melanoma skin cancer	5.6		
**Cancers**	Primary Malignancy – Prostate	209.4	Prostate cancer	56.6		
**Cancers**	Primary Malignancy – Breast	138.5	Breast cancer	78.2		
**Cancers**	Primary Malignancy – Bowel	80.0	Colon and rectum cancer	43.2		
**Cancers**	Primary Malignancy – Melanoma	52.2	Malignant skin melanoma	27.7		
**Cancers**	Primary Malignancy – Bladder	46.5	Bladder cancer	9.5		
**Cancers**	Primary Malignancy – Lung	45.2	Tracheal, bronchus, and lung cancer	14.4		
**Cancers**	Primary Malignancy – Uterus	36.9	Uterine cancer	12.5		
**Cancers**	Non Hodgkins Lymphoma	29.6	Non-Hodgkin lymphoma	14.6		
**Cancers**	Primary Malignancy – Ovary	28.9	Ovarian cancer	4.9		
**Cancers**	Leukaemia	22.7	Leukemia	9.6		
**Cancers**	Primary Malignancy – Cervix	19.2	Cervical cancer	4.4		
**Cancers**	Primary Malignancy – Kidney	17.5	Kidney cancer	7.8		
**Cancers**	Primary Malignancy – Testis	16.2	Testicular cancer	3.4		
**Cancers**	Primary Malignancy – Oesophageal	13.0	Esophageal cancer	1.9		
**Cancers**	Primary Malignancy – Stomach	11.1	Stomach cancer	4.9		
**Cancers**	Plasma Cell Malignancy	10.4	Multiple myeloma	3.9		
**Cancers**	Primary Malignancy – Pancreas	9.9	Pancreatic cancer	1.2		
**Cancers**	Primary Malignancy – Brain	9.5	Brain and nervous system cancer	7.4		
**Cancers**	Hodgkins Lymphoma	7.2	Hodgkin lymphoma	3.7		
**Cancers**	Primary Malignancy – Thyroid	6.5	Thyroid cancer	4.1		
**Cancers**	Primary Malignancy – Liver	4.3	Liver cancer	0.4		
**Cancers**	Primary Malignancy – Biliary	4.0	Gallbladder and biliary tract cancer	0.5		
**Cancers**	Primary Malignancy – Mesothelioma	3.1	Mesothelioma	1.2		
**Haem/Imm**	Sickle Cell Trait	14.4	Sickle cell trait	408.8		
**Haem/Imm**	Thalassaemia Trait	14.0	Thalassemias trait	57.4		
**Haem/Imm**	Thalassaemia	5.5	Thalassemias	0.0		
**Haem/Imm**	Sickle Cell Disease	2.7	Sickle cell disorders	0.1		
**Endocrine**	Type 2 Diabetes Mellitus	599.6	Diabetes mellitus type 2	866.3		

**Endocrine**	Thyroid Disease	518.1			Thyroid disorders	410
**Endocrine**	Polycystic Ovarian Syndrome	90.1	Polycystic ovarian syndrome	64.5		
**Endocrine**	Type 1 Diabetes Mellitus	42.5	Diabetes mellitus type 1	42.8		
**Psychiatric**	Depression	1,744.3	Depressive disorders	435.7	Depression	820
**Psychiatric**	Anxiety	1,296.2	Anxiety disorders	443.7	Anxiety & other neurotic, stress related & somatoform disorders	320
**Psychiatric**	Alcohol Misuse	293.5	Alcohol use disorders	176.2	Alcohol problems	240
**Psychiatric**	Dementia	208.7	Alzheimer's disease and other dementias	122.7	Dementia	70
**Psychiatric**	Substance Misuse	159.3	Drug use disorders	143.3	Other psychoactive substance misuse	240
**Psychiatric**	Schizophrenia	75.4	Schizophrenia	28.8	Schizophrenia (and related non-organic psychosis) or bipolar disorder	70
**Psychiatric**	Intellectual Disability	64.5	Idiopathic developmental intellectual disability	32.8	Learning disability	30
**Psychiatric**	Bipolar Affective Disorder	45.1	Bipolar disorder	105.1		
**Psychiatric**	Autism	39.0	Autism spectrum disorders	59.2		
**Psychiatric**	Hyperkinetic Disorders	34.3	Attention-deficit/hyperactivity disorder	74.1		
**Psychiatric**	Eating Disorders	33.3	Eating disorders	43.8	Anorexia or bulimia	30
**Neurological**	Migraine	696.7	Migraine	2,064.6	Migraine	60
**Neurological**	Epilepsy	179.3	Epilepsy	38.7	Epilepsy (currently treated)	80
**Neurological**	Parkinson's Disease	47.1	Parkinson's disease	20.0	Parkinson's disease	20
**Neurological**	Multiple Sclerosis	26.2	Multiple sclerosis	14.6	Multiple sclerosis	20
**Neurological**	Motor Neurone Disease	4.5	Motor neuron disease	1.2		
**Eye**	Cataract	673.4	Cataract	60.2		
**Eye**	Glaucoma	182.4	Glaucoma	10.8	Glaucoma	90
**Eye**	Macular Degeneration	144.3	Age-related macular degeneration	23.0		
**Eye**	Blindness	120.3	Blindness and vision impairment	704.7	Blindness & low vision	50
**Ear**	Deafness	885.9	Age-related and other hearing loss	1,911.7	Hearing loss	340
**Cardiovascular**	Hypertension	2,062.7			Hypertension	1340
**Cardiovascular**	Atrial Fibrillation	432.9	Atrial fibrillation and flutter	140.3	Atrial fibrillation	140
**Cardiovascular**	Heart Failure	277.5			Heart failure	110
**Cardiovascular**	Peripheral Arterial Disease	162.1	Peripheral artery disease	275.9	Peripheral vascular disease	130
**Cardiovascular**	Ischaemic Stroke	134.2	Ischemic stroke	125.7		
**Cardiovascular**	Non-rheumatic Aortic valve disorder	98.6	Non-rheumatic calcific aortic valve disease	80.1		
**Cardiovascular**	Non-rheumatic Mitral valve disorder	84.0	Non-rheumatic degenerative mitral valve disease	45.9		
**Cardiovascular**	Intracerebral Haemorrhage	31.5	Intracerebral hemorrhage	13.7		
**Cardiovascular**	Rheumatic Valve Disorder	21.7	Rheumatic heart disease	0.6		
**Cardiovascular**	Subarachnoid Haemorrhage	20.7	Subarachnoid hemorrhage	19.6		
**Cardiovascular**	Dilated cardiomyopathy	14.7	Alcoholic cardiomyopathy	3.4		
**Respiratory**	Asthma	1,498.9	Asthma	837.1	Asthma	600
**Respiratory**	Chronic Obstructive Pulmonary Disease	371.5	Chronic obstructive pulmonary disease	818.9	Chronic obstructive pulmonary disease	320
**Respiratory**	Chronic Sinusitis	219.5			Chronic sinusitis	50
**Respiratory**	Bronchiectasis	57.3			Bronchiectasis	20
**Respiratory**	Pulmonary Fibrosis	28.5	Interstitial lung disease and pulmonary sarcoidosis	16.5		

**Respiratory**	Asbestosis	12.2	Asbestosis	0.3		
**Digestive**	Gastro-oesophageal Reflux Disease	941.6	Gastroesophageal reflux disease	1,281.4		
**Digestive**	Abdominal Hernia	757.2	Inguinal, femoral, and abdominal hernia	38.0		
**Digestive**	Gastritis	648.6	Gastritis and duodenitis	134.1		
**Digestive**	Irritable Bowel Syndrome	607.0			Irritable bowel syndrome	300
**Digestive**	Appendicitis	563.1	Appendicitis	0.3		
**Digestive**	Diverticular Disease	461.8			Diverticular disease of intestine	190
**Digestive**	Peptic Ulcer	246.9	Peptic ulcer disease	13.1		
**Digestive**	Pancreatitis	60.1	Pancreatitis	20.6		
**Digestive**	Angiodysplasia of colon	8.5	Vascular intestinal disorders	0.3		
**Skin**	Dermatitis	2,543.5	Dermatitis	682.3		
**Skin**	Acne	734.4	Acne vulgaris	319.7		
**Skin**	Urticaria	520.2	Urticaria	72.3		
**Skin**	Seborrheic Dermatitis	498.4	Seborrhoeic dermatitis	8.9		
**Skin**	Psoriasis	348.1	Psoriasis	214.3		
**Skin**	Alopecia Areata	38.0	Alopecia areata	32.2		
**Musculoskeletal**	Osteoarthritis	1,271.6	Osteoarthritis	730.5		
**Musculoskeletal**	Gout	318.3	Gout	123.7		
**Musculoskeletal**	Rheumatoid Arthritis	115.4	Rheumatoid arthritis	69.7		
**Genitourinary**	Benign Prostatic Hyperplasia	769.4	Benign prostatic hyperplasia	171.8	Prostate disorders	90
**Genitourinary**	Uterovaginal Prolapse	537.2	Genital prolapse	259.3		
**Genitourinary**	Chronic Kidney Disease	427.4	Chronic kidney disease	845.9	Chronic kidney disease	190
**Genitourinary**	Female Infertility	251.5	Female infertility	27.2		
**Genitourinary**	Urolithiasis	239.9	Urolithiasis	5.9		
**Genitourinary**	Endometriosis	228.3	Endometriosis	86.0		
**Genitourinary**	Glomerulonephritis	68.9	Acute glomerulonephritis	0.0		
**Genitourinary**	Male infertility	62.3	Male infertility	27.2		
**Perinatal/Congenital**	Neonatal Jaundice	91.8	Hemolytic disease and other neonatal jaundice	0.8		
**Perinatal/Congenital**	Premature Delivery	69.3	Neonatal preterm birth	102.7		
**Perinatal/Congenital**	Congenital Septal Defect	36.5	Congenital heart anomalies	12.3		
**Perinatal/Congenital**	Sepsis of the Newborn	15.8	Neonatal sepsis and other neonatal infections	30.8		
**Perinatal/Congenital**	Spina Bifida	11.6	Neural tube defects	2.5		
**Perinatal/Congenital**	Down Syndrome	7.1	Down syndrome	6.2		

Supplementary Table S10A. Infectious diseases and their prevalences (ranked in descending order) from the Kuan 2018, GBD 2017[1], and Barnett 2012[2] studies which are not reported in any of the other studies.

Disease label (Kuan 2018)	Prevalence per 10,000 (Kuan 2018)	Disease label (GBD 2017)	Prevalence per 10,000 (GBD 2017)	Disease label (Barnett 2012)	Prevalence per 10,000 (Barnett 2012)
Bacterial Infection	1,008	Respiratory infections and tuberculosis	1375		
Infection – Other organisms	946	HIV/AIDS and sexually transmitted infections	1320		
Infection – Other organs	330	Sexually transmitted infections excluding HIV	1309		
Viral Infection	327	Fungal skin diseases	1107		
Infection – Skin	295	Genital herpes	1079		
Infection – Digestive System	250	Latent tuberculosis infection	902		
Pelvic Inflammatory Disease	183	Viral skin diseases	147		
Septicaemia	127	Trichomoniasis	110		
Fungal Infection	75	Chlamydial infection	101		
Infection – Male Genitourinary	64	Otitis media	98		
Rheumatic Fever	37	Diarrheal diseases	56		
Infection – Anorectal	36	Enteric infections	56		
Infection – Bone	29	Other infectious diseases	54		
Infection – Eye	27	Gonococcal infection	35		
Infection – Other Genitourianry	23	Other unspecified infectious diseases	34		
Infection – Liver	21	Bacterial skin diseases	26		
Parasitic Infection	12	Syphilis	22		
Infection – Other nervous system	11	Pyoderma	16		
Infection – Heart	6	Acute hepatitis	12		
		Other sexually transmitted infections	10		
		Scabies	10		
		Cellulitis	10		
		Cysticercosis	9		
		Neglected tropical diseases and malaria	9		
		Varicella and herpes zoster	9		
		Acute hepatitis A	7		
		Acute hepatitis B	4		
		Other meningitis	3		
		Whooping cough	1		
		Meningococcal meningitis	1		
		Pneumococcal meningitis	1		
		Acute hepatitis E	1		
		Drug-susceptible tuberculosis	1		
		Acute hepatitis C	0		
		H influenzae type B meningitis	0		
		Chagas disease	0		

HIV/AIDS - Drug-susceptible Tuberculosis	0
Multidrug-resistant tuberculosis without extensive drug resistance	0
Invasive Non-typhoidal Salmonella (iNTS)	0
Cystic echinococcosis	0
Paratyphoid fever	0
Typhoid fever	0
Extensively drug-resistant tuberculosis	0
HIV/AIDS - Multidrug-resistant Tuberculosis without extensive drug resistance	0
Measles	0
HIV/AIDS - Extensively drug-resistant Tuberculosis	0
Leprosy	0
Tetanus	0
Diphtheria	0
Rabies	0
Other intestinal infectious diseases	0
Malaria	0
Leishmaniasis	0
Visceral leishmaniasis	0
Cutaneous and mucocutaneous leishmaniasis	0
African trypanosomiasis	0
Schistosomiasis	0
Lymphatic filariasis	0
Onchocerciasis	0
Trachoma	0
Dengue	0
Yellow fever	0
Intestinal nematode infections	0
Ascariasis	0
Trichuriasis	0
Hookworm disease	0
Food-borne trematodiases	0
Other neglected tropical diseases	0
Ebola	0
Zika virus	0
Guinea worm disease	0
Typhoid and paratyphoid	0

Supplementary Table S10B. Neoplastic diseases and their prevalences (ranked in descending order) from the Kuan 2018, GBD 2017[1], and Barnett 2012[2] studies which are not reported in any of the other studies.

Disease label (Kuan 2018)	Prevalence per 10,000 (Kuan 2018)	Disease label (GBD 2017)	Prevalence per 10,000 (GBD 2017)	Disease label (Barnett 2012)	Prevalence per 10,000 (Barnett 2012)
Benign Neoplasm – Ovary	383	Neoplasms	394	New diagnosis of cancer in last five years	250
Cervical Intra-epithelial Neoplasia	241	Other malignant neoplasms	68		
Benign Neoplasm – Uterus	181	Other neoplasms	25		
Haemangioma	127	Other benign and in situ neoplasms	19		
Secondary Malignancy – Lymph Nodes	65	Chronic lymphoid leukemia	6		
Benign Neoplasm – Stomach	56	Non-melanoma skin cancer (squamous-cell carcinoma)	4		
Secondary Malignancy – Liver	40	Lip and oral cavity cancer	4		
Secondary Malignancy – Bone	36	Larynx cancer	3		
Primary Malignancy – other	33	Non-melanoma skin cancer (basal-cell carcinoma)	2		
Secondary Malignancy – Lung	28	Other pharynx cancer	2		
Benign Neoplasm – Brain	25	Acute lymphoid leukemia	1		
Secondary Malignancy – other	18	Other leukemia	1		
Primary Malignancy – Oropharyngeal	17	Acute myeloid leukemia	1		
Secondary Malignancy – Peritoneum	15	Benign and in situ cervical and uterine neoplasms	1		
Monoclonal Gammopathy of Undetermined Significance	12	Liver cancer due to alcohol use	1		
Myelodysplastic Syndrome	11	Liver cancer due to hepatitis C	0		
Polycythaemia vera	11	Nasopharynx cancer	0		
Secondary Malignancy – Brain	11	Chronic myeloid leukemia	0		
Secondary Malignancy – Pleura	7	Liver cancer due to hepatitis B	0		
Secondary Malignancy – Adrenal	5	Liver cancer due to NASH	0		
Primary Malignancy – Bone	4	Liver cancer due to other causes	0		
Secondary Malignancy – Bowel	3				
Primary Malignancy – Multiple Sites	2				

Supplementary Table S10C. Haematological and Immunological diseases and their prevalences (ranked in descending order) from the Kuan 2018, GBD 2017[1], and Barnett 2012[2] studies which are not reported in any of the other studies.

Disease label (Kuan 2018)	Prevalence per 10,000 (Kuan 2018)	Disease label (GBD 2017)	Prevalence per 10,000 (GBD 2017)	Disease label (Barnett 2012)	Prevalence per 10,000 (Barnett 2012)
Anaemia – other	511	Hemoglobinopathies and hemolytic anemias	1564		
Iron Deficiency Anaemia	433	G6PD trait	944		
Agranulocytosis	60	G6PD deficiency	177		
Vitamin B12 deficiency anaemia	50	Other hemoglobinopathies and hemolytic anemias	50		
Secondary Thrombocytopaenia	44				
Sarcoidosis	20				
Folate Deficiency Anaemia	16				
Primary thrombocytopaenia	15				
Hyposplenism	15				
Hypersplenism	15				
Aplastic Anaemia	12				
Thrombophilia	12				
Other haemolytic anaemia	12				
Secondary Polycythaemia	10				
Immunodeficiency	6				

Supplementary Table S10D. Endocrine, metabolic and nutritional diseases and their prevalences (ranked in descending order) from the Kuan 2018, GBD 2017[1], and Barnett 2012[2] studies which are not reported in any of the other studies.

Disease label (Kuan 2018)	Prevalence per 10,000 (Kuan 2018)	Disease label (GBD 2017)	Prevalence per 10,000 (GBD 2017)	Disease label (Barnett 2012)	Prevalence per 10,000 (Barnett 2012)
Raised Total Cholesterol	2398	Nutritional deficiencies	1042	Diabetes	430
Obesity	1692	Diabetes mellitus	909		
Raised LDL-C	1531	Dietary iron deficiency	732		
Raised Triglycerides	1123	Vitamin A deficiency	196		
Low HDL-C	673	Iodine deficiency	111		
Diabetes Mellitus – other or not specified	35	Protein-energy malnutrition	60		
Hyperparathyroidism	22	Other nutritional deficiencies	0		
Syndrome of Inappropriate AntiDiuretic Hormone	6				
Cystic Fibrosis	5				

Supplementary Table S10E. Mental and behavioural disorders and their prevalences (ranked in descending order) from the Kuan 2018, GBD 2017[1], and Barnett 2012[2] studies which are not reported in any of the other studies.

Disease label (Kuan 2018)	Prevalence per 10,000 (Kuan 2018)	Disease label (GBD 2017)	Prevalence per 10,000 (GBD 2017)	Disease label (Barnett 2012)	Prevalence per 10,000 (Barnett 2012)
Personality Disorder	63	Mental disorders	1404		
Obsessive Compulsive Disorder	44	Substance use disorders	313		
Delirium	58	Major depressive disorder	272		
		Other mental disorders	231		
		Dysthymia	173		
		Conduct disorder	51		
		Opioid use disorders	43		
		Cannabis use disorders	41		
		Bulimia nervosa	34		
		Cocaine use disorders	32		
		Amphetamine use disorders	23		
		Cannabis use disorders	14		
		Anorexia nervosa	10		
		Other drug use disorders	7		

Supplementary Table S10F. Neurological diseases and their prevalences (ranked in descending order) from the Kuan 2018, GBD 2017[1], and Barnett 2012[2] studies which are not reported in any of the other studies.

Disease label (Kuan 2018)	Prevalence per 10,000 (Kuan 2018)	Disease label (GBD 2017)	Prevalence per 10,000 (GBD 2017)	Disease label (Barnett 2012)	Prevalence per 10,000 (Barnett 2012)
Peripheral Neuropathy	218	Neurological disorders	4769		
Chronic Fatigue Syndrome	148	Headache disorders	4661		
Bell's Palsy	76	Tension-type headache	3613		
Trigeminal Neuralgia	55	Other neurological disorders	0		
Diabetic Neuropathy	41				
Essential Tremor	26				
Autonomic Neuropathy	26				
Cerebral Palsy	16				
Idiopathic Intracranial Hypertension	5				
Myasthenia Gravis	4				

Supplementary Table S10G. Eye and ear diseases and their prevalences (ranked in descending order) from the Kuan 2018, GBD 2017[1], and Barnett 2012[2] studies which are not reported in any of the other studies.

Disease label (Kuan 2018)	Prevalence per 10,000 (Kuan 2018)	Disease label (GBD 2017)	Prevalence per 10,000 (GBD 2017)	Disease label (Barnett 2012)	Prevalence per 10,000 (Barnett 2012)
Tinnitus	322	Near vision loss	378		
Diabetic Eye Disease	203	Refraction disorders	221		
Anterior Uveitis	71	Other vision loss	46		
Reitnal Detachment	58				
Keratitis	51				
Scleritis	46				
Retinal Vascular Occlusion	42				
Ptosis	39				
Meniere's Disease	37				
Posterior Uveitis	6				

Supplementary Table S10H. Cardiovascular diseases and their prevalences (ranked in descending order) from the Kuan 2018, GBD 2017[1], and Barnett 2012[2] studies which are not reported in any of the other studies.

Disease label (Kuan 2018)	Prevalence per 10,000 (Kuan 2018)	Disease label (GBD 2017)	Prevalence per 10,000 (GBD 2017)	Disease label (Barnett 2012)	Prevalence per 10,000 (Barnett 2012)
Stable Angina	455	Cardiovascular diseases	1077	Coronary heart disease	470
Myocardial Infarction	299	Ischemic heart disease	334	Stroke & transient ischaemic attack	210
Transient Ischaemic Attack	190	Other cardiovascular and circulatory diseases	249		
Unstable Angina	159	Stroke	152		
Venous thrombolism (Excl PE)	153	Non-rheumatic valvular heart disease	123		
Stroke – not otherwise specified	112	Hypertensive heart disease	15		
Coronary Heart Disease (not otherwise specified)	107	Cardiomyopathy and myocarditis	11		
Raynauds Disease	102	Other cardiomyopathy	8		
Pulmonary Embolism	100	Myocarditis	3		
Supraventricular Tachycardia	75	Endocarditis	2		
Multiple valve disorder	58	Other non-rheumatic valve diseases	0		
Left Bundle Branch Block	55				
Right Bundle Branch Block	55				
Abdominal Aortic Aneurysm	54				
Atrioventricular Block, first degree	35				
Cardiomyopathy – other	26				
Ventricular Tachycardia	20				
Atrioventricular Block, third degree	20				
Primary Pulmonary Hypertension	19				
Pericardial Effusion	16				
Atrioventricular Block, second degree	13				
Sick Sinus Syndrome	13				
Subdural haematoma	12				
Secondary Pulmonary Hypertension	11				
Hypertrophic cardiomyopathy	6				
Trifascicular Block	5				
Bifascicular Block	4				

Supplementary Table S10I. Respiratory diseases and their prevalences (ranked in descending order) from the Kuan 2018, GBD 2017[1], and Barnett 2012[2] studies which are not reported in any of the other studies.

Disease label (Kuan 2018)	Prevalence per 10,000 (Kuan 2018)	Disease label (GBD 2017)	Prevalence per 10,000 (GBD 2017)	Disease label (Barnett 2012)	Prevalence per 10,000 (Barnett 2012)
Allergic/chronic Rhinitis	1493	Chronic respiratory diseases	1536		
Pleural Effusion	164	Pneumoconiosis	1		
Nasal Polyps	109	Other pneumoconiosis	0		
Sleep apnoea	101	Coal workers pneumoconiosis	0		
Respiratory Failure	82	Silicosis	0		
Pulmonary Collapse	56	Other chronic respiratory diseases	0		
Pneumothorax	48				
Aspiration Pneumonitis	41				
Hypertrophic Nasal Turbinates	36				
Pleural Plaque	22				

Supplementary Table S10J. Oral diseases and their prevalences (ranked in descending order) from the Kuan 2018, GBD 2017[1], and Barnett 2012[2] studies which are not reported in any of the other studies.

Disease label (Kuan 2018)	Prevalence per 10,000 (Kuan 2018)	Disease label (GBD 2017)	Prevalence per 10,000 (GBD 2017)	Disease label (Barnett 2012)	Prevalence per 10,000 (Barnett 2012)
		Oral disorders	4781		
		Caries of permanent teeth	3679		
		Periodontal diseases	1082		
		Edentulism and severe tooth loss	631		
		Caries of deciduous teeth	237		
		Other oral disorders	197		

Supplementary Table S10K. Digestive diseases and their prevalences (ranked in descending order) from the Kuan 2018, GBD 2017[1], and Barnett 2012[2] studies which are not reported in any of the other studies.

Disease label (Kuan 2018)	Prevalence per 10,000 (Kuan 2018)	Disease label (GBD 2017)	Prevalence per 10,000 (GBD 2017)	Disease label (Barnett 2012)	Prevalence per 10,000 (Barnett 2012)
Oesophageal Ulcer	585	Digestive diseases	2576	Treated dyspepsia	450
Diaphragmatic Hernia	498	Cirrhosis and other chronic liver diseases	1407	Treated constipation	220
Cholelithiasis	344	Upper digestive system diseases	1325	Inflammatory bowel disease	60
Anal Fissure	238	Cirrhosis due to NASH	1132	Chronic liver disease	10
Cholecystitis	172	Cirrhosis and other chronic liver diseases due to hepatitis B	94		
Ulcerative Colitis	66	Cirrhosis and other chronic liver diseases due to alcohol use	75		
Peritonitis	66	Gallbladder and biliary diseases	72		
Barrett's Oesophagus	53	Cirrhosis and other chronic liver diseases due to hepatitis C	59		
Anorectal Fistula	46	Inflammatory bowel disease	54		
Crohns Disease	41	Cirrhosis and other chronic liver diseases due to other causes	48		
Fatty Liver	40	Paralytic ileus and intestinal obstruction	0		
Coeliac Disease	34	Other digestive diseases	0		
Cirrhosis	34				
Alcoholic Liver Disease	33				
Anorectal Prolapse	33				
Cholangitis	19				
Liver Failure	14				
Oesophageal Varices	13				
Portal Hypertension	12				
Volvulus	12				
Autoimmune liver disease	7				

Supplementary Table S10L. Skin diseases and their prevalences (ranked in descending order) from the Kuan 2018, GBD 2017[1], and Barnett 2012[2] studies which are not reported in any of the other studies.

Disease label (Kuan 2018)	Prevalence per 10,000 (Kuan 2018)	Disease label (GBD 2017)	Prevalence per 10,000 (GBD 2017)	Disease label (Barnett 2012)	Prevalence per 10,000 (Barnett 2012)
Actinic keratosis	273	Skin and subcutaneous diseases	3290	Psoriasis or eczema	70
Rosacea	250	Other skin and subcutaneous diseases	1233		
Pilonidal cyst/sinus	88	Atopic dermatitis	557		
Lichen Planus	62	Contact dermatitis	127		
Vitiligo	36	Pruritus	94		
Hidradenitis	27	Decubitus ulcer	4		

Supplementary Table S10M. Musculoskeletal diseases and their prevalences (ranked in descending order) from the Kuan 2018, GBD 2017[1], and Barnett 2012[2] studies which are not reported in any of the other studies.

Disease label (Kuan 2018)	Prevalence per 10,000 (Kuan 2018)	Disease label (GBD 2017)	Prevalence per 10,000 (GBD 2017)	Disease label (Barnett 2012)	Prevalence per 10,000 (Barnett 2012)
Enthesopathy	1854	Musculoskeletal disorders	2821	Painful condition	720
Spondylosis	458	Low back pain	1568	Rheumatoid arthritis, other inflammatory polyarthropathies & systematic connective tissue disorders	340
Carpal Tunnel Syndrome	370	Neck pain	681		
Fracture – Wrist	320	Other musculoskeletal disorders	419		
Intervertebral Disc Disorder	315				
Osteoporosis	303				
Fracture – Hip	123				
Fibromatosis	112				
Polymyalgia Rheumatica	90				
Spinal Stenosis	80				
Scoliosis	78				
Spondylolisthesis	36				
Collapsed Vertebra	29				
Psoriatic Arthritis	24				
Ankylosing Spondylosis	19				
Giant Cell Arteritis	18				
Lupus Erythematosus	16				
Sjogren Syndrome	11				
Reactive Arthritis	11				
Juvenile Arthritis	6				
Scleroderma	4				
Enteropathic Arthropathy	1				

Supplementary Table S10N. Genitourinary diseases and their prevalences (ranked in descending order) from the Kuan 2018, GBD 2017[1], and Barnett 2012[2] studies which are not reported in any of the other studies.

Disease label (Kuan 2018)	Prevalence per 10,000 (Kuan 2018)	Disease label (GBD 2017)	Prevalence per 10,000 (GBD 2017)	Disease label (Barnett 2012)	Prevalence per 10,000 (Barnett 2012)
Menorrhagia	1377	Gynecological diseases	1068		
Erectile Dysfunction	916	Chronic kidney disease due to other and unspecified causes	623		
Dysmenorrhoea	597	Premenstrual syndrome	497		
Urinary Incontinence	400	Urinary diseases and male infertility	206		
Postmenopausal Bleeding	395	Chronic kidney disease due to diabetes mellitus type 2	142		
Postcoital Bleeding	258	Other gynecological diseases	94		
Hydrocele	204	Chronic kidney disease due to glomerulonephritis	49		
Acute Kidney Injury	200	Chronic kidney disease due to hypertension	26		
Neuropahtic Bladder	100	Chronic kidney disease due to diabetes mellitus type 1	5		
Undescended Testis	89	Other urinary diseases	0		
Obstructive and reflux uropathy	87				
Endometrial Hyperplasia	72				
Tubulo-interstitial Nephropathy	32				
End Stage Renal Disease	29				
Chronic Cystitis	21				

Supplementary Table S10O. Maternal disorders and their prevalences (ranked in descending order) from the Kuan 2018, GBD 2017[1], and Barnett 2012[2] studies which are not reported in any of the other studies.

Disease label (Kuan 2018)	Prevalence per 10,000 (Kuan 2018)	Disease label (GBD 2017)	Prevalence per 10,000 (GBD 2017)	Disease label (Barnett 2012)	Prevalence per 10,000 (Barnett 2012)
		Maternal disorders	8		
		Maternal hypertensive disorders	5		
		Maternal sepsis and other maternal infections	1		
		Maternal hemorrhage	1		
		Maternal obstructed labor and uterine rupture	0		
		Maternal abortion and miscarriage	0		
		Ectopic pregnancy	0		
		Other maternal disorders	0		

Supplementary Table S10P. Perinatal and congenital disorders and their prevalences (ranked in descending order) from the Kuan 2018, GBD 2017[1], and Barnett 2012[2] studies which are not reported in any of the other studies.

Disease label (Kuan 2018)	Prevalence per 10,000 (Kuan 2018)	Disease label (GBD 2017)	Prevalence per 10,000 (GBD 2017)	Disease label (Barnett 2012)	Prevalence per 10,000 (Barnett 2012)
Low Birth Weight	56	Congenital birth defects	186		
Intrauterine Hypoxia	47	Neonatal disorders	152		
Respiratory Distress of the Newborn	42	Digestive congenital anomalies	77		
High Birth Weight	10	Congenital musculoskeletal and limb anomalies	51		
Patent Ductus Arteriosus	10	Neonatal encephalopathy due to birth asphyxia and trauma	32		
Post-term Delivery	8	Other congenital birth defects	23		
		Orofacial clefts	14		
		Other chromosomal abnormalities	13		
		Urogenital congenital anomalies	10		
		Turner syndrome	1		
		Klinefelter syndrome	0		
		Other neonatal disorders	0		

Supplementary Table S10Q. Injuries and their prevalences (ranked in descending order) from the Kuan 2018, GBD 2017[1], and Barnett 2012[2] studies which are not reported in any of the other studies.

Disease label (Kuan 2018)	Prevalence per 10,000 (Kuan 2018)	Disease label (GBD 2017)	Prevalence per 10,000 (GBD 2017)	Disease label (Barnett 2012)	Prevalence per 10,000 (Barnett 2012)
		Injuries	3000		
		Unintentional injuries	2469		
		Falls	1237		
		Other unintentional injuries	556		
		Exposure to mechanical forces	444		
		Other exposure to mechanical forces	435		
		Transport injuries	297		
		Road injuries	257		
		Self-harm and interpersonal violence	235		
		Interpersonal violence	193		
		Fire, heat, and hot substances	145		
		Sexual violence	136		
		Motor vehicle road injuries	78		
		Cyclist road injuries	70		
		Motorcyclist road injuries	50		
		Pedestrian road injuries	44		
		Physical violence by other means	43		
		Other transport injuries	40		
		Self-harm	35		
		Self-harm by other specified means	35		
		Foreign body	25		
		Animal contact	25		
		Environmental heat and cold exposure	20		
		Non-venomous animal contact	18		
		Foreign body in other body part	17		
		Other road injuries	16		
		Poisonings	14		
		Poisoning by other means	12		
		Physical violence by sharp object	11		
		Unintentional firearm injuries	9		
		Venomous animal contact	7		
		Conflict and terrorism	6		
		Pulmonary aspiration and foreign body in airway	5		
		Foreign body in eyes	3		
		Physical violence by firearm	3		
		Poisoning by carbon monoxide	2		
		Adverse effects of medical treatment	2		
		Drowning	2		
		Executions and police conflict	1		
		Exposure to forces of nature	0		
		Self-harm by firearm	0		

Supplementary Table S10R. General causes spanning several categories and their prevalences (ranked in descending order) from the Kuan 2018, GBD 2017[1], and Barnett 2012[2] studies which are not reported in any of the other studies.

Disease label (Kuan 2018)	Prevalence per 10,000 (Kuan 2018)	Disease label (GBD 2017)	Prevalence per 10,000 (GBD 2017)	Disease label (Barnett 2012)	Prevalence per 10,000 (Barnett 2012)
		Non-communicable diseases	9213		
		Other non-communicable diseases	6170		
		Communicable, maternal, neonatal, and nutritional diseases	3463		
		Sense organ diseases	2239		
		Diabetes and kidney diseases	1499		
		Other sense organ diseases	192		
		Maternal and neonatal disorders	159		
		Endocrine, metabolic, blood, and immune disorders	137		
		Myelodysplastic, myeloproliferative, and other hematopoietic neoplasms	4		

Supplementary Figure S1. Criteria for inclusion of health conditions in this analysis were based on FCE's, prevalence and clinical importance. FCE's were obtained from NHS Digital hospital admitted patient care activity data for England between 1 April 2014 and 31 March 2015. Abbreviations: FCE: Finished consultant episodes; ICD-10: International Classification of Diseases, tenth revision.

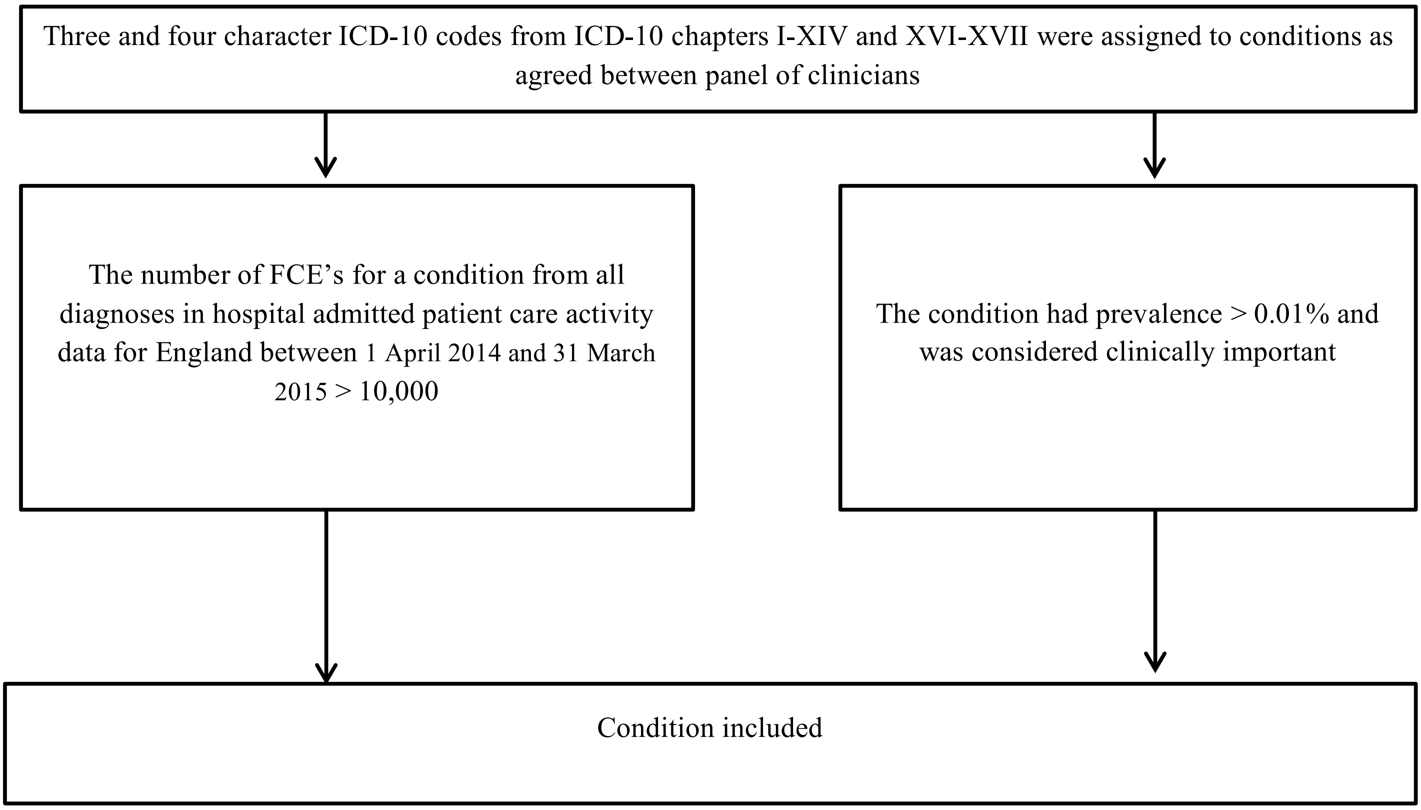

Supplementary Figure S2. Scatter plot of prevalence estimates between A) GBD[1] and Kuan studies where the prevalence in both studies was < 5%, B) Barnett[2] and Kuan studies where the prevalence in both studies was < 5%, C) GBD[1] and Kuan studies where the prevalence in at least one of the studies was > 5%, and D) Barnett[2] and Kuan studies where the prevalence in at least one of the studies was > 5%. The Kuan study used period prevalence, while the GBD[1] and Barnett[2] studies used point prevalence estimates.

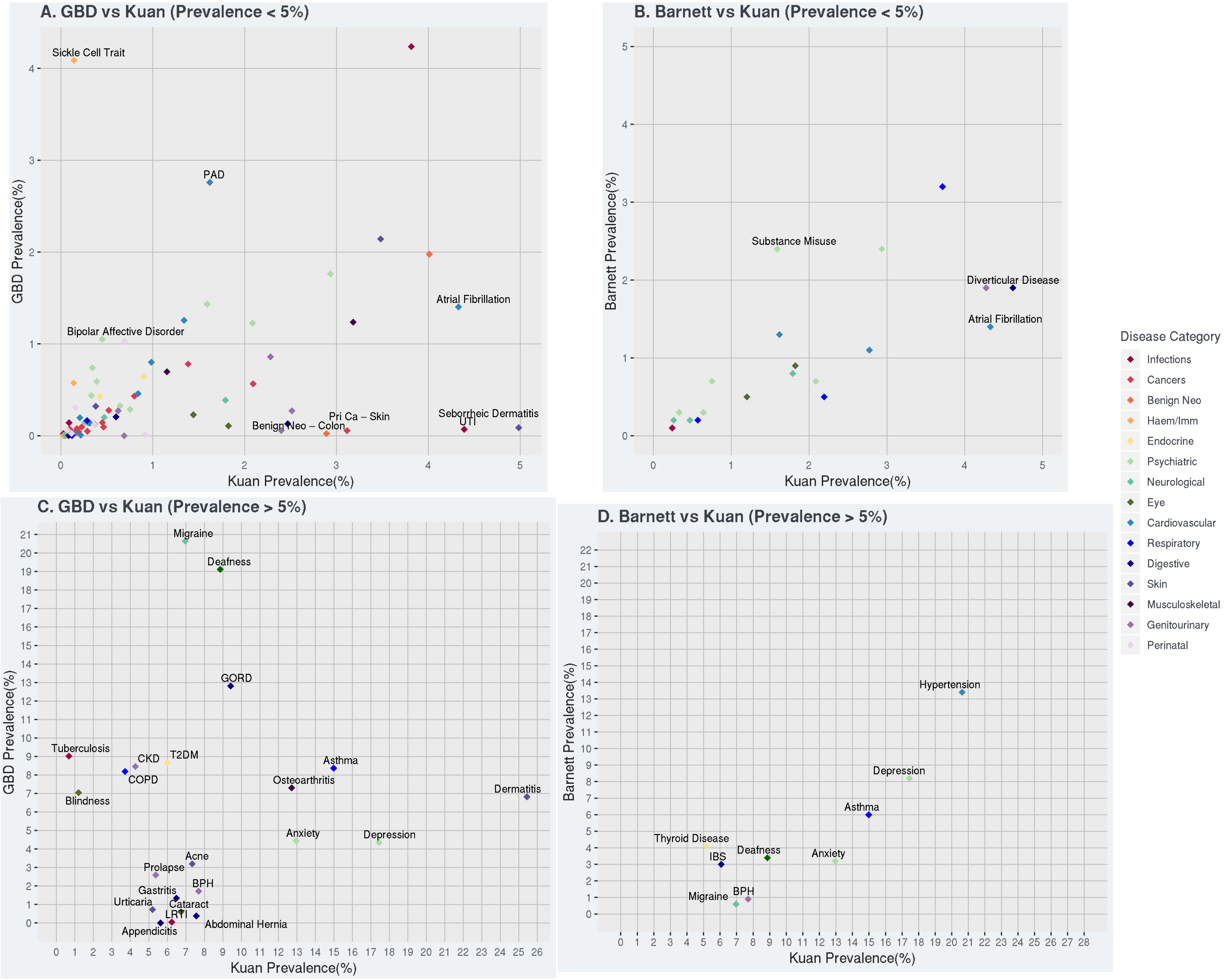

Supplementary Figure S3. Sex-standardised period prevalence (%) of the top 50 diseases and all cancers between 1 April 2010 and 31 March 2015 for individuals aged A) 1-9+ years, B) 10-19+ years, C) aged 20-29+ years, D) aged 30-39+ years, E) aged 40-49+ years, F) aged 50-59+ years, G) aged 60-69+ years, H) aged 70-79+ years, I) aged 80+ years on 1 April 2010

A. 1-9+y **B.** 10-19+y **C.** 20-29+y

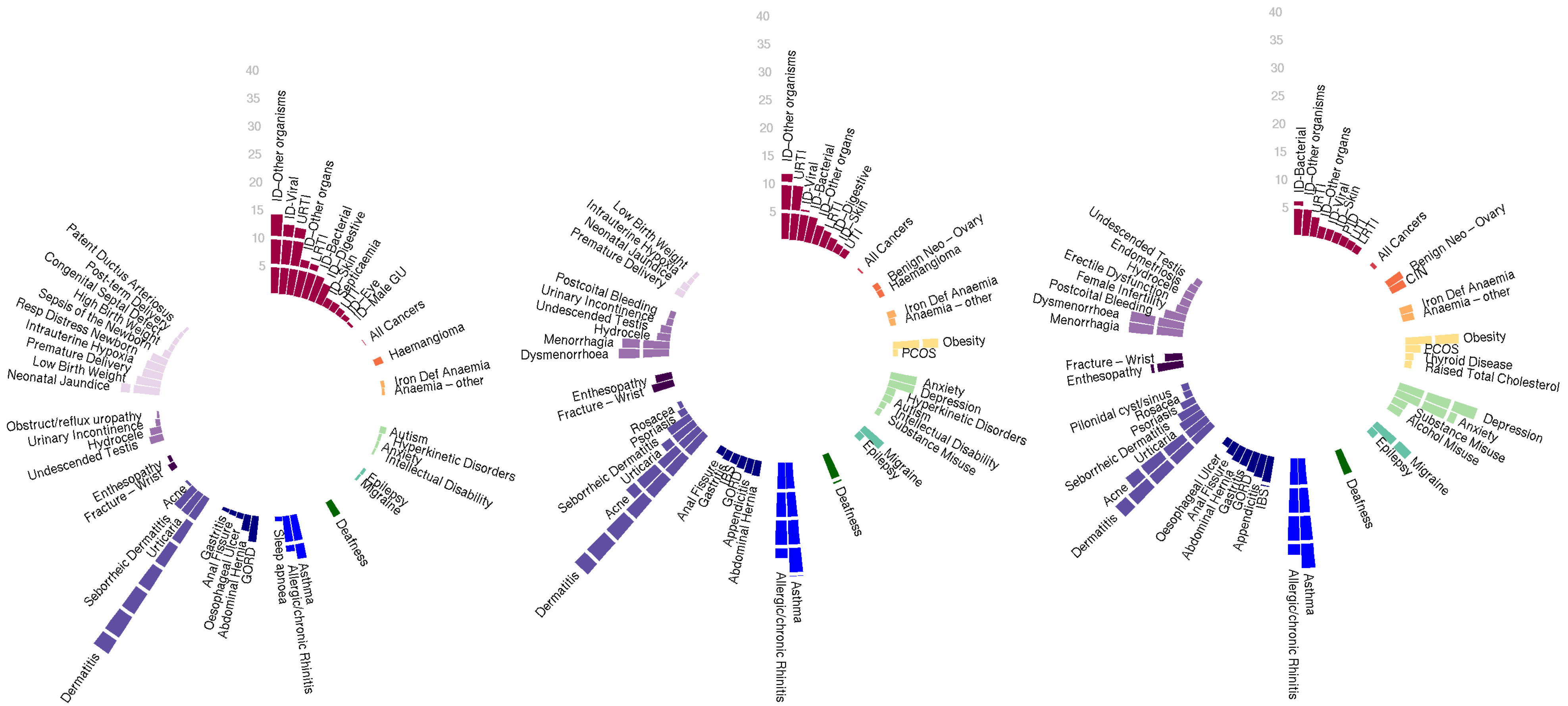

D. 30-39+y

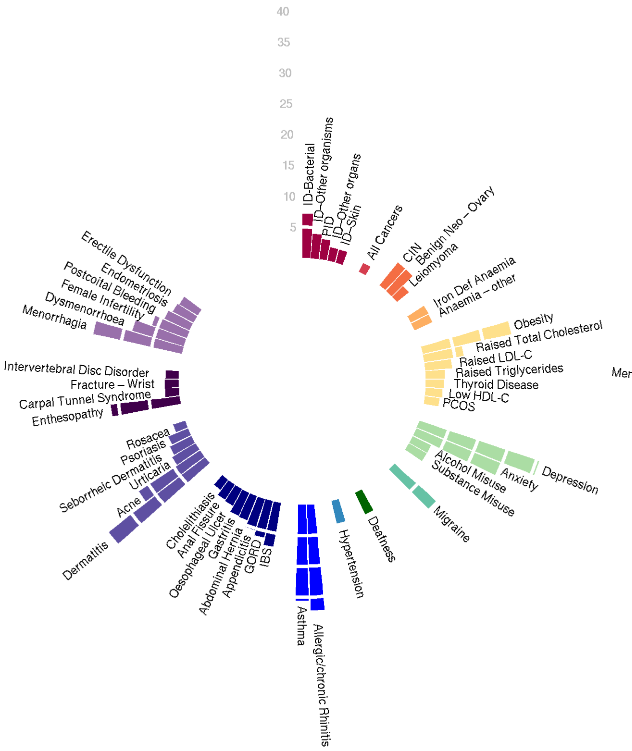

E. 40-49+y

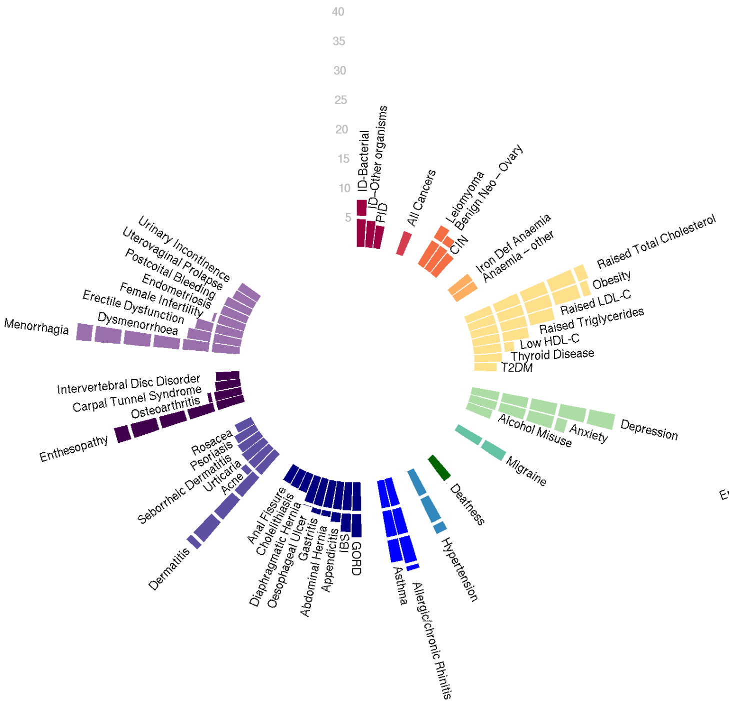

F. 50-59+y

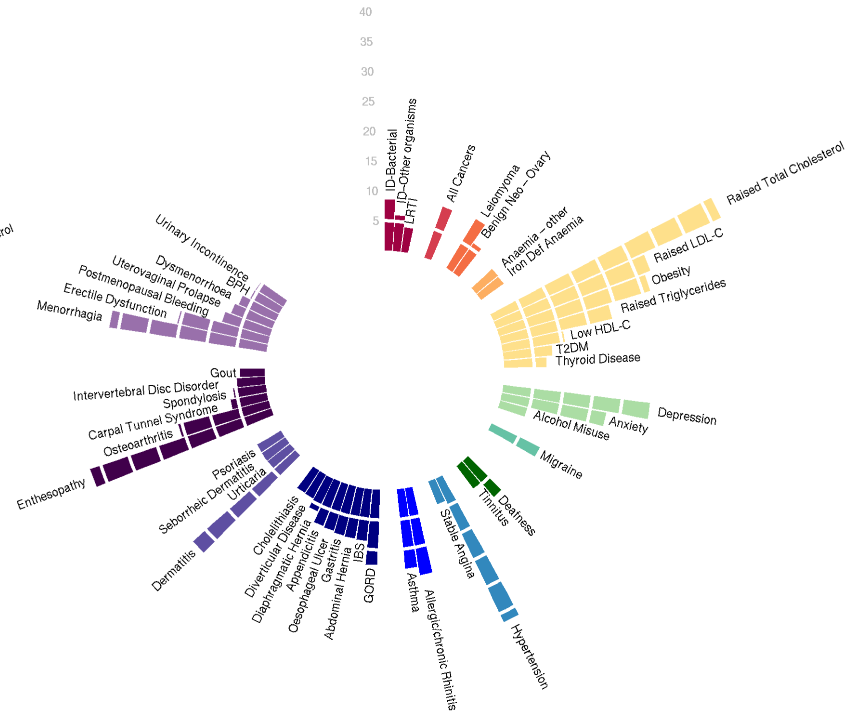

G. 60-69+y

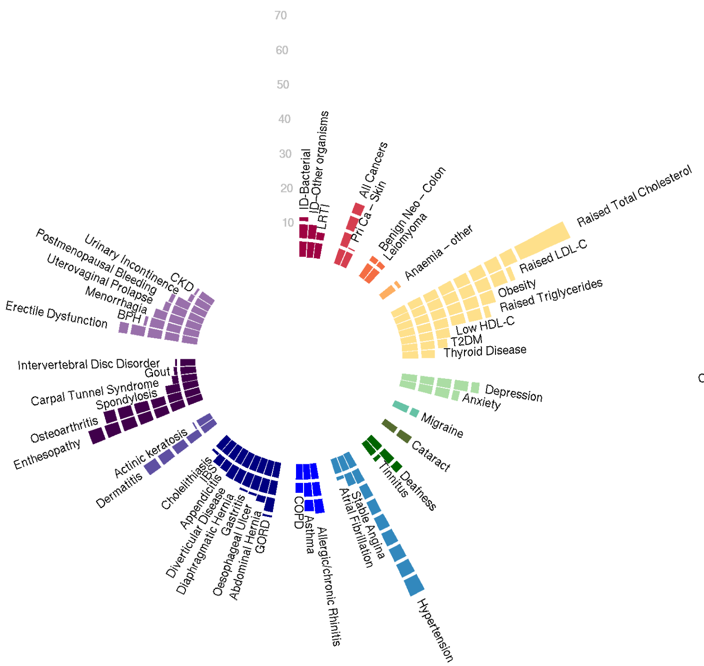

H. 70-79+y

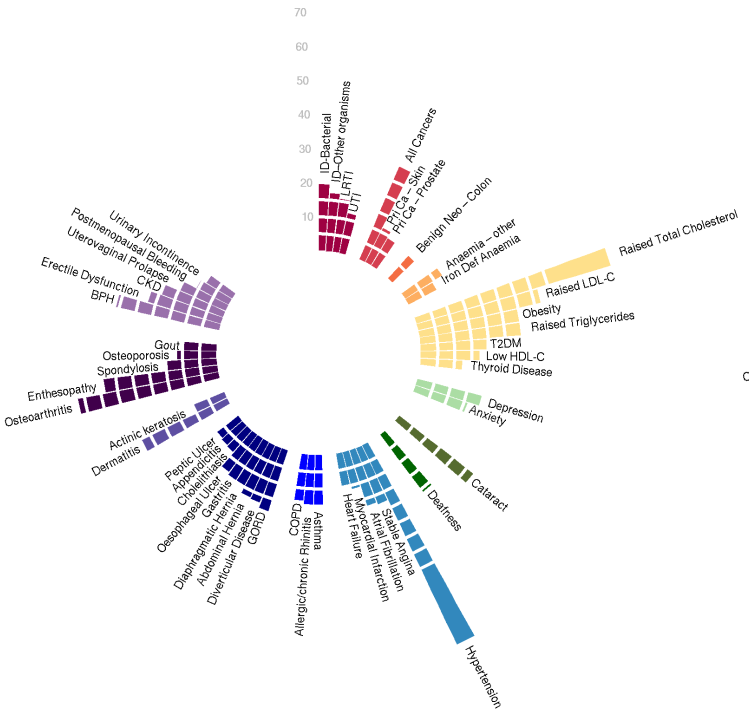

I. 80+y

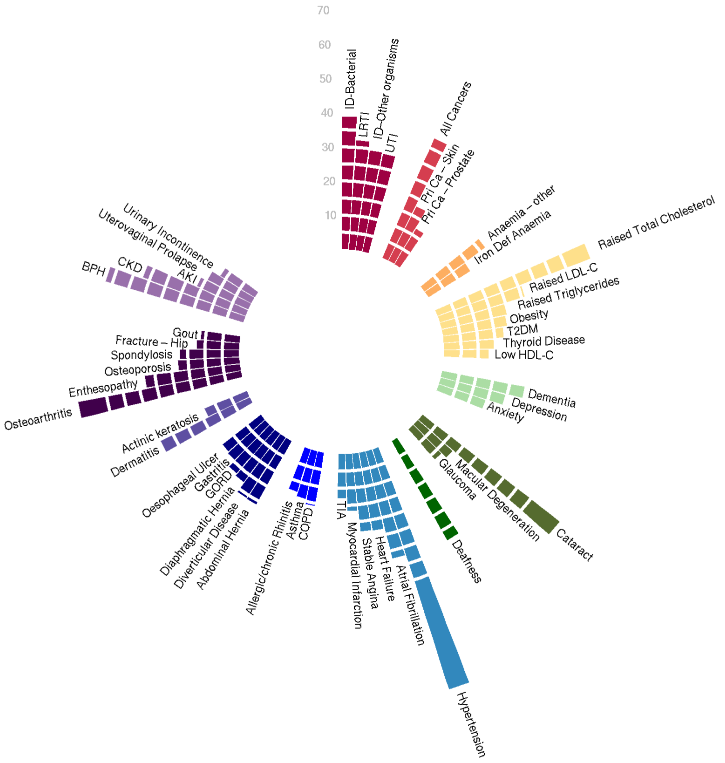

Supplementary Figure S4. Histogram of age at first record for thalassaemia and cystic fibrosis. The x-axis represents 10 year age bins, the y-axis represents frequency counts.

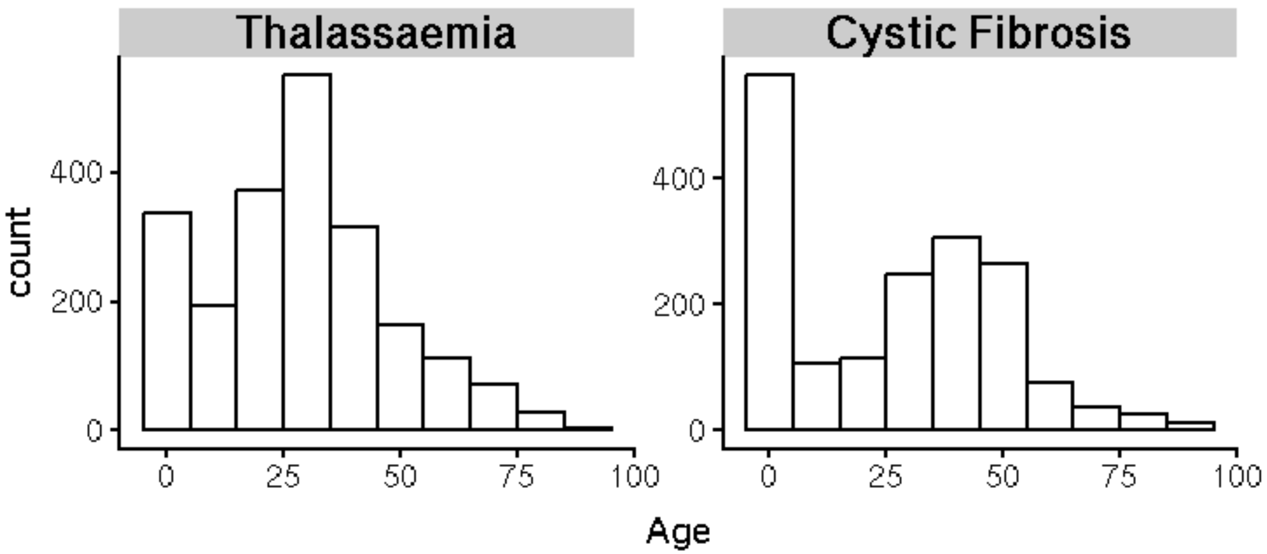

References

[1] GBD 2017 Disease and Injury Incidence and Prevalence Collaborators. Global, regional, and national incidence, prevalence, and years lived with disability for 354 diseases and injuries for 195 countries and territories, 1990–2017: a systematic analysis for the Global Burden of Disease Study 2017. Lancet 2018;392:1789–858. doi: http://dx.doi.org/10.1016/S0140-6736(18)32279-7.

[2] Barnett K, Mercer S, Norbury M, Watt G, Wyke S, Guthrie B. The epidemiology of multimorbidity in a large cross-sectional dataset: implications for health care, research and medical education. Lancet 2012;380:37-43.

[3] Kay J, Gawkrodger DJ, Mortimer MJ, Jaron AG.The prevalence of childhood atopic eczema in a general population. J Am Acad Dermatol. 1994; 30:35–39

[4] Montnemery P, Nihlén U, Göran Löfdahl C, Nyberg P, Svensson Å. Prevalence of self-reported eczema in relation to living environment, socio-economic status and respiratory symptoms assessed in a questionnaire study. BMC Dermatology. 2003;3:4. doi:10.1186/1471-5945-3-4.

[5] Stewart WF, Roy J, Lipton RB. Migraine prevalence, socioeconomic status, and social causation. Neurology. 2013;81(11):948-955. doi:10.1212/WNL.0b013e3182a43b32.

[6] Singleton N, Bumpstead R, O'Brien M, et al. (2001) Psychiatric Morbidity Among Adults Living in Private Households, 2000. London: The Stationery Office.

[7] https://digital.nhs.uk/data-and-information/publications/statistical/quality-and-outcomes-framework-achievement-prevalence-and-exceptions-data/quality-and-outcomes-framework-qof-2014-15. Accessed 20 December 2018.

[8] Action on Hearing Loss, 2015. Hearing Matters. London. https://www.actiononhearingloss.org.uk/-/media/ahl/documents/research-and-policy/reports/hearing-matters-report.pdf. Accessed 20 December 2018.

[9] Davis AC, 1995. Hearing in adults. London: Whurr.

[10] Public Health England. (2017) Tuberculosis in England: 2017. Public Health England, London. https://assets.publishing.service.gov.uk/government/uploads/system/uploads/attachment_data/file/686185/TB_Annual_Report_2017_v1.1.pdf. Accessed 20 December 2018.

[11] https://statistics.blf.org.uk/asthma. Accessed 20 December 2018.

[12] Ruhl CE, Everhart JE. Risk factors for inguinal hernia among adults in the US population. Am J Epidemiol. 2007 May 15. 165(10):1154-61.

[13] Primatesta P, Goldacre MJ. Inguinal hernia repair: incidence of elective and emergency surgery, readmission and mortality. Int J Epidemiol. 1996 Aug;25(4):835-9.
